# CRISPR Technology: Transforming the Future of Medicine and Diagnostics

**DOI:** 10.1021/acs.biochem.5c00480

**Published:** 2025-11-24

**Authors:** Kavita A Iyer, Rumiana Tenchov, Leilani M Lotti Diaz, Preeti Jain, Trupti Thite, Yi Deng, Qiongqiong Angela Zhou

**Affiliations:** 1 A Division of the American Chemical Society, CAS, Columbus, Ohio 43210, United States; 2 ACS International India Pvt., Ltd., Pune 411044, India

**Keywords:** CRISPR, landscape analysis, Casgevy, gene therapy, gene editing, CRISPR diagnostics

## Abstract

In this report, we examine the extensive research landscape of CRISPR with an emphasis on CRISPR therapeutics and showcase our results from an in-depth analysis of the most up-to-date scientific information consisting of more than 53,000 publications encompassing academic journal articles and patents, spanning nearly three decades, extracted from the CAS Content Collection. Our analysis indicates that cancer and infectious diseases are the most explored in the context of CRISPR. Identified gene targets associated with CRISPR-related publications are led by TP53, c-myc, and hemoglobin beta subunit (HBB). Among the many delivery methods, adeno-associated vectors (AAVs) appear to be highly explored. With >140 CRISPR-based therapeutics in the clinical development pipeline and billions of dollars in investment, the field of CRISPR continues to evolve rapidly. We also briefly discuss the ethical implications of CRISPR technology. While some fundamental challenges persist, the future of CRISPR is undoubtedly bright.

## Graphical abstract

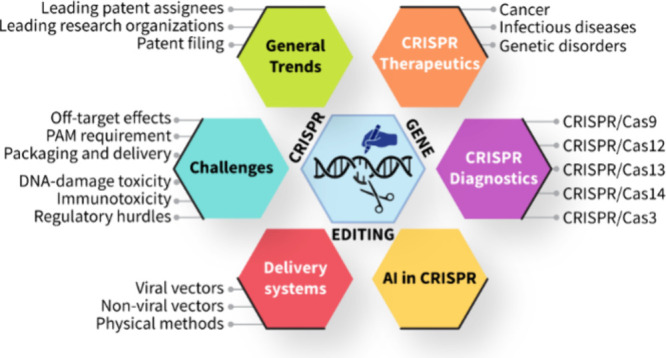

## Introduction

Clustered Regularly Interspaced Short Palindromic Repeats (CRISPR) and CRISPR-associated proteins (Cas) have revolutionized the field of genetic engineering and therapeutic development.^1–4^ Originally discovered as an adaptive immune mechanism in bacteria, CRISPR/Cas systems have been harnessed to enable precise and efficient genome editing in a variety of organisms.^5–7^ This powerful technology offers unprecedented opportunities for advancing our understanding of genetic diseases, developing novel therapies, and potentially curing previously intractable conditions.

CRISPR/Cas systems were first identified in bacteria and archaea as a defense mechanism against viral infections.^8,9^ The system works by capturing snippets of DNA from invading viruses and storing them in the bacterial genome. When the same virus attacks again, the bacteria produce RNA segments from the CRISPR sequences to target the viral DNA, guided by the Cas proteins, which then cut the DNA, neutralizing the threat.

This natural mechanism has been adapted for use in gene editing.^1,10^ The most commonly used system, CRISPR/Cas9, involves a guide RNA (gRNA) that matches the target DNA sequence and the Cas9 enzyme, which acts as molecular scissors to cut the DNA at the desired location. This break in the DNA can then be repaired by the cell’s natural repair mechanisms, allowing for the insertion, deletion, or modification of genes.^11,12^

Since its adaptation for gene editing, CRISPR technology has rapidly advanced. Researchers have developed various modifications of the original CRISPR/Cas9 system to improve specificity, efficiency, and versatility. For example, CRISPR/Cas12 and CRISPR/Cas13 target different nucleic acids, expanding the range of possible applications.^13–15^ Base editing techniques allow for precise conversion of single DNA bases without introducing double-strand breaks, reducing the risk of unwanted mutations.^16,17^ Prime editing represents a more recent advancement that combines aspects of CRISPR and reverse transcriptase to directly write new genetic information into a DNA site without causing double-strand breaks.^18,19^

The potential therapeutic applications of CRISPR are vast and encompass a wide range of diseases. Monogenic disorders, i.e., diseases caused by mutations in a single gene, such as sickle cell anemia, cystic fibrosis, and Duchenne muscular dystrophy, are prime targets for CRISPR-based therapies. Early clinical trials have shown promise in correcting these genetic defects.^20,21^ CRISPR is also being explored to enhance cancer immunotherapy by editing immune cells to better recognize and attack cancer cells. It is also being used to identify and validate new drug targets.^22–24^ CRISPR has potential applications in combating viral infections, such as HIV, by targeting and disabling viral DNA within the host genome.^25–27^

The future of CRISPR therapeutics is bright, with ongoing research aimed at overcoming current limitations and expanding its applications. Innovations such as CRISPR-based diagnostics,^28,29^ CRISPRa/i (CRISPR activation/interference for gene regulation),^30,31^ and combination therapies hold promise for broadening the impact of this technology. CRISPR therapeutics represent a transformative advance in medical science, offering the potential to treat and even cure a wide array of diseases. As research progresses and challenges are addressed, CRISPR-based therapies are poised to become a cornerstone of precision medicine, revolutionizing how we approach genetic disorders and complex diseases.

In this paper, we give an overview of the research progress in CRISPR therapeutics by analyzing data from the CAS Content Collection,^32^ the largest human-curated collection of published scientific information, supporting comprehensive quantitative analysis of global research across parameters including time, geography, scientific discipline, application, disease, chemical composition, etc. Relying on the expertise and knowledge of our subject matter experts, we have analyzed the corpus of CRISPR-related publications to identify and highlight interesting trends in terms of protein targets often targeted using CRISPR, the co-occurrences between diseases and protein targets, prevalence of different CRISPR/Cas proteins, and leading commercial and noncommercial entities engaged in research related to CRISPR. Finally, we inspect clinical applications of CRISPR therapeutics and diagnostics with details of their development. The objective of this review is to provide a broad overview of the evolving landscape of current knowledge regarding CRISPR application in therapeutics and diagnostics, to outline challenges that lie ahead and evaluate growth opportunities to further efforts in this groundbreaking technology.

To fully understand CRISPR, it is essential to break down its components and the mechanism of its natural function in prokaryotes in order to exploit CRISPR to achieve genome editing capabilities in humans and other organisms. Please see the Supporting Information for CRISPR/Cas biology and mechanism (Figure S1) and the types of CRISPR/Cas systems (Figure S2 and Table S1).

## General Trends in CRISPR Research: Insights from the CAS Content Collection

Querying the CAS Content Collection for publications related to CRISPR and its role in therapeutic treatment, therapeutic development, and therapeutic discovery (shortened to CRISPR therapeutics in this manuscript), while filtering out all agriculture related documents (see the methods section for query and details), resulted in over 39,000 academic journal articles and over 14,000 patents spanning from 1995 to June 2024. Publications on this topic sharply rose in 2008 and have steadily increased ever since with an average growth rate of 54% in the past decade (2014–2023) (Figure 1). This total rise in publications is primarily led by academic journal articles; however, patents showed a larger average yearly growth rate of 72% in the past decade when compared to journals (50%), demonstrating an increase in commercial interest.

**Figure 1:**
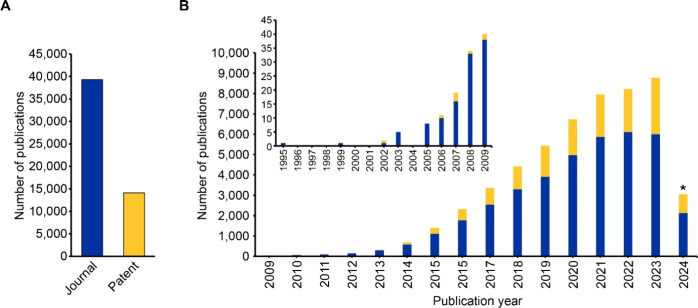
(A) Total number of journal and patent publications and (B) patent and journal publications through the years for the field of CRISPR including CRISPR therapeutics from 1995 to 2024. *Note that data for 2024 is incomplete due to time of data extraction and encompasses data for January to June.

We identified the top 100 journals containing the largest number of CRISPR therapeutics publications between 1995 and 2024. We then filtered out of this set the journals with the highest average citation per publication to provide data for Figure 2. The journal *Science*, with 262 publications, has the highest average citation (253 citations/publication) out of the top 100 journals by total publication (Figure 2). Topics of recently published and highly cited articles from this journal explore the following: the use of CRISPR/Cas9 screens to identify genes that could protect against copper-induced cell killing;^33^ the development of astrocyte-specific CRISPR/Cas9-based gene knockdown to reduce the expression of astrocyte morphology genes related to Alzheimer’s disease risk and other central nervous systems disorders;^34^ and the combination of fluorescence image-enabled cell sorting with CRISPR-pooled screens to identify regulators of the nuclear factor κB (NF-κB) pathway, quickly completing genome-wide image-based screens (9 h).^35^

**Figure 2:**
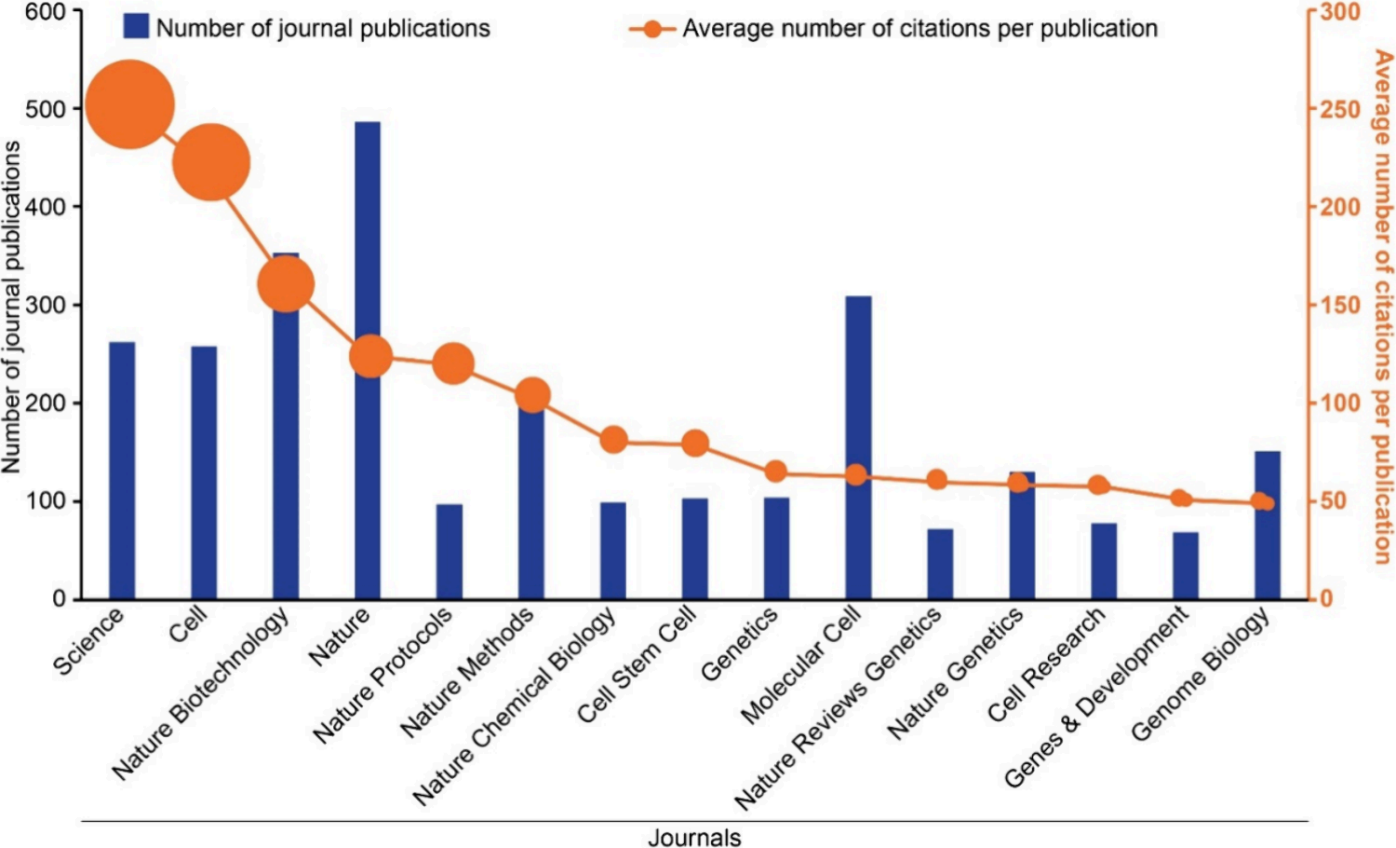
Leading scientific journals in the field of CRISPR based on research output (number of journal publications) and impact (average number of citations per publication) data from the CAS Content Collection for the period 1995–2024. Note that data for 2024 is incomplete due to time of data extraction and encompasses data for January to June.

*Cell*, the most known and oldest journal under Cell Press, comes in second place when it comes to citations with 220 citations/publications and 258 publications. Two recent publications in this journal with a high number of citations discuss the development and application of engineered DNA-free virus-like particles that efficiently package and deliver base editor or Cas9 ribonucleoproteins in vivo by overcoming cargo packaging, release, and localization bottlenecks^36^ and the use of genome-scale Perturb-seq targeting all expressed genes with CRISPRi across >2.5 million human cells for the generation of information-rich genotype–phenotype maps.^37^

Out of the top 15 journals shown in Figure 2, seven are owned by Springer Nature. The journals *Nature Biotechnology*, with a total of 353 publications, and *Nature*, with a total of 486 publications, come in third and fourth places with 161 citations/publication and 124 citations/publication, respectively. In addition, our data also shows that *Nature Communications* is the journal with the most publications on the topic of CRISPR therapeutics with 1,220 publications (Figure S6). Examples of publications from *Nature Biotechnology* cover topics like the design of an optimized Un1Cas12f1 and its application as a miniature CRISPR system that fits into the adeno-associated virus,^38^ new technologies to address challenges and allow biologically targeted mRNA therapeutics,^39^ and a prime editing-based method that achieves higher precision than CRISPR–Cas9 and sgRNA pairs in programming genomic deletions.^40^ Some examples of recent highly cited publications from the journal *Nature* report the use of CRISPR to conduct a genome-wide CRISPR knockout screen in glioblastoma to systematically identify potential resistance pathways to CAR-T cell cytotoxicity in solid tumors,^41^ the use of CRISPR-mediated targeting to identify mediators of Hopx induction (a transcriptional regulator) by β-hydroxybutyrate (BHB) and identify a BHB-triggered pathway regulating intestinal tumorigenesis,^42^ and provide molecular insight into the underlying structural mechanisms that cause off-target effects of Cas9 and a proof of concept for the design of Cas9 variants that reduce off-target DNA cleavage while retaining efficient cleavage of on-target DNA.^43^

We then looked at which organizations are leading academic research in the field of CRISPR therapeutics. If only taking into consideration the number of publications (Figure S7), the University of California, the Chinese Academy of Sciences, and Harvard University take the lead. Combination of research output (number of journal publications) and its impact (average citation per publication) reveals a different list (Figure 3) with Massachusetts General Hospital, Massachusetts Institute of Technology (MIT) and Harvard University as the leaders. Analyzing the geographical distribution of these leading organizations indicate that a majority of them originate in the United States (Figure 3).

**Figure 3:**
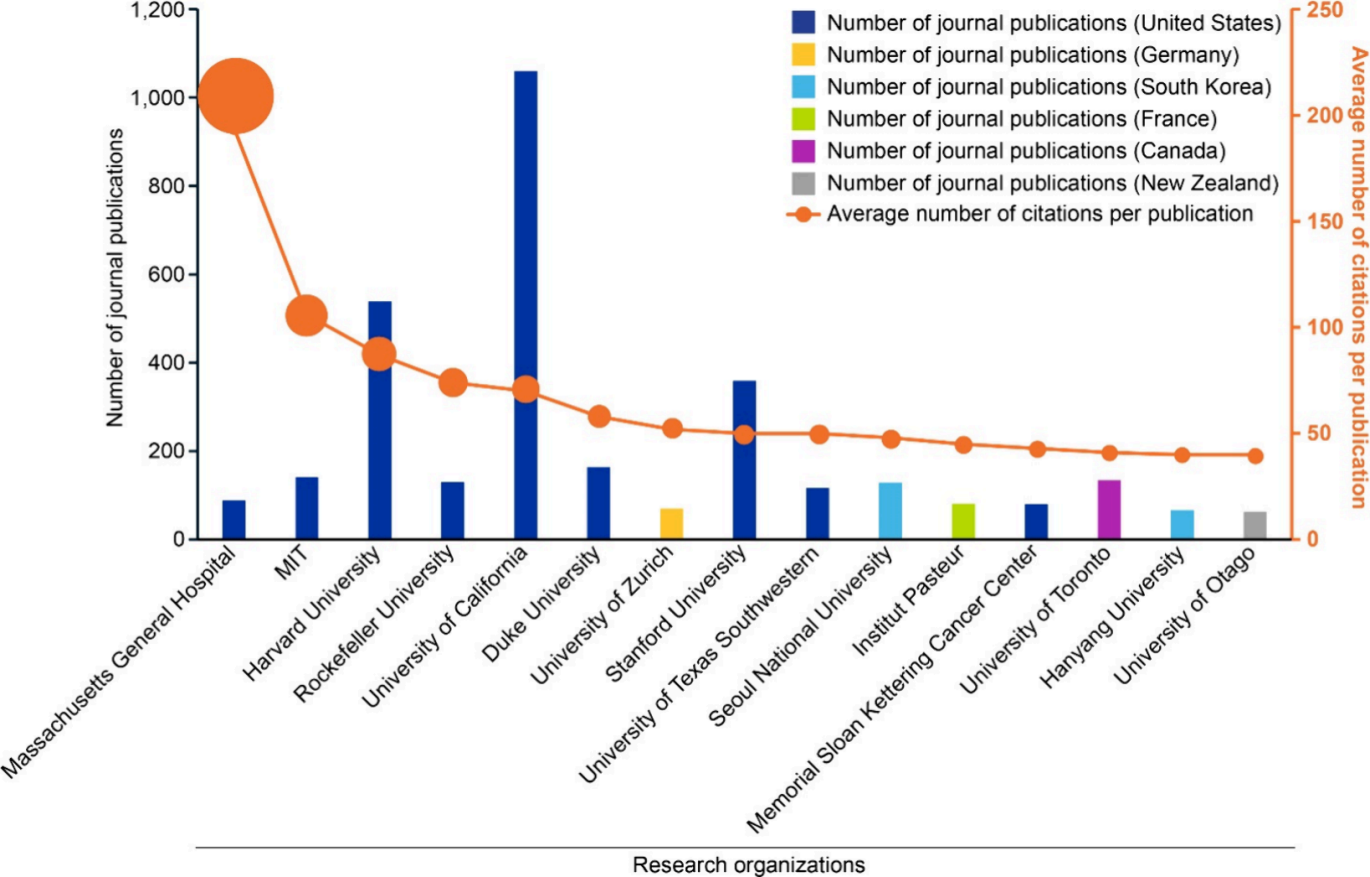
Leading research organizations in the field of CRISPR based on journal publication and citation data from the CAS Content Collection for the period 1995–2024. Note that data for 2024 is incomplete due to time of data extraction and encompasses data for January to June.

Taking a look into recent publications from the Massachusetts General Hospital, we observed the use of CRISPR: as a screening strategy to connect genes to detailed bioenergetic phenotypes in mitochondrias;^44^ to elucidate how Galectin 3 (Gal3) contributes to uterine serous carcinoma by using CRISPR/Cas9-mediated Gal3-knockout (KO) alongside a Gal3 inhibitor to evaluate Gal3′s impact on cell function;^45^ and to target PMS1 to reduce somatic expansion of the Huntington’s disease-associated CAG repeat.^46^ Examples of recent publications by MIT discuss using Cas9-assisted biological containment of a genetically engineered human commensal bacterium that could be used as a way to bring genetically modified microorganisms into biomedicine in a safe manner,^47^ and to examine effects of several simultaneous gene expression perturbations on growth using an *Escherichia coli* model.^48^ Finally, recent publications from Harvard University report the use of CRISPR technology: for germline mutagenesis to achieve genetic sterilization of male *Anopheles gambiae*, a species of malaria-carrying mosquitoes;^49^ to reveal a druggable pocket in STT3A, a subunit of oligosaccharyltransferase complex OST-A, whose inhibition blocks lipopolysaccharide signaling to NF-κB;^50^ to investigate the role of the progesterone receptor membrane component 1 (PGRMC1) in progesterone signaling at the maternal–fetal interface by knocking out PGRMC1 in JEG3 cells;^51^ and the use of CRISPR-corrected isogenic controls in research on human induced pluripotent stem cell lines.^52^

A look at patents in the field of CRISPR therapeutics, both submitted and approved patents, separated into commercial and noncommercial entities, are shown in Figures 4 and 5, respectively. When it comes to commercial assignees, Regeneron Pharmaceuticals in the U.S., CRISPR Therapeutics from Switzerland, and Shandong Shunfeng Biotechnology in China emerge as leaders among other key players. Overall, we observe that a majority (10 out of 15) commercial assignees among the top 15 are located in the U.S. Unlike commercial patents, Chinese and American academic and research institutions have a closer ratio (9:6, respectively) of dominance. For discussion of patent activity data in the field of CRISPR therapeutics please, see the Supporting Information (Figure S3).

**Figure 4:**
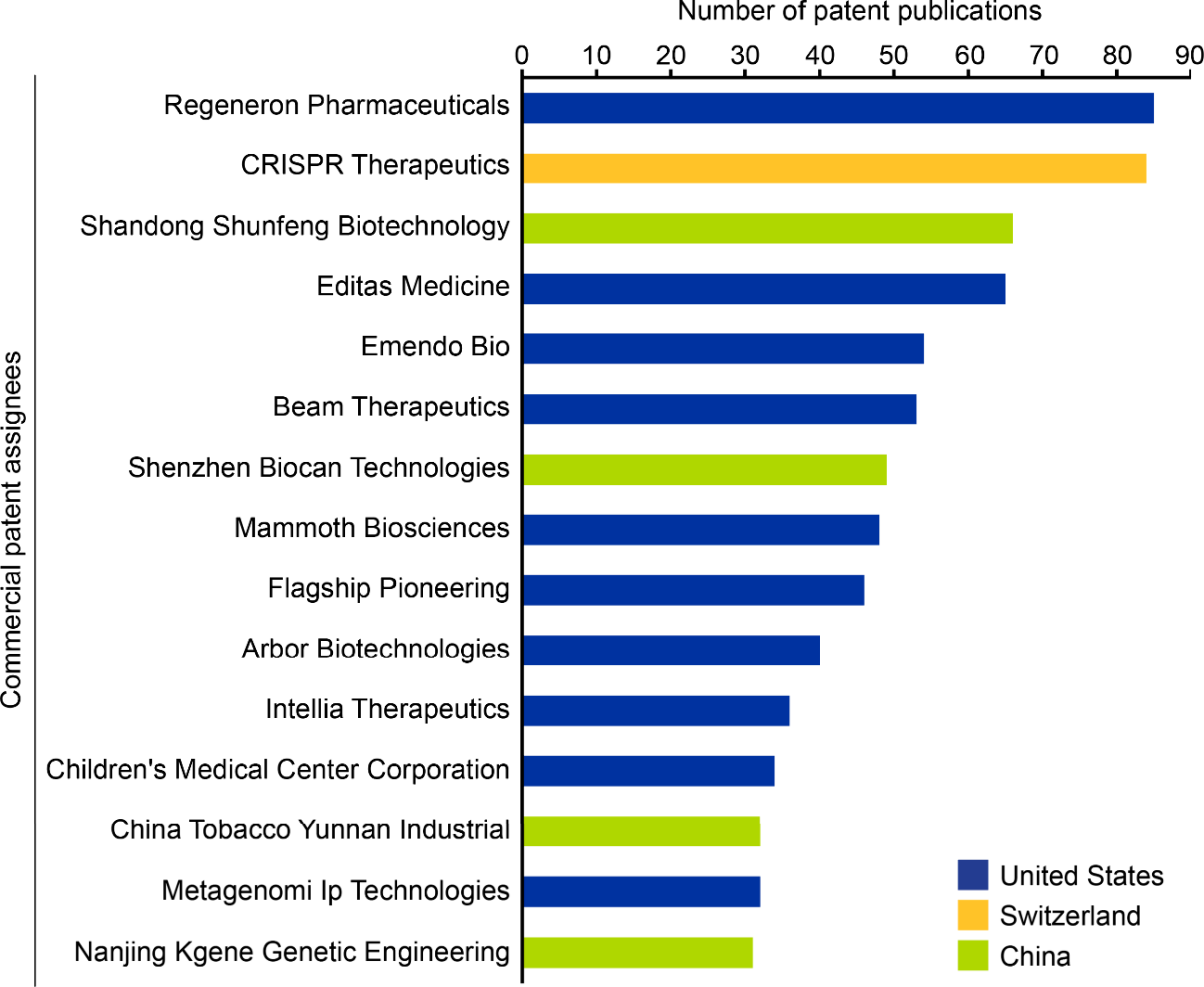
Leading commercial patent assignees in the field of CRISPR in terms of number of patent publications between 1995 and 2024 based on data from the CAS Content Collection. The bars have been color coded to indicate geographical location. Note that data for 2024 is incomplete due to time of data extraction and encompasses data for January to June.

**Figure 5:**
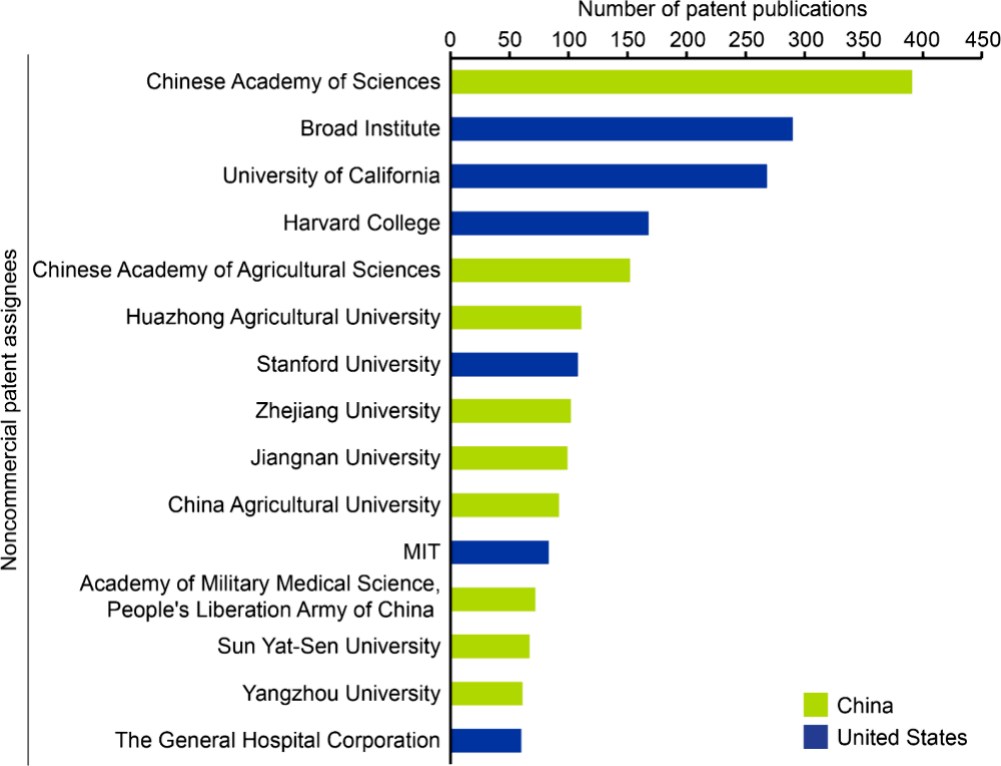
Leading noncommercial patent assignees in the field of CRISPR in terms of number of patent publications between 1995 and 2024 based on data from the CAS Content Collection. The bars have been color coded to indicate geographical location. Note that data for 2024 is incomplete due to time of data extraction and encompasses data for January to June.

A more detailed look into the top three commercial assignees and their recent submitted patents was merited. Regeneron Pharmaceuticals, an American biotechnology company, has recently published various patents on the use of CRISPR for the identification and treatment of liver disease,^53–56^ as therapeutics for c9orf72 repeat expansion disease,^57,58^ and for the treatment of ophthalmic diseases^59,60^ and metabolic disorders.^61,62^ Some other examples of recent patents include a CRISPR SAM biosensor cell line and their methods of use,^63^ and CRISPR/Cas methods and compositions for knocking out a C5 locus or gene.^64^ The Swiss–American biotechnology company, CRISPR Therapeutics, is known for its collaboration with Vertex Pharmaceuticals in creating the first-ever approved CRISPR/Cas9 gene-edited therapy known as CASGEVY.^65^ CASGEVY, also known as exagamglogene autotemcel, is a one-time therapy for sickle cell disease and β-thalassemia.^66^ Recent patents by CRISPR Therapeutics include the use of CRISPR for producing: CAR-T cells,^67–70^ genetically engineered immune cells,^71–76^ methods for differentiating stem cells into NK cells,^77,78^ and for in vivo editing of stem cells.^79^ Finally, Shandong Shunfeng Biotechnology, recently known for the development of the first gene-edited crop (soybean) approved by China, also ranks high among commercial patent assignees/entities.^80^ Some of their recent patents demonstrate various novel CRISPR/Cas systems and enzymes for targeting, editing, detecting mutations in, and cleaving nucleic acids.^81–86^ They have also recently published patents on efficient methods for detection of viruses^87^ based on CRISPR, including foot and mouth disease^88,89^ and African swine fever.^90^

A deeper look into recent patent publications from the leading noncommercial assignees (Figure 5) reveal the following:

1. The Chinese Academy of Sciences, a group of 124 individual research institutions,^91^ is a distinct leader with respect to the number of published patents in the field of CRISPR. A portion of their recent publications appear to be focused on use of CRISPR/Cas13 systems for targeting and treating diseases, such as SOD1-associated,^92,93^ UBE3a-associated,^94^ DMD-associated,^95^ and MECP2-associated^96^ diseases, nucleic acid detection based on CRISPR/Cas13a.^97–99^

2. The Broad Institute of MIT and Harvard, a biomedical and genomic research organization in Massachusetts, has recently patented CRISPR-associated transposase systems,^100–103^ CRISPR/Cas systems for gene editing mitochondria,^104,105^ and preparation of CRISPR/Cas systems comprising of adenine base editors,^106^ small novel Type V Cas polypeptides,^107^ and novel Cas5-HNH and Cas8-HNH polypeptides.^108^
3. The University of California, who as previously mentioned has the highest amount of journal publications, comes in third place when it comes to patents from noncommercial institutions. Some examples of recent patents discuss CRISPR/Cas effector proteins^109^ and polypeptides^110,111^ for gene editing, the use of CRISPR/Cas systems for modifying eukaryotic cells^112^ and oocytes,^113^ and CRISPR/Cas-mediated RNA targeting for treating Huntington’s disease^114^

In the past decade, capital investment in the field of CRISPR technology has seen a remarkable increase with a sharp increase starting in 2018 and persisting until 2021 with investments exceeding a staggering USD 11 billion in 2021 (Figure S4A; PitchBook Data, Inc.; *Data has not been reviewed by PitchBook analysts.). An overwhelming majority of these investments involved companies originating in the United States (USA, 96%). Other key players in terms of geographical distribution, though of much smaller magnitude, included Switzerland (CHE), China (CHN), and Japan (JPN) (Figure S4B). For more information about commercial interest in CRISPR, please see the Supporting Information.

With the recent and ongoing surge in artificial intelligence (AI) and its application in a wide range of fields, interest in using AI in CRISPR has also seen an increase as exhibited by the growth in publications over the past decade (Figure S5). For a brief description of some of the AI models developed for CRISPR, please refer to Supporting Information.

## CRISPR Therapeutics

The concept of gene therapy was introduced by Friedmann and Roblin back in 1972.^115^ ZFN (zinc finger nucleases) and TALEN (transcription activator-like effector nucleases) were then developed as mainstream tools to evaluate the possibility of targeting or editing genes to cure diseases. Both these methods require complex design strategies and can tolerate only a small number of positional mismatches making development of successful and effective gene therapy challenging. With ZFN, it is difficult to target nonguanine (G)-rich sites, and for each TALEN monomer, 5′ targeted base must be a thymine (T).^116–119^ Later, CRISPR/Cas emerged as a new tool to edit genes, and since its discovery, it has been explored tremendously by researchers as a potential therapeutic approach for disorders, which were previously thought to be incurable or difficult to cure. These include certain types of cancers, infectious diseases, and various genetic disorders, among others. CRISPR/CAS is beneficial over earlier conventional gene therapy methods such as ZFN and TALENs as it is easy to engineer and can tolerate positional/multiple consecutive mismatches.^120^

CRISPR/Cas technology has various key applications in the field of therapeutics, the most apparent of which would be to correct or replace the mutated or disease-causing gene(s). CRISPR/Cas-based gene therapy can be delivered in two modes—*in vivo* and *ex vivo*. For the *in vivo* approach, any viral or nonviral vector with the packaged CRISPR/Cas system is injected directly into the patient’s body, whereas, for the *ex vivo* approach, cells are first extracted from the patient, followed by growing them in the laboratory setup where the gene editing process is carried out and eventually the genetically altered cells are injected back into the patient’s body.^121^

Apart from the therapeutic application, CRISPR/Cas is often used in the functional genomics field to identify gene targets associated with certain diseases. Researchers can create gRNA libraries that target different genes in cell lines or animals and can further note the disruptions leading to phenotypic changes. This allows identification of candidate target genes involved in disease mechanism as well as potential therapeutic targets. CRISPR also enables high-throughput screening of genes in a fast and efficient manner. It is possible to establish experiments using pooled CRISPR libraries to screen thousands of genes simultaneously to discover their functions and understand their effects on various biological and pathological processes. Such high-throughput libraries are being constructed and explored particularly in cancers paving the way of using CRISPR in personalized medicine.^122^ Furthermore, CRISPR can also be used to create animal models for many diseases, helping researchers understand the molecular mechanisms of those diseases and eventually serving as an excellent tool during early stage drug discovery by enabling identification of therapeutic targets.^123^

As of today, numerous CRISPR-based therapeutics are in the preclinical stage of development, and many are undergoing clinical trials to validate their safety and efficacy for diverse disease conditions, as discussed further in this article (*CRISPR Therapeutics: Candidates in the Developmental Pipeline*). In December 2023, the first CRISPR/Cas9-based gene editing therapy got approval by the U.S. Food and Drug Administration (FDA) for the treatment of patients with transfusion-dependent β-thalassemia. The same therapy was approved in Europe in November 2023 for sickle cell disease and transfusion-dependent β-thalassemia.^127–129^

To gain insight and to understand the current trend in CRISPR therapeutics research, we explored the data from the CAS Content Collection and performed a quantitative analysis. Highlighted in Figure 6 are potential gene targets with the highest publication frequency in the CRISPR data set (journals and patents from 1995 to 2024). *TP53*, *c-myc*, and hemoglobin beta subunit (*HBB*) genes were the top three occurring genes identified. It is important to note that while TP53 is the most frequently mentioned gene in our data set, it is not always referenced specifically as a CRISPR target. As per Figure 7, the publication trend for genes such as c-myc, HBB, and CDKN2A show a steady increase while TP53 has shown a rapid increase over the past few years.

**Figure 6:**
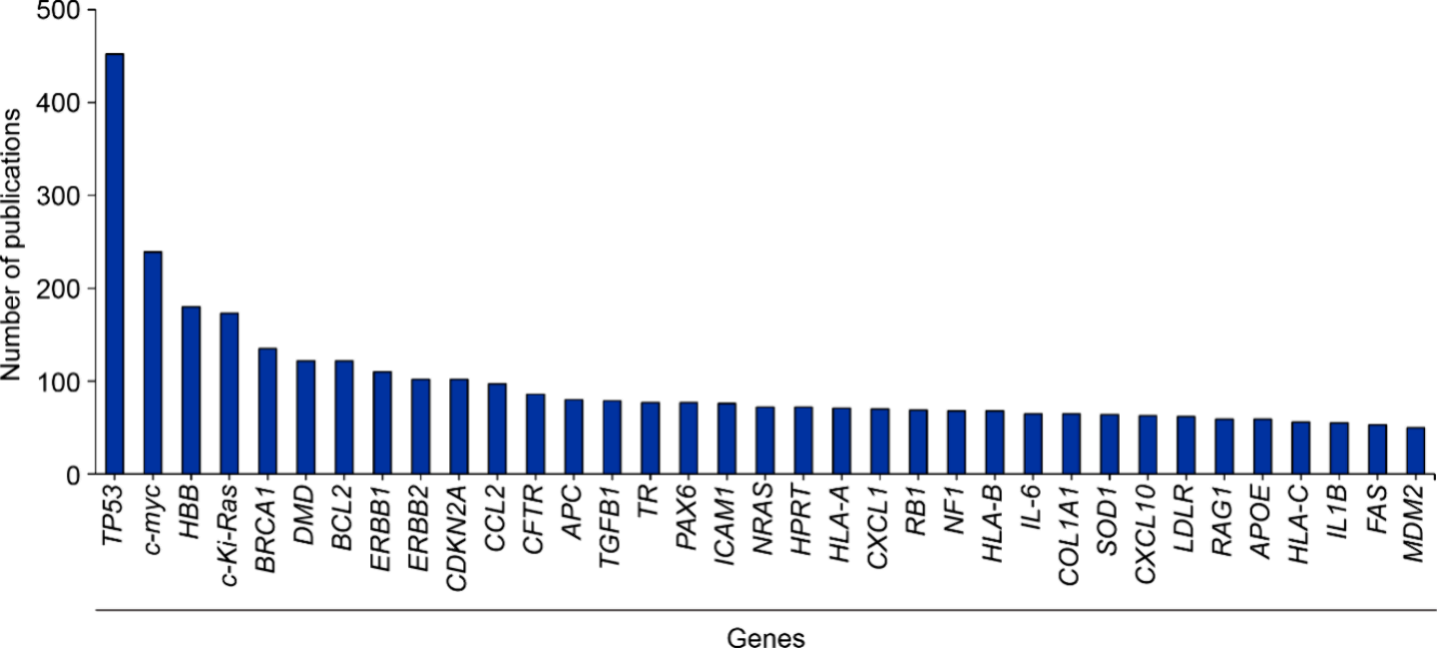
Publication frequencies of potential gene targets occurring in the CRISPR data set retrieved from the CAS Content Collection. Data includes patent and journal publications for the period 1995–2024 and is based on CAS indexing. Note that data for 2024 is incomplete due to time of data extraction and encompasses data for January to June.

**Figure 7:**
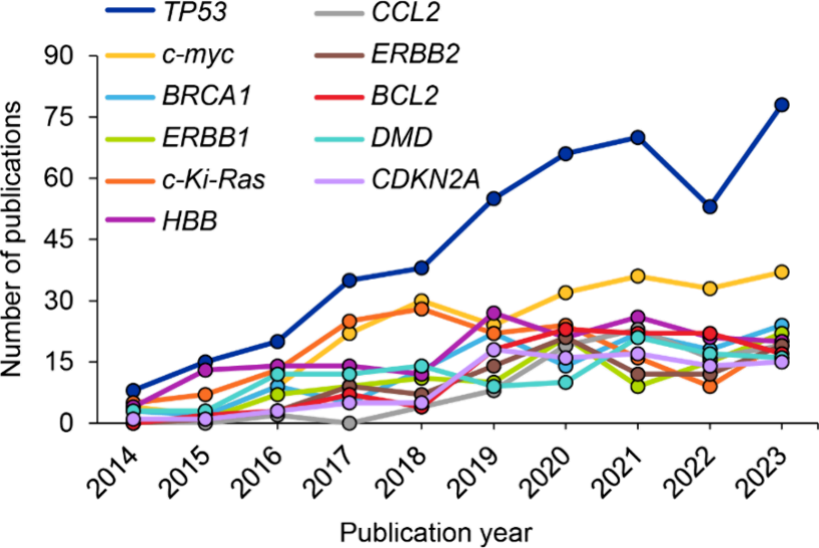
Time trends of some of the most highly occurring potential gene targets in the CRISPR data set retrieved from the CAS Content Collection. Data includes patent and journal publications for the period 2014–2023 and is based on CAS indexing.

As shown in Figure 8A, a majority of publications appear to be focused on cancer (35% of all journal articles and 24% of all patents explicitly mentioning diseases), followed by infectious diseases (25% and 22% of journal articles and patents explicitly mentioning diseases, respectively). Time trends of these diseases also show remarkable and consistent increase in number of CRISPR articles focused on cancer and infectious diseases after 2016 (Figure 8B and 8 **C**). Other broader categories of disease conditions observed in the data set were blood disorders, genetic disorders, nervous system disorders, cardiovascular diseases, respiratory diseases, immune diseases and metabolic disorders. In the following section we have discussed briefly how CRISPR/Cas technology is being utilized in the therapy targeted for these diseases with an emphasis on cancer, infectious diseases, blood disorders, genetic disorders (common as well as rare) and nervous system disorders.

**Figure 8:**
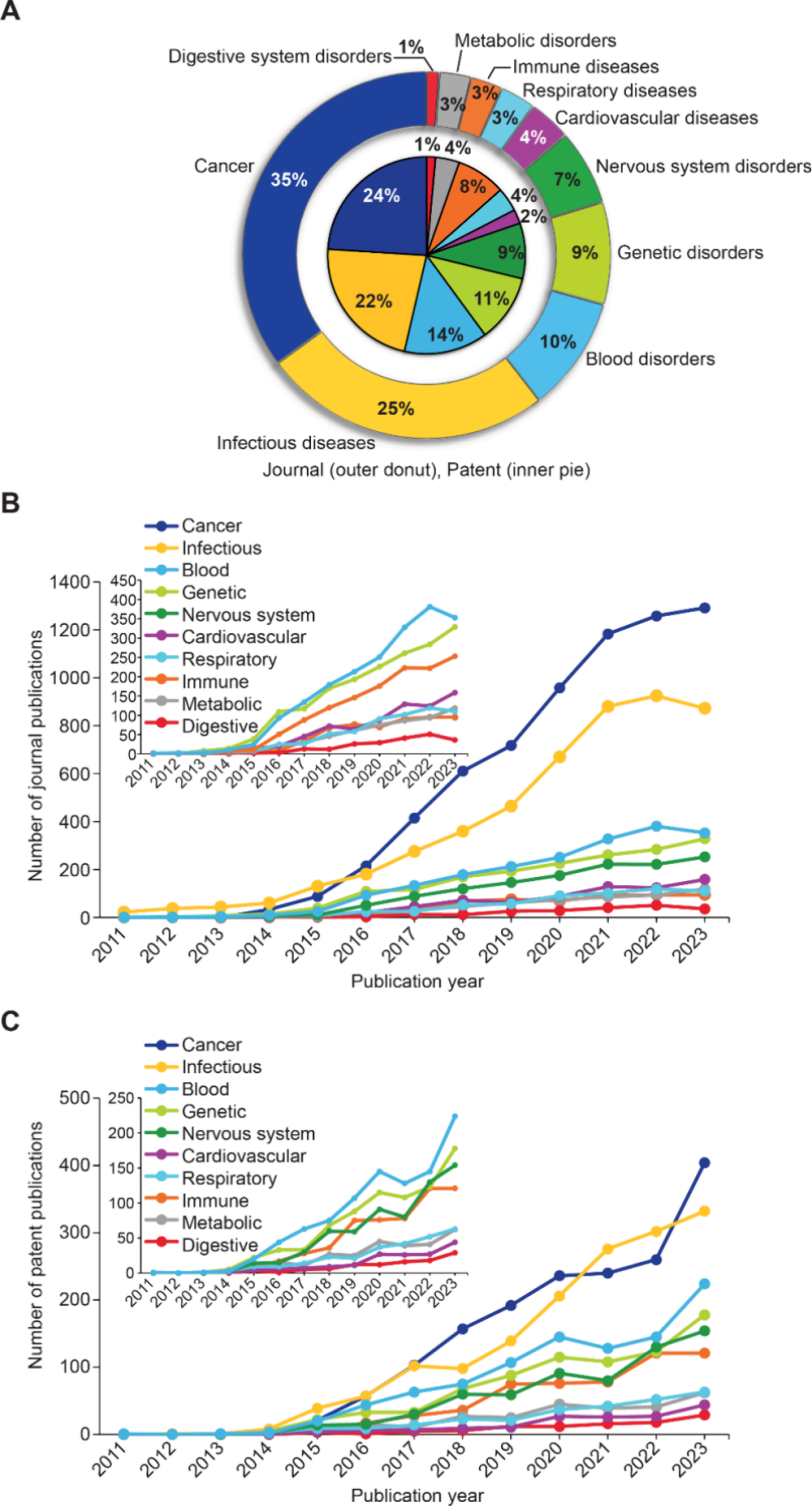
(A) Distribution and time trends for CRISPR (B) journal publications and (C) patents co-occurring with various disease conditions. Data includes journal and patent publications from the CAS Content Collection for the period 2011–2023.

### Cancer

Cancer is a multifaceted disease involving changes at the genomic, cellular, and eventually at the organismic level. Fundamentally, cancer originates in the genome, by mutations that either activate oncogenes or inactivate tumor suppressors. Dysregulation of the epigenome is another feasible way by which cells can become cancerous due to altered expression of certain genes involved in the DNA damage pathway or cell cycle pathway. At the cellular level, cancer results in altered metabolism, altered cell structure, and migration, which enables growth of cancer cells in unfavorable environments. Eventually, in the affected organism, cancer cells circumvent the immune defense mechanism of the host and coexist with normal cells. Understanding of all these complex genomic, cellular, and tissue level changes is crucial for the development of more effective treatment options and improving outcomes in cancer patients. CRISPR/Cas technology has had a significant impact on our understanding of cancer biology and is continuously driving new discoveries in the field.^127^

Supplementary Figure S8 shows the publication trend of CRISPR-related publications—journals and patents for different cancers subtypes (both solid cancer and hematological malignancies). Increase in journal publications was most evident for breast cancer, acute myeloid leukemia (AML), liver, lung, and rectal cancer. In line with the journal publications, patent publication trends show breast cancer, AML, and lung and liver cancer-related patents to be growing rapidly indicating potentially more commercialization efforts for these cancer types. Melanoma also shows a rapid increase in co-occurrence with CRISPR publications around 2022. Multiple gene candidates are being studied for cancers in the context of CRISPR, and Figure 9 shows co-occurrences between specific cancer types and genes found in the CRISPR data set retrieved from CAS Content Collection. A few key observations from this co-occurrence analysis are as follows:

1. Cancers such as breast, lung, rectal, prostate, and liver appear to co-occur more frequently with certain genetic targets than others.
2. Out of the more than 25 targets co-occurring frequently, ∼10 of them co-occur with more than one cancer type.
3. Besides *TP53*, other highly co-occurring genes include c-*K*_i_-Ras (*KRAS*), *c-myc*, *ERBB1*, and *BRCA1*.

**Figure 9:**
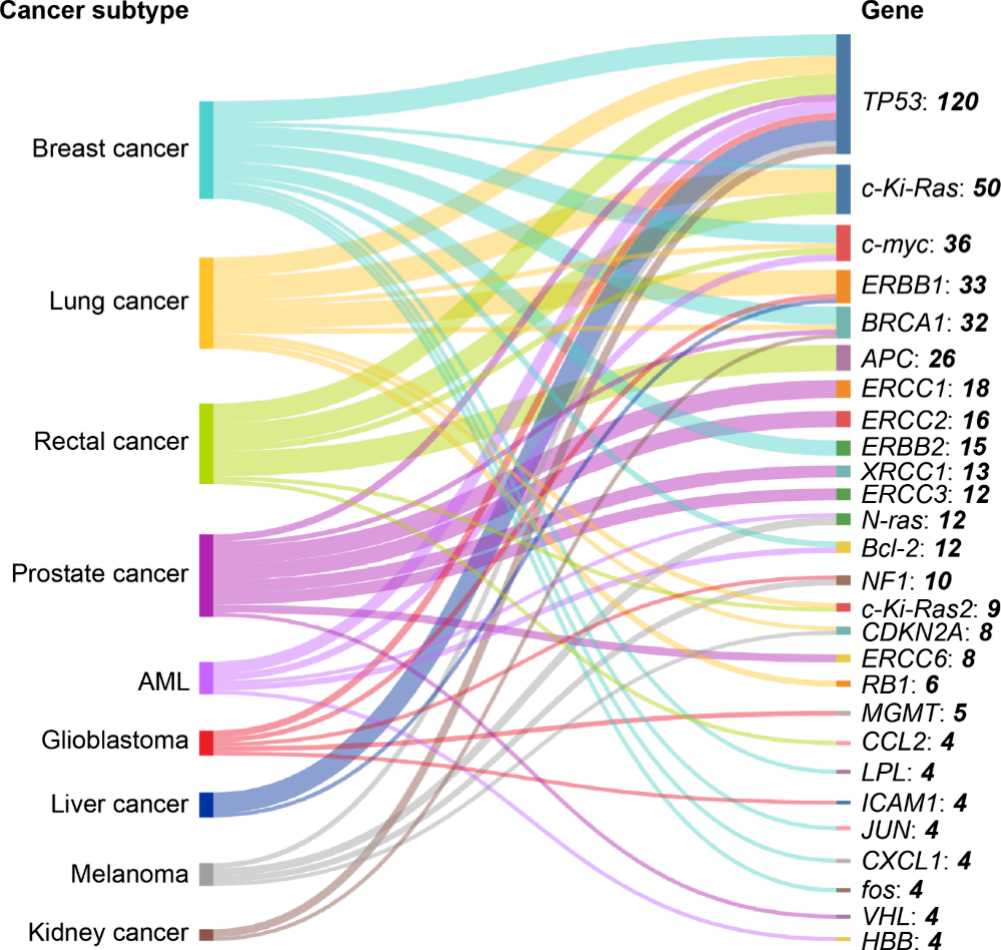
Co-occurrence of cancer subtypes (left column) with genes (right column) in the CRISPR data set retrieved from the CAS Content Collection. Data includes patent and journal publications for the period 1995–2024. Note that data for 2024 is incomplete due to time of data extraction and encompasses data for January to June.

In terms of diversity of genes co-occurring, breast, lung, rectal, and prostate cancers lead the way.

There are several approaches of using CRISPR/Cas technology in cancer therapy as discussed in the following.

#### Correcting Driver Mutations in Oncogenes or Tumor-Suppressor Genes

Oncogenes and tumor-suppressor genes play a critical role in the process of tumorigenesis. There are known driver mutations that either activate oncogenes or suppress tumor-suppressor genes, and both these phenomena disrupt the normal growth signaling pathways in cells, making them grow uncontrollably. Several studies have shown that by using CRISPR, it is possible to edit these mutations and revert the cancerous phenotype *in vitro* as well as *in vivo*.

Kim et al.^128^ used CRISPR/Cas9-mediated gene editing to target mutations in *KRAS* oncogene (*KRAS* G12C, G12D, and G12 V) in pancreatic cancer cells in mice and found that it inhibited cancer cell proliferation without affecting wild-type (WT) cells. In other studies, CRISPR/Cas9 was used to knock out another mutant oncogene, epidermal growth factor receptor (*EGFR*), resulting in the inhibition of proliferation of lung adenocarcinoma cell lines and considerable decline in tumor size and weight in xenograft mouse models.^129,130^

The *TP53* gene codes for a transcription factor and a well-known tumor suppressor that regulates multitude intracellular pathways involved in DNA damage repair, cell cycle arrest, apoptosis, and senescence.^131,132^ Mutations in *TP53* leading to its inactivation are involved in tumorigenesis and are found to be prevalent in more than 50% of human primary tumors.^133^ Majority of *TP53* mutations are missense mutations (around 80%) occurring due to guanine (G) to adenine (A) transitions, followed by cytosine (C) to thymine (T) transitions. These are clustered in the central DNA-binding region consisting of exons 3–5. Other known *TP53* mutations are truncating mutations, in-frame mutations, and slice site alterations. Since majority of mutations are missense, it opens great opportunities for the CRISPR/Cas9 system to correct single nucleotides.^134,135^

In prostate cancer cell lines, the *TP53* 414delC mutation was corrected to the wild-type *TP53* genotype by using the CRISPR/Cas9 system, thereby promoting apoptosis and preventing tumor proliferation.^135^

Zhan et al.^136^ have designed and constructed a genetic sensor that specifically detects WT-p53 expression in cells. Furthermore, by combining the p53 sensor with diphtheria toxin using the CRISPR/Cas9 system, they were able to specifically kill p53-deficient tumor cells.

Chira et al.^137^ proposed a novel and highly tumor-specific *TP53* delivery system based on CRISPR/Cas9 genome editing technology, which can be used to replace the mutant *TP53* in the tumor genome with a functional copy by homologous recombination, leading to sustained expression of p53 protein and tumor regression.

#### Modifying or Silencing Epigenetic Markers

The epigenome is a complex framework through which precise gene expression takes place and is one of the key regulators of cell fate, certain diseases, and aging. Editing the epigenome is a promising therapeutic approach in cancer.^138^ For epigenome editing, a “dead” Cas9 protein (dCas9) is used that lacks nuclease activity, and it is placed alongside an epigenetic effector domain. Based on fusion partners of dCas9, an exact epigenetic status can be achieved.^139^

Granulin (*GRN*), a growth factor and a potent pluripotent mitogen that promotes cancer progression by maintaining self-renewal of hepatic stem cancer cells, is upregulated in hepatoma tissues and is associated with decreased tumor survival. Wang et al. synthesized a set of dCas9 epi-suppressors to target *GRN* by tethering the C terminus of dCas9 with three epigenetic suppressor genes: *DNMT3a* (DNA methyltransferase), *EZH2* (histone 3 lysine 27 methyltransferase), and *KRAB* (the Krüppel-associated box transcriptional repression domain). The epigenetic knockdown of *GRN* (by altering promoter methylation status) led to the inhibition of cell proliferation, decreased tumor sphere formation, and reduced cell invasion.^140^

The mutated transcription factor *FOXA1* acts as an oncogene and is responsible for the onset and progression of prostate cancer. Zhou et al.^141^ identified a group of six *cis*-regulatory elements in the *FOXA1* regulatory plexus containing somatic single-nucleotide variants in primary prostate tumors. Deletion and repression of these *cis*-regulatory elements with the help of CRISPR/Cas technology significantly decreases *FOXA1* expression and prostate cancer growth.

Furthermore, CRISPR/Cas9-based epigenome editing was shown to successfully repress interleukin receptors (*IL1R1*) and tumor necrosis factor α receptor (*TNFR1*) in human adipose-derived stem cells and ovarian cancer cells, respectively.^142,143^ This approach may be used to control various kinds of inflammations that accelerate the growth of diverse types of cancers.

#### Assisting in Cancer Immunotherapy

Cancer immunotherapy, or immuno-oncology, is an approach to treat cancer by stimulating the body’s immune system to combat cancer cells. The major categories of immunotherapy include cytokine therapies, cancer vaccines, oncolytic virus therapies, immune checkpoint inhibitors, and adoptive cell transfer—which includes chimeric antigen receptor-T (CAR-T) cell therapy and natural killer (NK) cell therapy.^144^ One of the most promising applications of CRISPR/Cas9-mediated genome editing is the generation of CAR-T cells. In general, autologous T cells are collected and genetically engineered to attack cancer antigens *ex vivo* and subsequently transferred back into the patient. Zych et al. reported that the CRISPR/Cas9 system could be able to improve CAR-T cell function via interrupting the genes that code T cell inhibitory receptors or signaling molecules.^145^

CRISPR/Cas9 can also be used to create allogenic CAR-T cells, which can overcome mismatch of HLA typing a major limitation of autologous CAR-T cells.^146^ Various studies have attempted to create allogenic CAR-T cells by knocking out genes like beta-2 microglobulin (*B2M*), T cell receptor α subunit constant (*TRAC*), and programmed death 1 (*PD-1*).^147,148^ Using such an approach, it might be possible to create universal CAR-T cells derived from healthy donors that can be used for multiple patients helping tremendously to reduce the overall cost and time required to generate CAR-T based cell therapies. Table 1 elaborates various applications of using CRISPR/Cas9 system in CAR-T cell therapy.

**Table 1:** Applications of CRISPR/Cas9 in CAR-T Cell Therapy

**approach to modify CAR-T cells**	**modifications done in CAR-T cells**	**major outcome of the study**	**reference**
immune checkpoint blockade	knockout programmed death-ligand 1 (*PD-L1*) in primary T cells	enhanced CAR-T cytotoxicity	Su et al.^149^
	knockout cyclin-dependent kinase 5 (*CDK-5*) in CAR-T cells	reduced expression of PD-L1 and enhanced CAR-T cytotoxicity	Tu et al.^150^
	lymphocyte activation gene-3 (*LAG3*) knockout in CAR-T cells	strengthened T cell response and increased cytokine production	Zhang et al.^151^
	diacylglycerol kinase (*DGK*) knockout in CAR-T cells	stimulated CD3 signaling and increased resistance to the immunosuppressive factors TGF-β and prostaglandin E2	Jung et al.^152^
editing CAR-T cells to improve efficiency	CD40 ligand (*CD154*) expressing CAR-T cell	superior antitumor effects via NF-κB pathway	Kuhn et al.^153^
	inducible interleukin-12 (*IL-12*) secreting CAR-T cells	IL-12 secreting CAR-T cells attracted activated macrophages and eliminated antigen-loss tumor cells via tumor necrosis factor (TNF)-α mediated process	Chmielewski et al.^154^
	CXCR-2 expressing hepatocellular carcinoma (HCC)-targeting CAR-T cells	CXCR-2 expression stimulated the cohesion of CAR-T cells at the tumor site and ensured their migratory effect to the tumor microenvironment in HCC	Jin et al.^155^
improving durability and safety of CAR-T cells	disrupted *TET2* (Tet methylcytosine dioxygenase 2) promoter in CAR-T cells	*TET2* disrupted CAR-T cells exhibited higher antitumor activity *in vivo*	Fraietta et al.^156^
	CD7 and T cell receptor alpha chain (TRAC) expression lacking CAR-T cells, targeting T cell malignancies	modified CAR-T cells demonstrated efficacy against human T cell acute lymphoblastic leukemia (T-ALL) cell lines and primary T-ALL *in vitro* and *in vivo* without the induction of xenogeneic graft versus host disease (GvHD).	Cooper et al.^157^
	granulocyte-macrophage colony-stimulating factor (GM-CSF) knockout in CAR-T cells	GM-CSF is a major contributor in development of cytokine release syndrome (CRS), a well-known side effect of CAR-T cell therapy. GM-CSF KO CAR-T cells retained antitumor activity while reducing CRS.	Sterner et al.^158^

#### Targeting Mutations that Determine Drug Response or Susceptibility

Cancer cells can acquire resistance to targeted drugs or chemotherapy drugs by several mechanisms. Several mutations, mainly pathogenic single-nucleotide polymorphisms (SNPs), are known to develop during the course of therapy conferring resistance to cancer cells. One such example is the T315I mutation in the BCR-ABL kinase domain (threonine is substituted by isoleucine), which confers resistance against imatinib, a tyrosine kinase inhibitor used in treatment of BCR-ABL-positive hematological cancers. At the protein level, the mutation T315I results in a loss of a hydrogen bond, which is necessary for the binding of imatinib to the ATP-binding site of BCR-ABL, leading to significant reduction in efficacy of the drug.^159^ CRISPR-based editing offers a novel approach to silence such mutations and thereby restore drug efficacy.

EGFR T790M and TP53 R273H mutations are associated with gefitinib (a tyrosine kinase inhibitor) resistance in lung cancer patients. Yoon et al. showed that co-delivery of the adenine base editor (ABE) and EGFR- and TP53*-*SNP specific sgRNA via adenovirus resulted in accurate correction of the oncogenic mutations with high efficiency *in vitro* and *in vivo*. There was increased drug sensitivity and improved suppression of abnormal tumor growth in cells with altered EGFR and TP53 mutations as compared to control cells.^160^

In breast cancer cells, studies have been reported showing that genetically modified T47D and MCF7 breast cancer cells containing mutations in estrogen receptor 1 (ESR1) (Y537S and D538G) showed estrogen-independent growth and resistance to fulvestrant, raloxifene, and 4-hydroxytamoxifen (4-OHT) *in vitro*.^161–163^ In addition to addressing existing drug resistances, CRISPR can also be used to identify newer drug resistance mechanisms and mutations. Chen et al. showed that, in triple-negative breast cancer cells (HCC1937), the genetic ablation of ATPE1, a base excision repair enzyme, led to resistance to olaparib, a poly­(ADP-ribose) polymerase (PARP) inhibitor.^164^ In another study, CRISPR-based knockout of MAP3K1 in mutant PIK3CA breast cancer cells increased the proliferation rate and decreased sensitivity to AZD5363 (an AKT inhibitor) *in vitro* as well as *in vivo*.^165^

#### Inactivating Carcinogenic Viral Infections

The International Agency for Research on Cancer (IARC) has classified following viruses as carcinogens after comprehensive analysis: Epstein–Barr virus (EBV), hepatitis B virus (HBV), hepatitis C virus (HCV), Kaposi’s sarcoma herpes virus (KSHV), human immunodeficiency virus, type 1 (HIV-1), human T cell lymphotropic virus, type 1 (HTLV-1), and human papillomavirus (HPV). EBV, HPV, HTLV-1, and KSHV are classified as direct carcinogens, while HBV, HCV, and HIV-1 are considered indirect carcinogens (HBV and HCV cause chronic inflammation, and HIV-1 causes immune suppression).^166^

CRISPR/Cas technology has a promising role in targeting E6 or E7 genes in HPV, which are responsible for inducing cervical carcinoma. Kennedy et al. showed that the expression of a bacterial Cas9 RNA-guided endonuclease, together with sgRNAs specific to E6 or E7, induced cleavage of the HPV genome, resulting from inactivating mutations (deletions and insertions) into the E6 or E7 gene. This further induced p53 or retinoblastoma (Rb) protein, leading to cell cycle arrest and eventual cell death.^167^ In another study, CRISPR/Cas9 was used to target the promoter of HPV16 E6/E7 as well as E6 and E7 transcripts resulting in significant reduction in proliferation of cervical cancer cell line SiHa and reduced tumorigenesis in mouse models.^168^

The CRISPR/Cas9 system could successfully treat EBV-related cancers during the latent phase of EBV infections by targeting EBV viral genomes.^169^ CRISPR/Cas9 was shown to cause direct cleavage of the JCV genome, a small circular dsDNA that encodes for the viral early protein, T-antigen. CRISPR/Cas9 was used to stop viral replication in transformed human glial cells because of the inactivation of the T-antigen-coding genes, which are critical for directing viral reactivation and lytic infection.^170^

The following two approaches while not direct therapy approaches are still important tools in translational research as they help in understanding molecular mechanisms of various cancerous phenotypes, providing invaluable information during early phases of drug discovery:

#### Creating Tumor Models and Organoids

Transfecting of mouse embryonic stem cells with CRISPR/Cas9, sgRNA, and ±donor template promotes homology-directed repair (HDR) and enables development of efficient knockout or knock-in mouse models. CRISPR/Cas9 can also be used to develop inducible Cas9 mouse models to perform efficient somatic editing *in vivo*, with various organs as possible targets using either adeno-associated virus- (AAVs), lentivirus-, or nanoparticle-mediated sgRNA delivery.^171–173^

Heckl et al. used the CRISPR/Cas9 system via the lentiviral delivery method to revive several inactivated oncogenes in primary hematopoietic stem and progenitor cells (HSPCs) to generate leukemia models. The targeted genes were *TET2*, *RUNX1*, *DNMT3A*, *NF1*, *EZH2*, and *SMC3*.^174^

CRISPR/Cas9 technology has also been adopted to develop organoid tumor models. For example, organoid models for colon cancer were constructed *in vitro* by using CRISPR to introduce mutations in tumor-suppressor genes (*APC*, *TP53*, *SMAD4*, etc.) and modify oncogenes (*KRAS*, *PI3K*, etc.).^175^ Roper et al.^176^ established a protocol to induce site-directed tumors rapidly and efficiently in the distal colon of mice by utilizing colonoscopy-guided mucosal injection. This technique can be extrapolated to deliver viral vectors carrying Cre recombinase, CRISPR/Cas9 components, CRISPR-engineered mouse tumor organoids, or human cancer organoids to mice to model the adenoma–carcinoma–metastasis sequence of tumor progression.

#### Creating High-Throughput Genetic Screens

CRISPR-based high-throughput screening is a large-scale genetic loss-of-function experimental approach that facilitates discovery of key genes or gene sequences that correlate with a specific function or phenotype for a cell type, for example, resistance or sensitivity to a drug and susceptibility to environmental toxins, components of a cellular pathway or novel pathogenic biomarkers.^177,178^

Recently, using CRISPR screens, a compelling lethal interaction between the helicase-encoding *WRN* gene and microsatellite instability was identified.^179,180^ In immuno-oncology, the molecular mechanism of tumor immune evasion was explored, which included multiple factors like Ras signaling, antigen presentation, interferon, autophagy, and epigenetic remodeling.^181–183^ In another study, a CRISPR-based screening approach showed that depletion of neurofibromin, merlin, and the mediator complex component MED12 conferred resistance to vemurafenib, a B-Raf enzyme inhibitor, in B-RAF mutant melanoma cells.^178^

In the future, CRISPR/Cas9-based efficient and precise cancer models and high-throughput screens are likely to significantly promote functional cancer genomics research and accelerate the development of novel cancer therapies.

### Infectious Diseases

Infectious diseases were the second largest subset of publications in the CRISPR data set extracted from the CAS Content Collection. A total of 25% of all journal publications and 22% of all patent publications explicitly mentioning diseases were related to infectious diseases. There has been a steep increase over the past few years in number of publications on infectious diseases and CRISPR technology, especially marked for bacterial and viral infectious diseases (Figure S9).

CRISPR has emerged as a promising alternative to develop therapeutics against various pathogens by

1. targeting the pathogen genes required for replication, entry, or infecting the host cells or
2. altering host genes required by pathogens to cause infection or
3. modifying genes responsible for drug resistance or susceptibility^123,184,185^

CRISPR-based antimicrobials have a unique advantage over other conventional antimicrobials because they can destroy microbes based on their genomic sequence. This is particularly useful in situations where only a small number of microbes within a genus must be targeted and eradicated, which is tough to do with existing antimicrobial strategies.^186,187^

Table 2 enlists numerous studies conducted for exploring CRISPR-based therapeutics as antimicrobial agents.

**Table 2:** Examples of CRISPR/Cas9-Based Therapeutics As Antimicrobials

**pathogen**	**target genes of the pathogen**	**major outcome of the study**	**reference**
**Cas9**			
herpes simplex virus 1 (HSV-1)	HSV-1 genome was targeted using *Streptococcus pyogenes* Cas9 (SpCas9) mRNA and viral gene-targeting gRNAs (designated HSV-1-erasing lentiviral particles, HELP)	HSV-1 replication was blocked	Yin et al.^188^
hepatitis B virus (HBV)	the surface antigen (HBsAg)-encoding region of HBV, *in vitro* and *in vivo*	HBV replication and expression was inhibited	Zhen et al.^189^
hepatitis C virus (HCV)	HCV 5′ untranslated region involved in both translation of the viral polyprotein and replication of the viral RNA	HCV RNA transcription was inhibited	Price et al.^190^
human immunodeficiency virus (HIV)	edit integrated proviral DNA (long terminal repeats region)	HIV-1 expression was suppressed	Ebina et al.^191^
*Staphylococcus aureus*	virulence genes and antibiotic resistance genes	only the virulent *Staphylococcus aureus* was killed. By targeting antibiotic resistance genes, bacteria became more susceptible to existing treatment	Bikard et al.^192^
Mycobacterium tuberculosis	multiple genes of *Mycobacterium tuberculosis*	sequence-specific regulatory suppression in M. tb was observed	Choudhary et al.^193^
*Aspergillus fumigatus*	multiple genes of *Aspergillus fumigatus,* like those involved in drug resistance or rRNA processing or other essential functions	increased drug susceptibility and reduction in fungal growth was observed	Vyas et al.^194^
*Candida albicans*	CDR1 and CDR2 (members of the multigene drug efflux pump encoding family), responsible for drug resistance to azoles	by knocking out CDR1 and CDR2, the clinical strain of *Candida albicans* did not show hyper-resistance to fluconazole or cycloheximide	Vyas et al.^194^
**Cas3**			
*Clostridium difficile*	the genome of *Clostridium difficile* to create long-range deletions (packaged in bacteriophages)	bacteriophages containing the targeted CRISPR/Cas3 system killed *Clostridium difficile*	Selle et al.^195^

### Blood Disorders

The delivery of genome editing machinery by utilizing CRISPR/Cas technology to target blood cells possesses an interesting possibility to provide cure for patients with inherited monogenic blood diseases such as sickle cell anemia and β-thalassemia. The first U.S. FDA-approved CRISPR therapeutic, Casgevy, is an autologous gene therapy that edits the *BCL11A* gene, which helps in production of fetal hemoglobin. Eventually, this stops red blood cells (RBCs) from adopting their characteristic sickle shape.^196^ Other therapies for the treatment of sickle cell anemia and β-thalassemia include targeting the erythroid-specific enhancer region of the *BCL11A* gene and *HBG1*/*HBG2* genes and are currently undergoing clinical trials.^197^

β-Thalassemia is also associated with mutations in the *HBB* gene, particularly a point mutation in intron 2 that alters splicing. Xu et al. used TALENs and CRISPR/Cas9 to target the aberrant intron to restore *HBB* gene expression in induced pluripotent stem cells (iPSCs) *in vitro*, creating a potential opportunity for cell therapy through hemopoietic stem cell replacement.^198^

### Common and Uncommon Genetic Disorders

Among the many promising possibilities of using CRISPR-based therapeutics, their translational use in monogenic human genetic diseases has the potential to provide long-term therapy after a single treatment. Genetic disorders can be treated with the help of CRISPR by editing the defective (disease-causing) gene or by editing the enhancer or regulator of the defective gene. Numerous studies, which are summarized in the table below (Table 3), have shown promising results by using these two approaches.

**Table 3:** Examples of CRISPR-Based Therapeutics for the Treatment of Genetic Disorders

**disease**	**CRISPR target**	**approach**	**major outcome of the study**	**reference**
Duchenne muscular dystrophy	dystrophin gene (*DMD*)	single or multiplexed sgRNAs were developed to restore the dystrophin reading frame by targeting the mutational hotspot at exons 45–55 and introducing shifts within exons or deleting one or more exons	dystrophin expression is restored *in vitro*	Ousterout et al.^199^
Huntington's disease	Huntingtin gene (*HTT*)	HTT 5′ UTR was targeted	improper maturation of the transcript and reducing the expression of the disease-causing allele	Kolli et al.^200^
		a dual sgRNA approach was used *in vitro* to excise a 44kb promoter region upstream of a mutant *HTT* gene to silence its expression	expression of the Huntington's disease-causing variant was ablated	Shin et al.^201^
glaucoma	myocilin gene (*MYOC*)	Knocked down the expression of mutant *MYOC* in a mouse model of primary open-angle glaucoma	reduction of ER stress, lower intraocular pressure, and the preventability of further glaucomatous damage in mouse eyes was observed. The authors also demonstrated the feasibility of utilizing CRISPR/Cas9 in human eyes with glaucoma	Jain et al.^202^
hereditary tyrosinemia type I	fumarylacetoacetate hydrolase gene (*FAH*)	HDR-mediated point mutation correction in mouse hepatocytes.	a significant proportion of alleles were corrected	VanLith et al.^203^
Leber congenital amaurosis type 10 (LCA10)	centrosomal protein 290 gene (*CEP290*)	AAV5-based therapy (EDIT-101) encapsulates *Staphylococcus aureus* Cas9 (SaCas9) and two sgRNAs targeting genomic locations upstream and downstream of the intronic *CEP290* point mutation. The two sgRNAs enable cutting around the mutation to induce its removal or inversion	normal splicing of *CEP290* pre-mRNA was restored	Maeder et al.^204^
Noonan syndrome	leucine zipper like post translational regulator 1 gene (*LZTR1*)	intron 16 of *LZTR1* was targeted	the gene editing process could overcome the disease phenotype associated with Noonan syndrome-associated cardiomyopathy in iPSC-derived cardiomyocytes *in vitro*	Hanses et al.^205^
Angelman syndrome	UBE3A-ATS Inc.RNA	UBE3A-ATS Inc.RNA was targeted in cultured human neurons and in a mouse model of the disease	targeting of UBE3A-ATS ablated its function, leading to expression of the paternal *UBE3A* gene and rescuing the disease phenotype	Wolter et al.^206^
congenital muscular dystrophy type 1A (MDC1A)	laminin subunit alpha 1 gene (*LAMA1*)	CRISPR activator mediated gene upregulation	3.6-fold upregulation of *LAMA1* was observed	Kemaladevi et al.^207^
genetic deafness	transmembrane channel like 1 gene (*TMC1*)	non-homologous end joining (NHEJ)-mediated mutant Tmc allele disruption	deafness was prevented in mouse models up to one year post injection	György et al.^208^

### Nervous System Disorders

While accounting for a smaller fraction of CRISPR publications (Figure S8A), nervous system disorders still contribute about 7 and 6% of journal articles and patents in the field of CRISPR. Figure S10 further shows the breakdown of publication trend across various nervous system disorders—a key takeaway is that the rate of growth of publications in the field of CRISPR co-occurring with Alzheimer’s and Parkinson’s diseases has increased over the past decade, indicating interest from both academic researchers and commercial entities. CRISPR/Cas9 technology has gained popularity in the field of neurodegenerative diseases due to its short experimental duration and easy molecular engineering requirements. It is currently being extensively utilized for building disease models, identifying pathogenic genes through screening, and for targeted therapy.

Alzheimer’s disease (AD) is the most prevalent neurodegenerative disease characterized by memory deficits and cognitive decline. It is mainly characterized by two neuropathological features—the accumulation of extracellular amyloid β (Aβ) protein plaques and neurofibrillary tangles primarily composed of hyperphosphorylated Tau protein.^209,210^ Majority of cases of AD are known to be sporadic in nature; however, a small percentage of cases are familial (known as familial AD or FAD), caused by dominant autosomal mutations found in one of three genes: presenilin-1 (*PSEN1*), presenilin-2 (*PSEN2*), and amyloid precursor protein (*APP*).^211,212^

Sun et al.^213^ knocked out *PSEN1* genes using CRISPR/Cas9 in mouse neuroblastoma cells and observed decreased production of Aβ42 and Aβ40. Konstantinidis et al.^214^ suggest that the CRISPR/Cas9 approach can be used to selectively disrupt the PSEN1M146L allele responsible for AD and partly switch the abnormal Aβ42/40 ratio that leads to the development of the disease in carriers of this mutation. Ortiz-Virumbrales et al.^215^ demonstrated that CRISPR/Cas9 can correct neurons derived from the PSEN2N141I-mutated individual fibroblasts and can further normalize the Aβ42/40 ratio. This was shown to effectively restore the associated electrophysiological deficits.

Parkinson’s disease (PD) is the second most prevalent neurological disorder in humans, which is characterized by the progressive loss of dopaminergic neurons and significant decrease in dopamine levels as well as functional impairment of the motor circuit. Around 90% of PD cases are not linked to any known cause, while the remaining 10% have familial PD caused by mutations in specific genes like α-synuclein (*SNCA*), parkin RBR E3 ubiquitin protein ligase (*PRKN*), PTEN-induced kinase 1 (*PINK1*), and leucine-rich repeat kinase 2 (*LRRK2*).^216,217^

The missense mutation, Ala53Thr (A53T) in *SNCA*, is considered to be one of the most prominent risk factors for early-onset PD. Yoon et al.^218^ conducted a study where they deleted the A53T-SNCA gene using CRISPR/Cas9, which significantly improved conditions related to PD, such as the overproduction of α-synuclein, reactive microgliosis, dopaminergic neurodegeneration, and PD-associated motor symptoms.

There is significant research still ongoing in identifying novel biomarkers and mutations involved in the onset of AD and PD. Developing disease models is critical in understanding disease biology and pathology, and CRISPR has shown promising utility in the same. Few of the examples are cellular model of AD with disease-causing mutations in *APP* and *PSEN1*,^219^ mouse model for AD with tau knockout,^220^ and a monkey model for PD with *PINK1* deletion.^221^

## CRISPR Therapeutics: Candidates in the Developmental Pipeline

Over the past decade, CRISPR has made significant strides in clinical research, with numerous trials launched to explore its potential in therapeutics. As a result, in late 2023, the CRISPR-based therapeutic, Casgevy, was granted approval becoming the first ever in just 11 years which is truly a remarkable achievement. Casgevy (exagamglogene autotemcel), the CRISPR/Cas9 gene editing therapy for the treatment of patients with transfusion-dependent β-thalassemia and the treatment of sickle cell disease in patients aged ≥12 years with recurrent vaso-occlusive crises, was approved by the UK Medicines and Healthcare Products Regulatory Agency (MHRA) on 16 November 2023.^125^ The U.S. FDA approved Casgevy and Lyfgenia (lovotibeglogene autotemcel) for patients with sickle cell disease on 8 December 2023.^124^ Casgevy has also been approved by the European Medicines Agency (EMA) for sickle cell disease and transfusion-dependent β-thalassemia on 15 December 2023.^126^

To gain insights about ongoing preclinical and clinical trials on CRISPR technology, we retrieved data from Pharmaproject Citeline Clinical Intelligence (Figure 10). At present, there are 142 CRISPR therapeutics in different stages of development of which 10% are in phase I, 11% in phase II, and 1% in phase III clinical trials. A vast majority of CRISPR therapeutics (77%) are still in the preclinical stage of development. Listed in Table S2 are examples of CRISPR therapeutics in phases I–III with information about their gene and disease targets.

**Figure 10:**
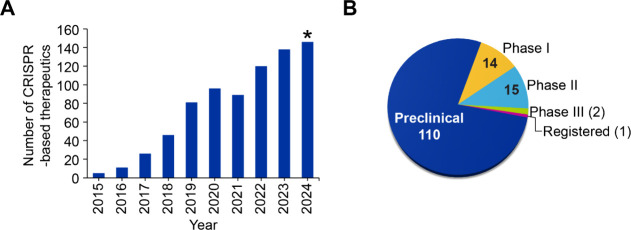
(A) Year-wise distribution of CRISPR-based therapeutics in preclinical and clinical trials. (B) Distribution of CRISPR-based therapeutics as per stage of development (preclinical, phase I, phase II, and phase III). Data retrieved from Pharmaproject Citeline Clinical Intelligence. Data for 2024 is partial and includes data until June 2024.

The range of disease conditions targeted by CRISPR-based therapeutics currently in the preclinical stages of development are wide—from rare genetic disorders and blood diseases to various forms of cancer and even infectious diseases such as HIV, tuberculosis (TB), and COVID-19. The data reveals that 25% of these therapeutics are focused on cancer (Figure 11), which consists of treatment for solid tumors (60%) and hematological malignancies (34%). CRISPR-edited CAR-T therapies are leading (57%) against hematological malignancies. However, some CAR-T cell therapies are also being developed for solid tumors (43%) with the help of CRISPR technology. Nkarta in collaboration with CRISPR Therapeutics is developing an allogeneic chimeric antigen receptor-natural killer (CAR-NK) cell therapy targeting CD70, using its off-the-shelf NK cell-based technology for the treatment of solid and hematological cancers. Other major disease groups targeted by CRISPR-based therapeutics that are currently under exploration include immunological (4%), respiratory (3%), and dermatological (1%) diseases (Figure 11).

**Figure 11:**
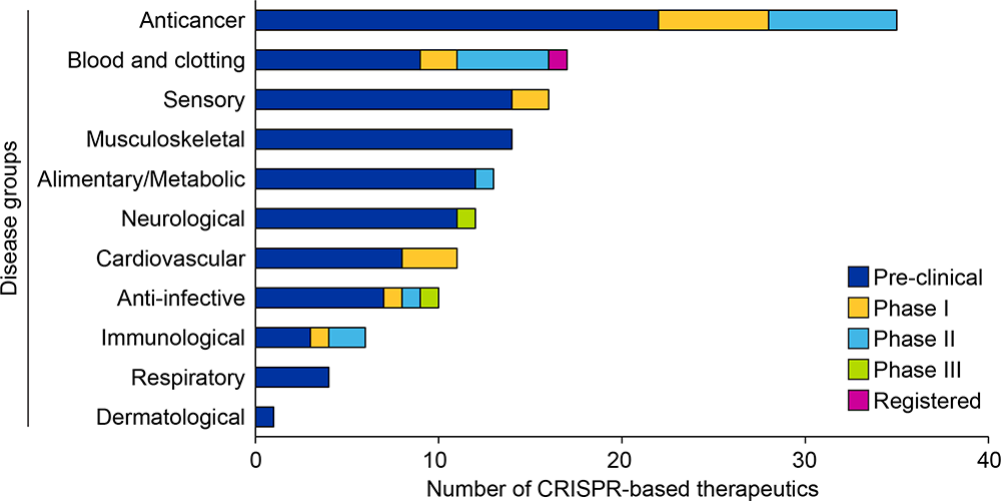
Distribution of CRISPR-based therapeutics currently under development among different disease groups. The stacked bar shows the split of therapeutics among various stages of development for each disease group.

CRISPR-based therapeutics in preclinical and clinical trials focused on the treatment of neurological conditions (Figure S11) including amyotrophic lateral sclerosis (ALS), anxiety, depression, and Alzheimer’s disease. Of these, 92% are in the preclinical research stage and 8% in clinical trial phases (Figure 12 and Figure S11). Of the 9% of CRISPR-based therapeutics aimed at the treatment of alimentary or metabolic diseases, 92% are in the preclinical stage and include diseases such as hyperoxaluria, hepatic dysfunction, inflammatory bowel disease, type 1 diabetes, Pompe’s disease,^222,223^ radio/chemotherapy-induced GI injury, and ulcerative colitis, and at present, only one CRISPR-based therapy named CTX-211 has reached the phase II clinical trials for the treatment of type 1 diabetes (NCT05565248).

**Figure 12:**
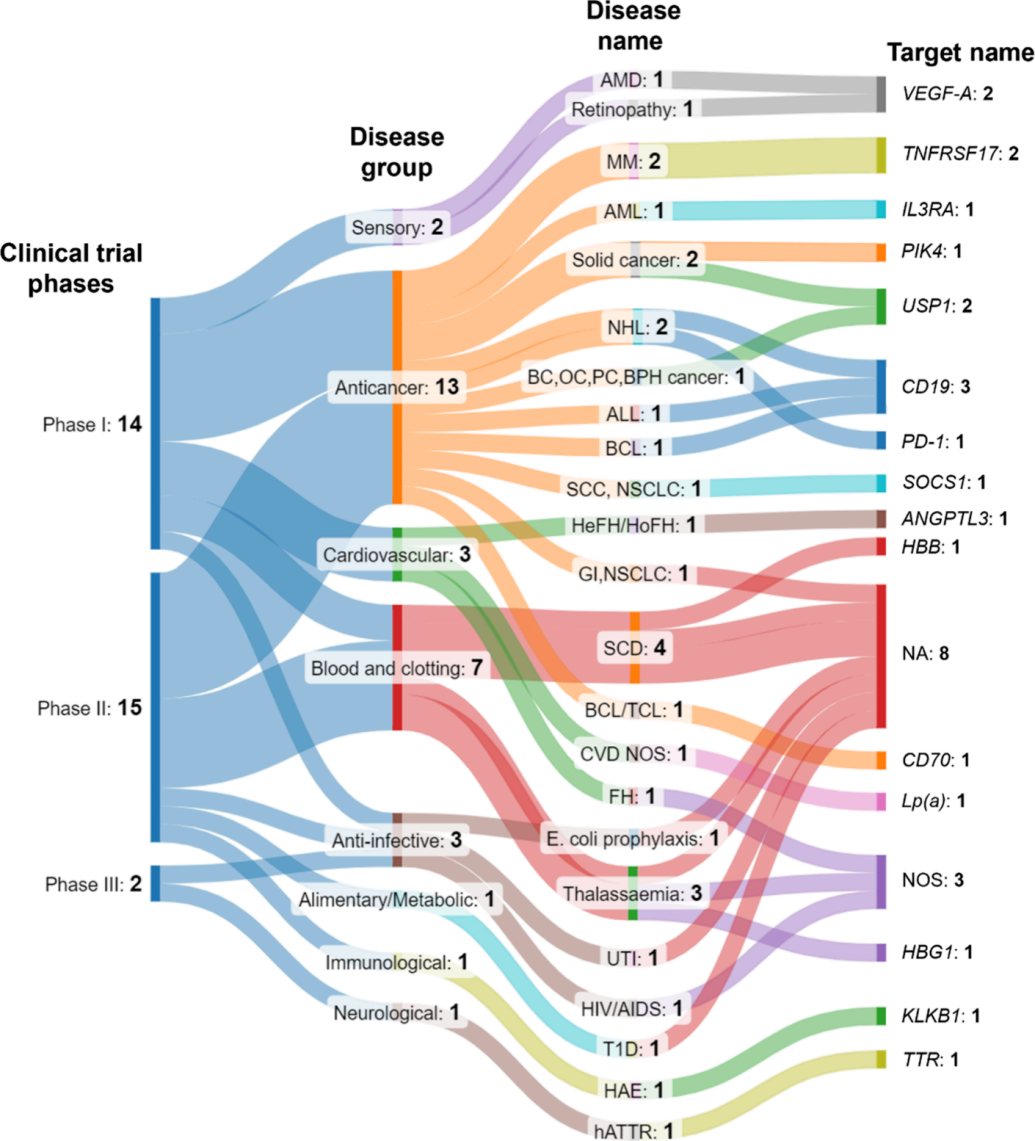
Distribution of CRISPR-based therapeutics in the clinical stages (phase I, II, and III; first column from the left) of development across broader disease groups (second column from the left), individual diseases (third column from the left), and their biological targets (fourth column from the left). Data retrieved from Pharmaproject Citeline Clinical Intelligence in June 2024. The names of the diseases and their targets are abbreviated here as ALL, acute lymphocytic leukemia; AMD, age-related macular degeneration; AML, acute myeloid leukemia; ANGPTL3, angiopoietin-like protein 3; BC, breast cancer; BCL, B-cell lymphoma; BPH, benign prostatic hyperplasia; CD19, cluster of differentiation 19; CD70, cluster of differentiation 70; CVD, cardiovascular disease; FH, familial hypercholesterolemia; HAE, hereditary angioedema; hATTR, hereditary transthyretin amyloidosis; HBB, hemoglobin subunit beta; HBG1, hemoglobin subunit gamma 1; HIV/AIDS, human immunodeficiency virus/acquired immunodeficiency syndrome; IL3RA, interleukin 3 receptor alpha; KLKB1, kallikrein B1; Lp­(a), lipoprotein (a); MM, multiple myeloma; NA, not applicable; NHL, non-Hodgkin’s lymphoma; NSCLC, nonsmall cell lung cancer; NOS, not specified; OC, ovarian cancer; PC, pancreatic cancer; PD-1, programmed death 1; Plk4, polo-like kinase 4; SCC, squamous cell carcinoma; SCD, sickle cell disease; SOCS1, suppressor of cytokine signaling 1; TCL, T cell lymphoma; T1D, type 1 diabetes; TNFRSF17, TNF receptor superfamily member 17; TTR, transthyretin; USP1, ubiquitination-specific proteases; UTI, urinary tract infection; VEGF-A, vascular endothelial growth factor A.

Several different CRISPR-based therapies tackle infectious diseases (7%) (Figure 11), from CRISPR-enhanced bacteriophages to the excision of integrated retroviruses, and even epigenetic silencing of entire viral genomes. LBPEC-01, an anti-infective CRISPR-based therapy in phase III clinical trial (NCT05488340), is a bacteriophage, under development by Locus Biosciences, using CRISPR/Cas3 (crPhage) technology for the treatment of urinary tract infections caused by *Escherichia coli* and *Klebsiella pneumoniae*.^224^ The first-ever CRISPR therapy for HIV, EBT-101, aims to cut the virus from the genome of human cells using CRISPR/Cas9 and two gRNAs, delivered via AAV9 (NCT05144386). Data presented at the 27th American Society of Gene & Cell Therapy (ASGCT) meeting revealed that EBT-101 met the primary and secondary end points of safety and biodistribution/immunogenicity, respectively. However, EBT-101 did not prevent viral rebound in three individuals who stopped antiretroviral medication in a phase 1/2 clinical trial.^225^

The Sankey charts in Figures 12 and Figure S11 depict the breakdown of CRISPR-based therapeutics across phases of development, broader disease groups, individual diseases, and specific gene targets. A few key takeaways from these Sankeys are as follows:

1. A majority of CRISPR-based therapeutics currently in the developmental pipeline are aimed at treating cancers ranging from solid cancers such as nonsmall cell lung cancer (NSCLC) and hepatocellular carcinoma (HCC) as well as hematological malignancies such as AML and multiple myeloma (MM) among others.
2. Many of the targets (35%) currently explored in preclinical stages remain unspecified.
3. Among the specified targets, gene editing via the CRISPR system of dystrophin is being explored to permanently correct *DMD* mutations and thus restore the reading frame, allowing for the production of functional dystrophin and aid in the treatment of muscular dystrophy.^226^
4. Similarly, CRISPR-based strategies are also being investigated for facioscapulohumeral muscular dystrophy (FSHD) and merosin-deficient congenital muscular dystrophy type 1A (MDC1A), which are caused by the aberrant expression of the *DUX4* gene in the muscle tissue^227^ and mutation in the laminin alpha 2-chain (*LAMA2*) gene encoding laminin alpha 2 (Lama2) protein, respectively.^228^

In terms of sheer number of CRISPR-based therapeutics in the developmental pipeline, the leading organization is CRISPR Therapeutics contributing 17% of CRISPR-based therapeutics in preclinical and clinical development. With a focus on the development of transformative medicines using its proprietary CRISPR/Cas9 gene editing platform, CRISPR Therapeutics in collaboration with Vertex Pharmaceuticals launched the first-ever U.S. FDA-approved CRISPR-based therapy Casgevy.^125^ Other key players that are actively involved in developing CRISPR-based therapeutics include Intellia Therapeutics (10%), followed by Arbor Biotechnologies (8%), and Chengdu Gene Vector Biotechnology (6%), among others (Figure S12A). Geographical distribution of companies engaged in CRISPR-based research and development indicates that the U.S. is the leader accounting for 46%, followed by China (14%) and Switzerland (12%) (Figure S12B). While American universities, research institutions, and biotech companies have spearheaded much of the work on CRISPR technology, China has also been a key player (14%) in applying CRISPR technology in clinical settings.^229^ The country has launched a variety of clinical trials, particularly focusing on cancer treatment using CRISPR-edited immune cells.^230^

## CRISPR in Disease Diagnosis

CRISPR technology, originally harnessed for gene editing, has rapidly evolved into a powerful tool for disease diagnosis.^231–233^ Its ability to detect specific genetic sequences is invaluable in identifying infectious diseases, genetic disorders, and even cancers. Although quantitative polymerase chain reaction (qPCR)-based nucleic acid detection is a gold standard method in routine clinical practice,^234,235^ it relies on optimizing numerous processes, such as DNA or RNA extraction, primer design, amplicon detection, and data normalization.^236,237^ Isothermal amplification and next-generation sequencing (NGS) are also used in routine clinical diagnostics. For comparisons between the three most prevalent molecular diagnostic methods please see Table S3 in the Supporting Information.

The CRISPR/Cas system can integrate the ease of use and cost efficiency of isothermal amplification with the diagnostic accuracy of PCR for genotyping and aid in detecting cancer mutations and mutations that confer resistance to antibiotics, antiviral medicines, or cancer drugs. Additionally, the CRISPR/Cas system can fulfill the ASSURED criteria (affordable, sensitive, specific, user-friendly, rapid, equipment-free, delivered) set by the World Health Organization^238^ for infectious disease diagnostics.

The various Cas proteins, combined with other technologies such as biosensors, biochips, biomagnetic beads, isothermal amplification, lateral flow, and protein aptamers, have led to the development of new molecular diagnostic methods with high sensitivity, specificity, low cost, short turnaround time, and portability in complex biological specimens.^293^ Most current CRISPR/Cas-mediated diagnostic assays utilize Class 2 CRISPR/Cas systems that consist of type II (Cas9), type V (Cas12 and Cas14), and type VI (Cas13) CRISPR/Cas systems employing single multidomain effectors. The class 1 type I CRISPR/Cas3 system is also emerging for nucleic acid detection.^294^ The CRISPR/Cas12a, CRISPR/Cas13a, CRISPR/Cas14a, and CRISPR/Cas3 systems depend on the measurement of *trans*-cleavage activity triggered by target sequence recognition,^295,296^ with *trans*-cleavage activity being inhibited or nonspecifically activated by target-independent factors.^297^ The CRISPR/Cas9 system possesses excellent DNA recognition capability but does not possess *trans*-cleavage activity, and has been developed for biosensor-based diagnostics.^301–303^ Only the CRISPR/Cas12a and CRISPR/Cas9 systems are available for dsDNA recognition. In this section, we have discussed the publication landscape of CRISPR-based disease diagnostics and briefly described their mechanisms.

### Publication Landscape on CRISPR-Based Disease Diagnostics

Our data analysis indicates more than 6,600 and 2,900 journal articles and patent publications, respectively, on the application of CRISPR technology in disease diagnosis from 2004 to 2024, which accounts for 17 and 21% of total journal articles and patent publications, respectively, on CRISPR therapeutics in CAS Content collection (Figure 13). Publication trends of CRISPR in disease diagnosis has shown a remarkable increase in recent years, reflecting its growing importance as a diagnostic tool in molecular biology and medical research. The COVID-19 pandemic coincides with accelerated use of CRISPR-based diagnostics with a notable increase in publications (44%) between 2020 and 2022. Patent publications on CRISPR-based disease diagnosis have also surged in recent years, paralleling the technology’s rapid adoption in research and clinical applications.

**Figure 13:**
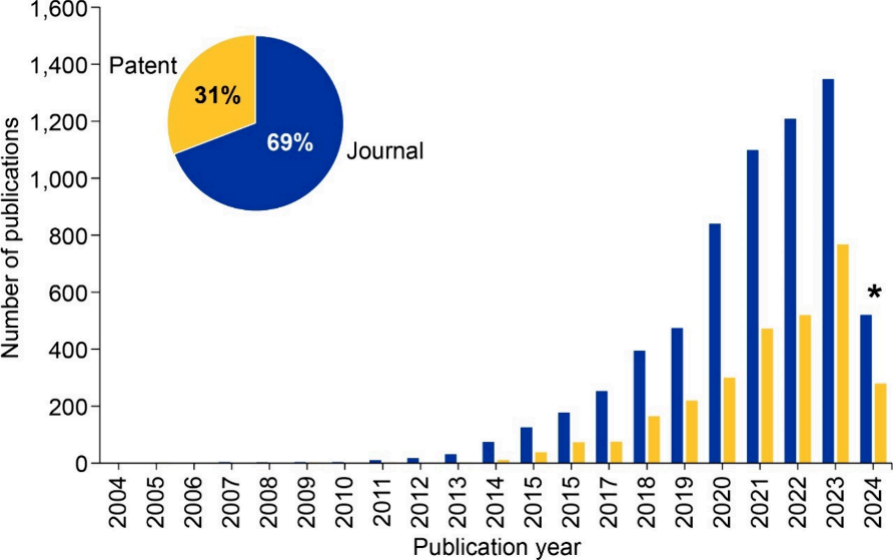
Journal and patent publication trends on CRISPR-based disease diagnostics from the CAS Content Collection for the period 2004 to 2024. *Note that data for 2024 is incomplete due to time of data extraction and encompasses data for January to June.

The publication trends on CRISPR technology and its various Cas proteins associated with diagnosis have evolved significantly over the past decade as the diversity of Cas systems has expanded (Figure 14). Each Cas protein has unique properties and has been adapted for various applications. Cas9 was the first and most widely studied protein in CRISPR research. Early studies predominantly focused on gene editing, but some initial exploration of Cas9’s potential for diagnostics began in 2014 with a steady increase in publications (Figure 14B). The discovery of Cas12 (for DNA detection) and Cas13 (for RNA detection) led to major breakthroughs in diagnostics, especially with the development of the SHERLOCK (Cas13-based) (Sherlock Biosciences) and DETECTR (Cas12-based) (Mammoth Biosciences) platforms. Publications on CRISPR/Cas12 increased several-fold since 2019 indicating development of accurate, fast, and scalable testing solutions. Similarly, publications on Cas12 and Cas13 surged due to their applications in infectious disease detection (e.g., Zika, Dengue, and HPV), multiplexed diagnostics (e.g., influenza, HIV, and SARS-CoV-2), and cancer. Although publications on other Cas proteins such as Cas14 and Cas3 represent a small fraction of the CRISPR diagnostics literature, they highlight emerging areas of interest. Cas14 is notable for its unique ability to detect ssDNA and dsDNA, offering enhanced versatility for developing sensitive and specific diagnostic platforms. Cas3, known for its ability to degrade long stretches of DNA, has been explored in genome editing strategies that may eventually contribute to diagnostic innovations.

**Figure 14:**
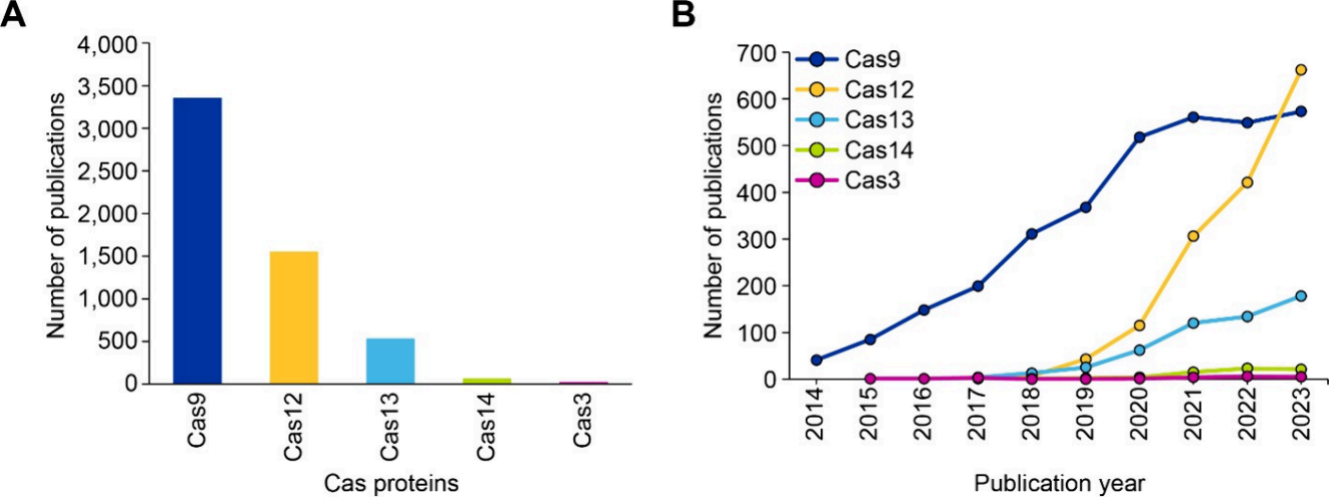
(A) Distribution of publications (journal and patent) based on Cas proteins—Cas9, Cas12, Cas13, Cas14, and Cas3—in publications related to the application of CRISPR in diagnostics. (B) Year-wise distribution of publications (journal and patent) associated with various Cas proteins in the CRISPR diagnostics subset of publications. Data includes journal and patent publications from the CAS Content Collection for the period 2014 to 2023.

The analysis of CRISPR-based disease diagnostics publications co-occurring with various diseases (Figure 15) reflects a growing interest in both infectious and noninfectious diseases. Viral infections in infectious diseases and cancer in noninfectious diseases led the way with the highest number of publications, followed by bacterial, genetic, immune, and fungal diseases (Figure 15A). Publications on CRISPR-based disease diagnostics co-occurring with cancer show continuous and constant growth since 2014, whereas publications on viral diseases show a sudden and steep spike in 2019 followed by plateauing (Figure 15B).

**Figure 15:**
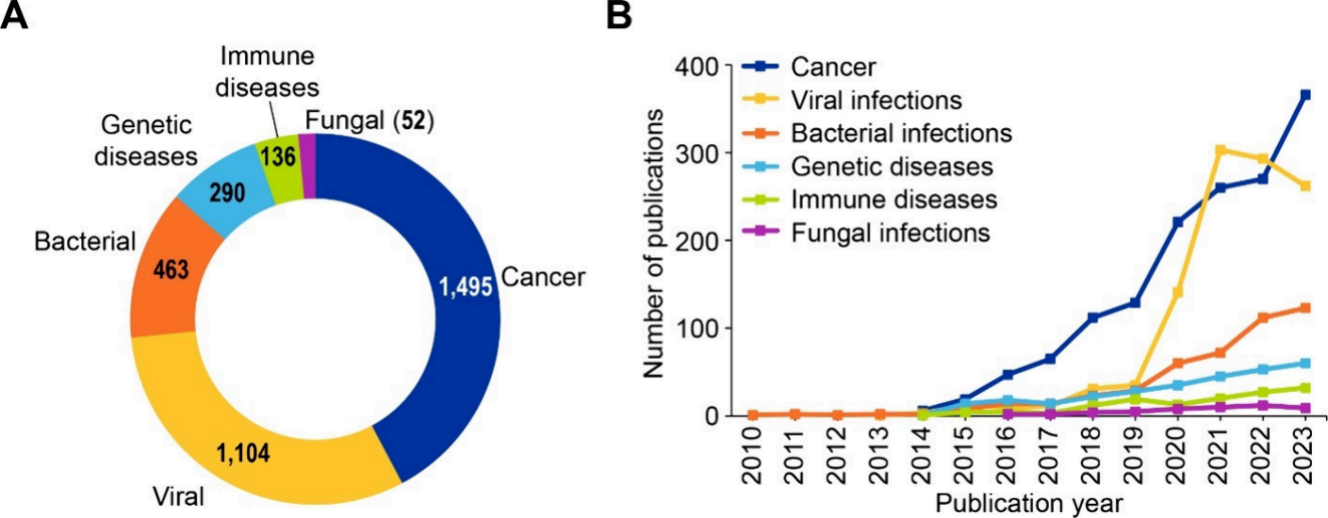
(A) CRISPR-based disease diagnostics documents co-occurring with various diseases (cancer, viral, bacterial, genetic, immune, and fungal diseases. (B) Time trend for CRISPR-based disease diagnostics publications co-occurring with various diseases. Data includes journal and patent publications from the CAS content Collection for the period 2010–2023.

The intersection of CRISPR technologies with preamplification methods for disease diagnosis is a dynamic and rapidly growing area of research, driven by the need for sensitive, specific, and rapid diagnostic tools for various diseases including infectious diseases and cancers. Many diagnostic methods based on CRISPR require preamplification to detect low-abundance nucleic acids. 56% of publications appear to be associated with PCR as a preamplification technique in combination with CRISPR diagnostics to achieve low-cost and point-of-care solutions. This is followed by recombinase polymerase amplification (RPA) (18%), loop-mediated isothermal amplification (LAMP) (7%), etc. Recent publications are also exploring nonamplification methods (4%), focusing on simpler, faster, and more portable diagnostic systems (Figure 16A). Various readout methods are used to interpret the results of CRISPR diagnostics, ranging from simple colorimetric assays to more complex fluorescence-based systems. Fluorescence and sequencing readouts dominate the landscape (39 and 33% respectively), with growing interest in lateral flow, electrochemical, colorimetric, luminescence, and optical (Figure 16B).

**Figure 16:**
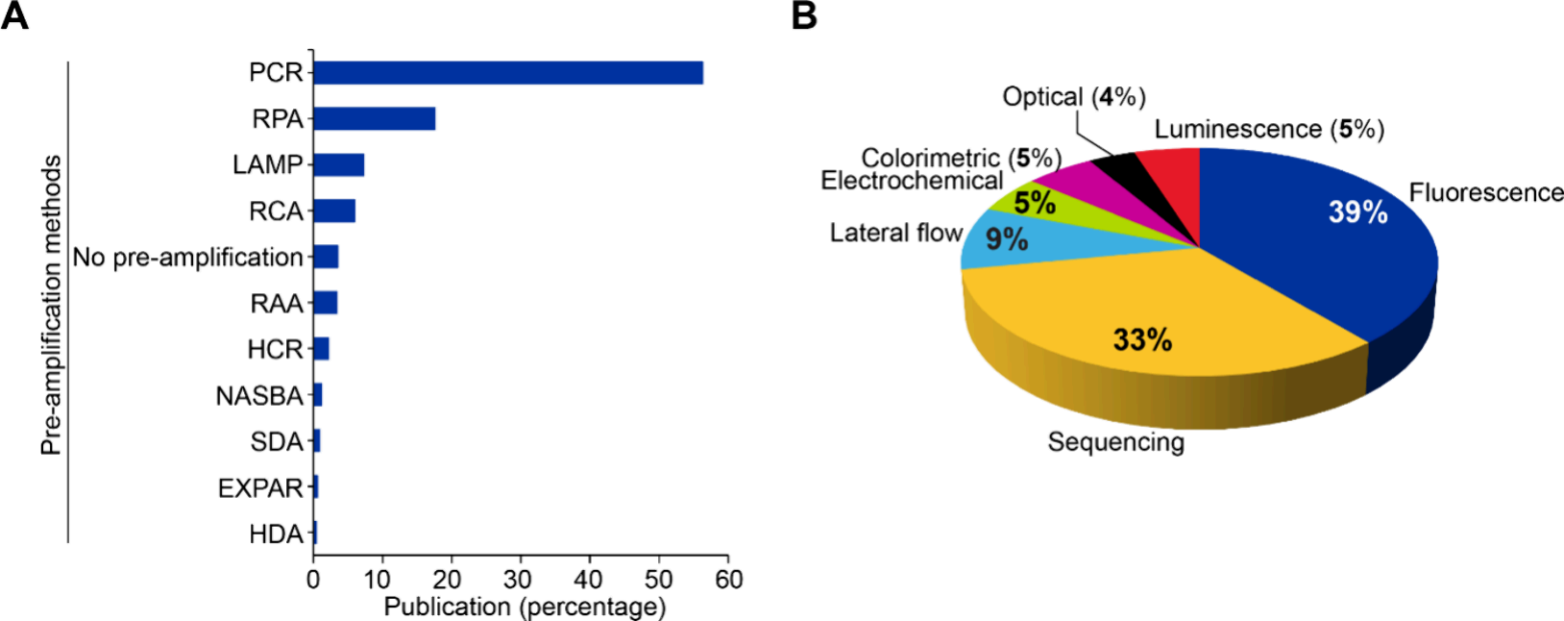
(A) Number of journal and patent publications on various preamplification methods used in CRISPR technology-based nucleic acid detection and diagnosis. (B) Number of journal and patent publications on methods of readout used for CRISPR-based diagnosis. Data includes journal and patent publications from the CAS Content Collection for the period 2004 to 2023. Abbreviations used: EXPAR, exponential amplification reaction; HAD, helicase-dependent amplification; HCR, hybridization chain reaction; LAMP, loop-mediated isothermal amplification; NASBA, nucleic acid sequence-based amplification; PCR, polymerase chain reaction; RAA, recombinase-aided amplification; RCA, rolling circle amplification; RPA, recombinase polymerase amplification; SDA, strand displacement amplification.

### Mechanisms of CRISPR/Cas-Based Diagnostics

CRISPR/Cas-based diagnostics leverage the precise targeting capabilities of the CRISPR/Cas system, particularly variants such as Cas9, Cas12, Cas13, Cas14, and Cas3, to recognize and bind to a target nucleic acid sequence followed by cleavage used to generate a detectable signal. The key mechanisms of various CRISPR/Cas-based platforms developed for disease diagnosis have been described in Table S4, and details of individual detection platforms are summarized in Tables S5–S7 with schematics of various detection platforms depicted in Figures S13–S16.

## CRISPR: Delivery Systems

The ability to target and modify specific genomic sequences holds promise for treating a myriad of genetic disorders, from monogenic diseases to complex, multifactorial conditions. In practice, however, CRISPR-based therapeutics must enter the desired cells without eliciting an unwanted immune response, so a delivery system is required. Thus, despite its transformative potential, the therapeutic application of CRISPR faces significant challenges, particularly in the realm of delivery systems.^239–241^ Effective and safe delivery of CRISPR components—such as the Cas9 nuclease and sgRNA—to target cells and tissues is paramount for achieving desired therapeutic outcomes while minimizing off-target effects and immune responses. The choice of delivery method can significantly influence the efficiency, specificity, and safety of CRISPR-mediated gene editing.

Carriers currently used for delivery of gene editing system cargo fall into three general groups: (i) viral vectors, (ii) nonviral vectors, and (iii) physical delivery (Figure 17).^242–246^ Viral vectors have been extensively studied and utilized due to their high efficiency in delivering genetic material. Among them, AAVs, lentiviruses, and adenoviruses are the most used. AAVs are particularly favored for their low immunogenicity and ability to infect both dividing and nondividing cells, making them suitable for a wide range of tissues. Lentiviruses, derived from HIV-1, can integrate into the host genome, providing long-term expression of the CRISPR components. However, the potential for insertional mutagenesis remains a concern. Adenoviruses offer transient expression and can carry larger genetic payloads, but their high immunogenicity can limit their use in clinical settings. The unfavorable effects of the viral vectors such as genome integration, immunogenetic responses, and limited cargo loading impede further clinical applications.^247,248^

**Figure 17:**
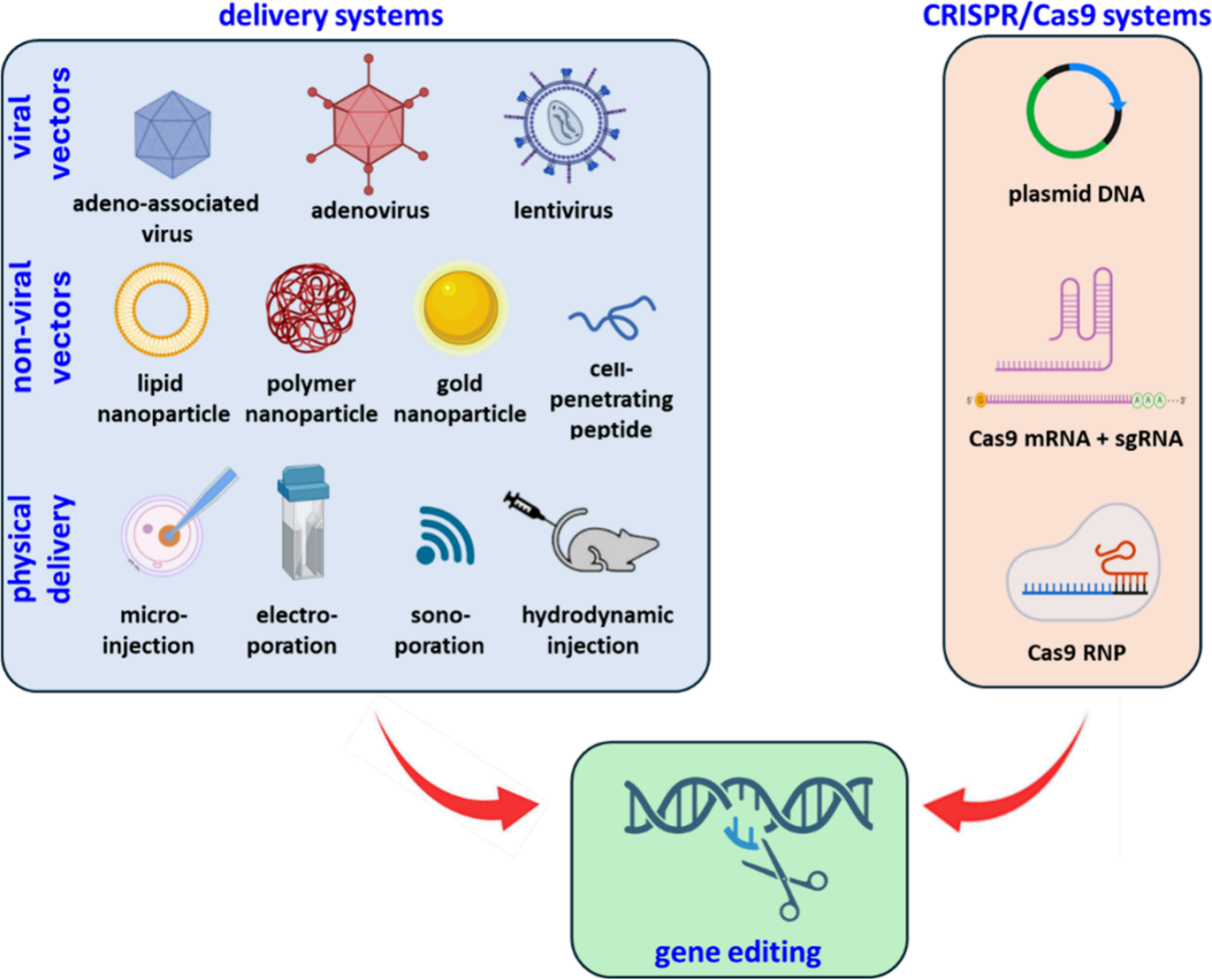
Schematic representation of the various CRISPR/CAS9 delivery systems. Partially created with www.BioRender.com.

Nonviral vectors, including lipid-, polymer-, or metal-based nanocarriers and cell-penetrating peptides (CPPs), offer an alternative approach in CRISPR delivery. Although considered not as prominent as viral-based delivery vectors, they possess the advantages of lower immunogenicity and toxicity, and huge cargo size, and are a proliferating area of research.^242,249,250^

Negatively charged nucleic acids can be electrostatically complexed to cationic materials with the complexes subsequently endocytosed by cells. The most successful classes of cationic materials applied so far for nucleic acid delivery are lipids, e.g., rationally designed lipids and lipid-like materials, and naturally occurring and synthetic polymers. Ideally, any nonviral delivery material for genome editing should be well tolerated—biocompatible, nonimmunogenic, and capable of delivering payloads to the nucleus.^251^

Thus, lipid-based nanoparticles can encapsulate CRISPR components and facilitate their delivery into cells via endocytosis. Polymer-based systems, such as polyethylenimine (PEI) and poly­(lactic-*co*-glycolic acid) (PLGA) nanoparticles, provide customizable platforms for delivering CRISPR payloads with controlled release profiles. Nanoparticles offer unique advantages in terms of size, surface modification, and targeting capabilities. These nanocarriers can enhance cellular uptake and provide protection for CRISPR components from degradation. Exosomes, which are naturally occurring extracellular vesicles, have garnered interest due to their inherent biocompatibility and ability to mediate intercellular communication. Engineering exosomes to deliver CRISPR components holds promise for achieving targeted and efficient gene editing with minimal immunogenicity.^252–254^

In some cases, delivery vectors are not necessary for genome editing. In *ex vivo* therapies, mechanical intervention can create transient holes in cell membranes, allowing nucleic acids and proteins to enter the cell. The most common physical delivery methods include microinjection and electroporation/sonoporation, while methods such as hydrodynamic delivery are currently under development. Optimization of the *in vivo* CRISPR delivery still faces multiple challenges, including encapsulation of large size CRISPR system, targeted delivery, and enhanced endocytosis.^255–257^ In addition to gene editing, CRISPR systems have been developed for delivery of drugs, such as doxorubicin—e.g., CRISPR-dCas9.^258^ Thus, based on the potent functions of the CRISPR system for disease correction, efficient *in vivo* delivery systems are urgently needed.

With regards to CRISPR/Cas9 cargoes, three forms have been explored: (i) **plasmid** DNA encoding both Cas9 protein and the sgRNA; (ii) a mixture of Cas9 **mRNA** and a separate sgRNA; and (iii) a mixture of Cas9 protein and the sgRNA (Cas9 **ribonucleoprotein**, **Cas9 RNP**) (Figure 17).^259–261^ It is now widely believed that the safest delivery method for CRISPR is to deliver it as a complete RNP. By delivering the Cas enzyme and gRNA as a preformed RNP complex, the amount of time the complex spends in the cells is reduced, minimizing the risks of triggering an immune response or off-target editing of the genome.^262^

An outline of the various delivery systems for CRISPR therapeutics is summarized in Table 4.

**Table 4:** Delivery Systems/Vectors for CRISPR Therapeutics

**delivery system/vector**	**advantages**	**disadvantages**	**mechanism**	**applications**	**example**
**Viral vectors**					
adeno-associated virus (AAV)	high transduction efficiency, low immunogenicity, ability to infect nondividing cells	limited packaging capacity (∼5 kb), potential for pre-existing immunity	AAV vectors deliver CRISPR components by infecting target cells, where the viral DNA is expressed	used in gene therapy for treating genetic disorders such as Duchenne muscular dystrophy and hemophilia	Luxturna (voretigene neparvovec): the first FDA-approved gene therapy for treating inherited retinal disease. Uses AAV to deliver a functional copy of the RPE65 gene.^263–265^
lentivirus	ability to integrate into the host genome, large packaging capacity (∼8 kb), stable expression	risk of insertional mutagenesis, potential for long-term effects	lentiviral vectors integrate CRISPR components into the host genome, ensuring stable expression	suitable for long-term gene therapy applications, such as treating HIV or genetic blood disorders	CAR-T cell therapy: lentiviral vectors are used to modify T cells to express CARs for cancer immunotherapy.^266,267^
adenovirus	high transduction efficiency, large packaging capacity (∼36 kb)	high immunogenicity, transient expression	adenovirus vectors deliver CRISPR components as episomal DNA, resulting in transient expression	used for transient gene editing applications and cancer gene therapy	clinical trials for muscular dystrophy: adenovirus vectors are being investigated for delivering CRISPR components to correct mutations in the dystrophin (*DMD*) gene in muscle cells.^268–271^
**Nonviral vectors**					
lipid nanoparticles (LNPs)	low immunogenicity, ability to carry large cargoes, adaptable for mRNA delivery	potential for off-target effects, need for optimization of lipid composition	LNPs encapsulate CRISPR components, facilitating cellular uptake and endosomal escape	widely used for mRNA-based CRISPR delivery, such as in liver-targeted therapies	Moderna’s mRNA vaccine platform: while primarily used for mRNA vaccines, LNPs are also being explored for delivering CRISPR components for gene editing applications.^272–274^
polymeric nanoparticles	versatility in design, ability to encapsulate various types of nucleic acids, biodegradable	potential for toxicity, need for extensive optimization	polymeric nanoparticles encapsulate CRISPR components and release them in a controlled manner	used for sustained release applications and targeting specific tissues	cationic polymers for gene editing: researchers are developing polymer NPs based on cationic polymers like PEI to deliver CRISPR/Cas9 plasmids for cancer therapy.^275–277^
gold nanoparticles	high stability, ease of functionalization, low toxicity	limited cargo capacity, need for complex surface modification	gold nanoparticles are functionalized with CRISPR components for cellular uptake	used in precision medicine for targeted gene editing in cancer cells	targeted cancer therapy: AuNPs conjugated with CRISPR components are being investigated for targeted gene editing in cancer cells to knock out oncogenes.^244,278–280^
cell-penetrating peptides (CPPs)	ability to deliver cargo directly into the cytoplasm, minimal toxicity	limited cargo size, potential for off-target delivery	CPPs facilitate the direct delivery of CRISPR components into the cytoplasm	used for intracellular delivery of nucleic acids and proteins	Tat peptide for protein delivery: the HIV-1 Tat peptide is used to deliver Cas9 protein and sgRNA into cells for efficient gene editing *in vitro*.^281–283^
**Physical methods**					
electroporation	high efficiency, ability to transfect a variety of cell types	potential for cell damage, limited *in vivo* applicability	electrical pulses create pores in the cell membrane, allowing CRISPR components to enter	used for *ex vivo* gene editing in cell therapy applications	CRISPR-edited T cells for cancer immunotherapy: electroporation is used to introduce CRISPR components into T cells *ex vivo* to knock out PD-1, enhancing their antitumor activity.^284–286^
microinjection	high precision, direct delivery into the nucleus or cytoplasm	labor-intensive, not suitable for high-throughput applications	direct injection of CRISPR components into cells using a fine needle	used in research for precise gene editing in embryos and zygotes	gene editing in mouse embryos: microinjection of CRISPR/Cas9 components into mouse zygotes is used to create genetically modified mice for research.^287–289^
hydrodynamic injection	simple technique, effective for delivering plasmids to the liver	limited to certain tissues, potential for tissue damage	rapid injection of a large volume of CRISPR components into the bloodstream, creating transient pores in endothelial cells	used primarily for liver-targeted gene therapy	liver-specific gene editing: hydrodynamic injection of CRISPR plasmids into mice for liver-specific gene editing to study metabolic diseases.^290,291^
gene gun (Biolistics)	can penetrate cell walls, effective for plant cells. Allows for direct delivery to tissues.	potential tissue damage. Variable efficiency and low cell viability. Limited to accessible tissues.	the gene gun propels microscopic gold or tungsten particles coated with CRISPR components into target cells using a high-velocity helium pulse.	primarily used for plant cells, but also applicable to certain animal tissues and cells. Useful *for in vivo* applications where other methods are less effective.	plant genetic engineering: Biolistic delivery of CRISPR/Cas9 plasmids into plant cells to generate genetically modified crops with desired traits.^292–294^
ultrasound (sonoporation)	noninvasive and can be targeted to specific tissues. Enhances membrane permeability.	requires optimization to avoid tissue damage. Variable efficiency. Limited to certain tissues.	ultrasound waves create cavitation bubbles that disrupt cell membranes, allowing CRISPR components to enter the cells.	used for both *in vitro* and *in vivo* gene editing. Potential applications include targeted delivery to tumors and other tissues.	targeted cancer gene therapy: sonoporation is used to enhance the delivery of CRISPR components to tumor cells in animal models for gene knockdown studies.^295–297^
laser-induced poration	high precision and control. Minimal invasiveness.	requires specialized equipment. Potential thermal damage to cells and tissues. Limited throughput.	lasers create transient pores in the cell membrane, facilitating the entry of CRISPR components.	used for precise delivery to specific cells or tissues in research settings. Potential for applications in dermatology and ophthalmology.	dermatology applications: laser-induced poration is used to deliver CRISPR/Cas9 components into skin cells for potential treatments of skin disorders.^298–300^

### Viral Vectors

1. **Adeno-associated viruses (AAVs)** are small viruses that infect humans and some other primate species. They are not known to cause disease and have a low immune response, making them suitable for gene therapy. AAVs can deliver genes by infecting cells and inserting the therapeutic gene into the cell’s DNA. The limited cargo size is a significant challenge, often necessitating the use of smaller Cas9 variants or split Cas9 systems. Nonpathogenic, low immunogenicity, limited cargo capacity (∼5 kb), stable expression in nondividing cells.
2. **Lentiviruses** are a type of retrovirus that can integrate their genetic material into the host cell genome, enabling long-term expression. They can infect both dividing and nondividing cells and have a larger cargo capacity than AAVs, accommodating full-size Cas9. However, their integration into the host genome raises concerns about insertional mutagenesis and oncogenesis. High transduction efficiency, larger cargo capacity (∼8 kb), long-term expression, potential safety risks due to genome integration.
3. **Adenoviruses** are common viruses that cause mild infections in humans. They can deliver large DNA sequences and do not integrate into the host genome, which reduces the risk of insertional mutagenesis. However, they can elicit strong immune responses, which can be problematic for repeated treatments. Large cargo capacity (∼8–10 kb), high efficiency, transient expression, potential for strong immune responses.

### Nonviral Vectors

1. **Lipid nanoparticles (LNPs)** are tiny vesicles composed of lipids that can encapsulate nucleic acids, such as mRNA or small interfering RNA (siRNA), protecting them from degradation and facilitating cellular uptake. LNPs are widely used for delivering RNA-based CRISPR components and have been proven effective in recent mRNA vaccines. They protect RNA, facilitates uptake, low immunogenicity, and potential toxicity at high doses.
2. **Polymeric nanoparticles** are made from biodegradable polymers and can carry DNA, RNA, or protein cargoes. They can be engineered to release their payloads in a controlled manner, targeting specific cells or tissues. Their versatility allows for customization in design and functionality enabling carrying of various cargo types.
3. **Cell-penetrating peptides (CPPs)** are short peptides that facilitate the delivery of various molecules, including nucleic acids and proteins, across cell membranes. They are versatile and can be conjugated with different cargoes, though their efficiency can vary. They can deliver a variety of cargoes, minimal toxicity, and variable efficiency.
4. **Gold nanoparticles** can be functionalized with nucleic acids and are used for their stability and ease of modification. They can deliver CRISPR components into cells effectively but are expensive and may be toxic at high concentrations. They are biocompatible, are easily functionalized, have effective delivery, and have high cost.

### Physical Methods

1. **Electroporation** involves applying an electric field to cells to create temporary pores in their membranes, allowing CRISPR components to enter. This method is highly efficient but can cause significant cell damage and is less suitable for *in vivo* applications. It has high efficiency, is applicable to various cell types, and has potential cell damage.
2. **Microinjection** involves directly injecting CRISPR components into individual cells using a fine needle. This method is precise and commonly used for creating genetically modified embryos but is labor-intensive and not scalable. It is highly precise, suitable for single-cell applications, and labor-intensive.
3. **Hydrodynamic injection** involves rapidly injecting a large volume of solution into the bloodstream, usually targeting the liver. This creates transient pores in cell membranes, allowing CRISPR components to enter. It is mainly used in animal models. It is simple, is efficient for the liver, and has potential tissue damage.
4. **Particle bombardment (gene gun)** uses high-velocity particles (gold or tungsten) coated with CRISPR components to deliver them into target cells. When the particles penetrate the cell membrane, they deliver the CRISPR cargo directly into the cytoplasm. This is effective for plant cells and has some applications in mammalian tissues. It is good for hard-to-transfect cells, has potential cell damage, and lacks precision.
5. **Sonoporation** involves ultrasound waves creating temporary pores in the cell membrane, facilitating the uptake of CRISPR components. It has been used experimentally in tissues like muscle and tumor tissues. It has also shown promise in delivering therapeutics across the blood–brain barrier. It is noninvasive and limited to tissues accessible by ultrasound.

Figure 18 shows the distribution of the documents related to the various types of CRISPR delivery systems in the CAS Content Collection. The largest fraction of publications concern viral vectors, with AAVs being most represented. From the physical delivery methods, electroporation and microinjection appear to be more represented than the other physical methods.

**Figure 18:**
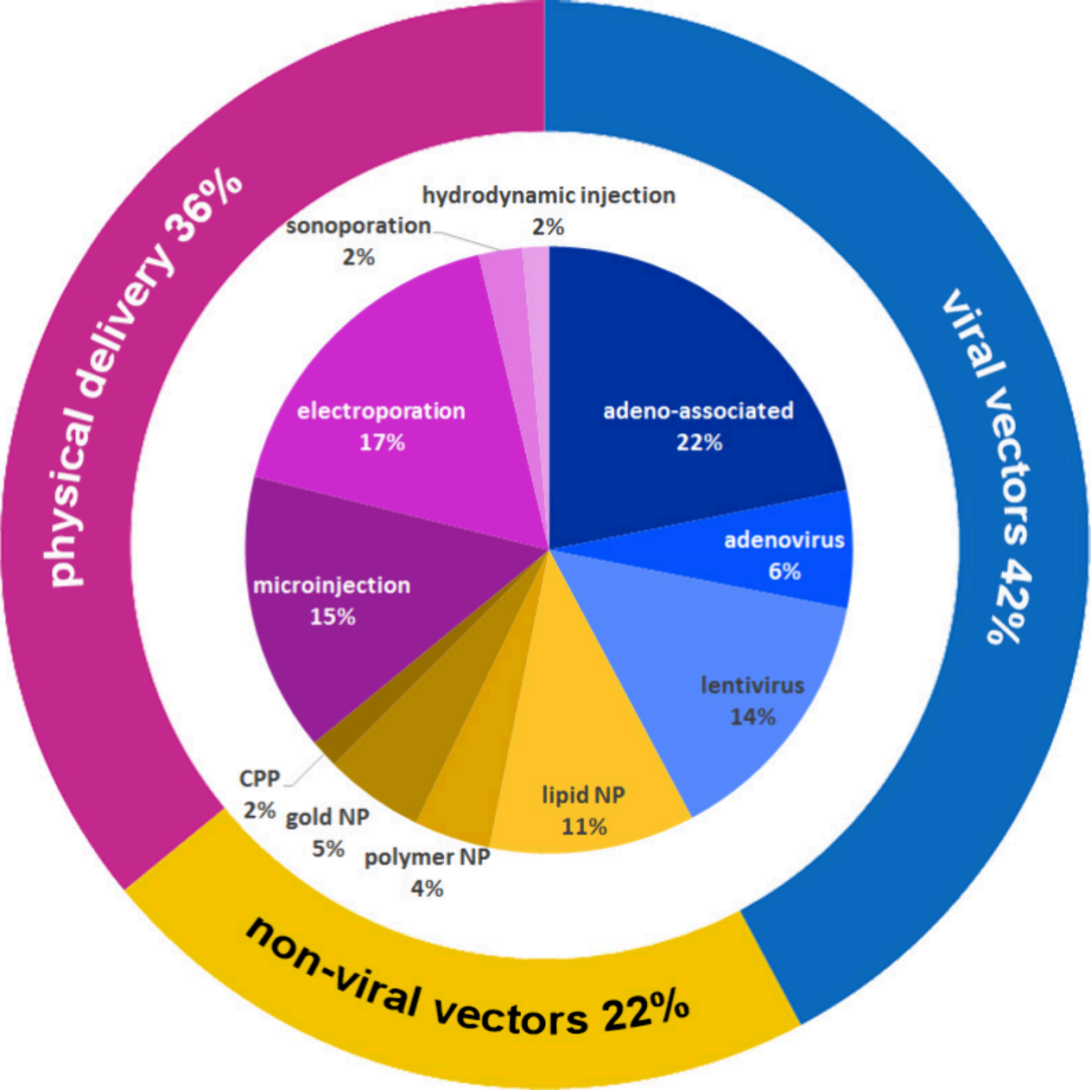
Distribution of the documents related to the various types of CRISPR delivery systems in the CAS Content Collection. Data includes journal and patent publications from the CAS Content Collection for the period 1995–2024.

Figure 19 represents a heatmap showing the relative co-occurrences of diseases targeted by CRISPR and the delivery vectors utilized, with a few takeaways highlighted below:

1. In general, viral vectors (AAV, lentivirus, and adenovirus) and some nonviral vectors (LNPs and polymer nanoparticles) have been explored more than other methods of delivery.
2. Among the physical methods of delivery, electroporation co-occurs to a higher extent as compared to all other methods for most diseases except for liver diseases.
3. Some of the highest correlations are between ocular diseases and AAV, cancer and lentiviral vectors, and liver and cardiovascular diseases and lipid nanoparticles.

**Figure 19:**
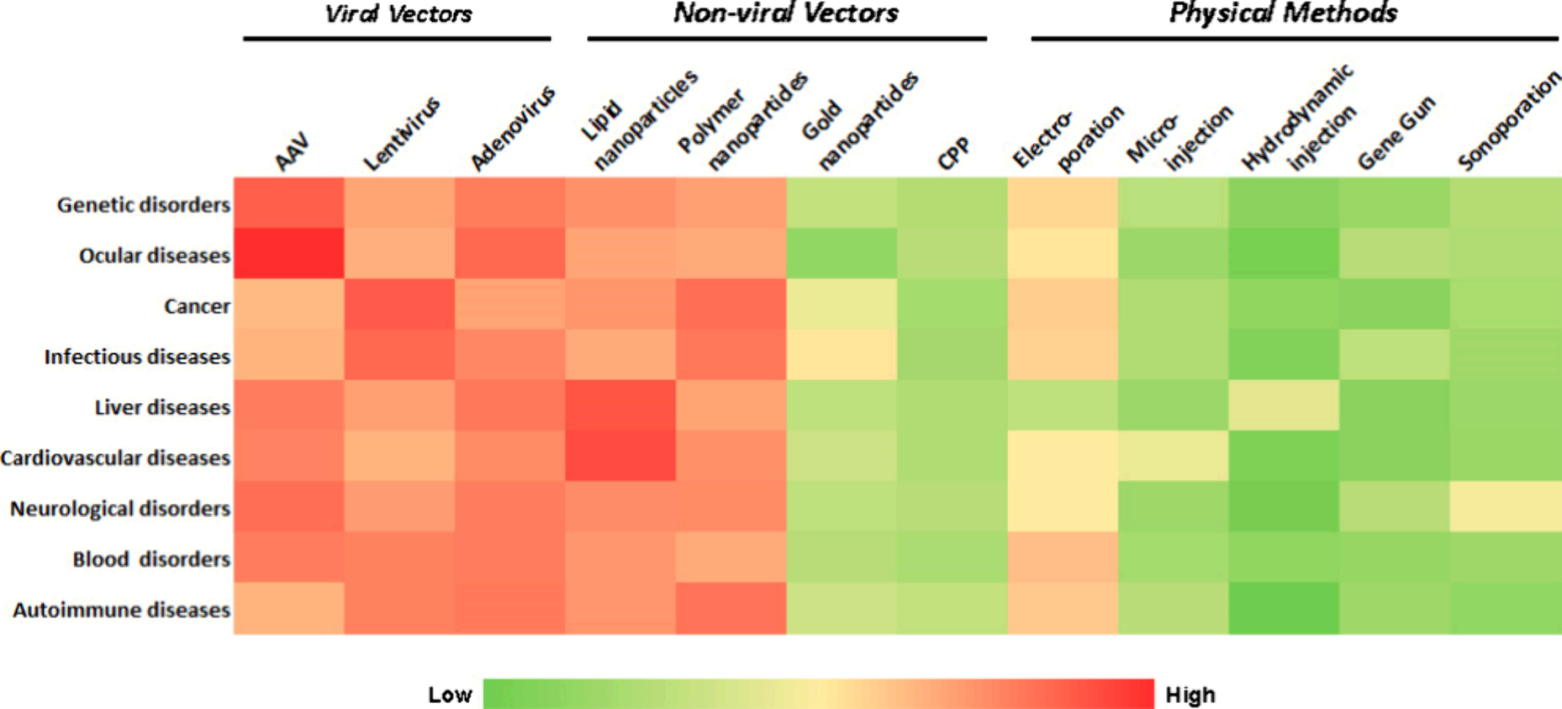
Heatmap showing relative co-occurrences of diseases targeted by CRISPR and the delivery vectors. Listed here are diseases included within each of the broader categories: genetic disorder—sickle cell disease, β-thalassemia, and cystic fibrosis, Duchenne muscular dystrophy, and Tay-Sachs disease; ocular diseases—Leber congenital amaurosis and retinitis pigmentosa; infectious diseases—HIV/AIDS and hepatitis B; liver diseases—antitrypsin deficiency and hereditary tyrosinemia; cardiovascular diseases—familial hypercholesterolemia and hypertrophic cardiomyopathy; neurological disorders—fragile X syndrome, autism spectrum disorders, amyotrophic lateral sclerosis, Huntington’s disease, and Alzheimer’s disease; blood disorders—sickle cell disease and β-thalassemia. Data includes journal and patent publications over the period 1995–2024 from the CAS Content Collection (AVV, adeno-associated virus; CPP, cell-penetrating peptide).

## Ethics

Doudna, one of the inventors of the CRISPR technology, expressed in the 2016 American Association for the Advancement of Science Annual Meeting that one of her biggest fears is “waking up one morning and reading about the first CRISPR baby, and having that create a public backlash where people ban or regulators shut this down, and I think that could be very detrimental to the progress of the field”.^301^ In 2018, her fears were realized when Chinese researcher He Jiankui claimed that he used CRISPR to alter the DNA of seven embryos of couples where the males were HIV carriers to immunize the babies against the HIV virus. This resulted in the birth of two twin girls, the first CRISPR babies.^302,303^

Beauchamp and Childress proposed four main principles of biomedical ethics: beneficence, nonmaleficence, respect for autonomy, and justice.^304^ In summary: proposed “treatment” should result in a positive outcome/benefit (beneficence), avoid or minimize harm as much as possible (nonmaleficence), patients should not be treated without informed consent (autonomy), and equitable access to treatment (justice). When looking at applications and study of CRISPR/Cas genome editing, researchers should take these principles into consideration.^305^ For example, under beneficence and nonmaleficence is the risk of unwanted effects such as genomic off-target activity, immune response, age-related or disease-related challenges that should be considered,^306,307^ and natural genetic diversity that could alter on-target and off-target outcomes.^308,309^ Under justice, an argument is the equitable distribution and accessibility of these expensive, but potentially lifesaving therapies.^310^ In the case of autonomy, there is the argument of embryonic and gamete targeting vs somatic cell targeting. There is less ethical argument when it comes to targeting somatic cells, but the possible human beings that result from any embryonic/gamete genetic modification would lack informed consent as the decision to be modified was not made by them yet would have to live with the consequences of the modification throughout their life.^311,312^

Other ethical concerns are legal regulations, the use of the technology at home by communities without medical supervision (biohackers),^305^ and the use of CRISPR for nontherapeutic purposes like enhancements, eugenics, and even gene terrorists. A survey of laws, regulations, and governance principles on genome editing in humans was also published by the Scientific Foresight Unit of the European Parliamentary Research Service in 2022.^313^ For more information and outlook on the ethical issues regarding the application of CRISPR technologies, we suggest publications by Gonzalez-Avila et al.,^305^ Lorenzo et al.,^311^ Brokowski and Adli,^314^ and Nada Kubikova et al.^315^ as well as news articles and interviews published by NPR,^316^ MIT Technology Reviews,^317^ and the Harvard Gazette.^318^

### Challenges

Despite the wide acceptance of CRISPR technology in gene editing owing to its versatility and ease of use, there remain certain challenges associated with it.

#### Off-Target Effects

In natural setting, CRISPR/Cas systems tolerate mismatches between the gRNA and the target to a certain extent. This is a likely evolutionary consequence to overcome the high mutational rate of phages. However, this property is unsought for genome engineering applications, as it may result in the targeting and editing of off-target sites. Numerous studies have reported off-target activity at sites ranging from a single base mismatch to sites containing multiple consecutive mismatches, or even nucleotide insertions or deletions.^319–322^ Regardless of the mismatch tolerance of CRISPR/Cas9, most potential off-target sites do not result in dsDNA cleavage and gene editing. This might be due to existing intrinsic checkpoints in the DNA binding and cleavage mechanisms of Cas9.^43,323,324^ Notably, high-throughput profiling studies exploring off-target effects have shown that their frequency is consistently lower *in vivo* as compared with isolated genomic DNA.^325,326^

#### PAM Requirement

Another limitation of the technology is the requirement for a PAM near the target site, which restricts its targeting scope. SpCas9 is one of the most extensively used Cas9s with a relatively short PAM recognition site −5′NGG3′ (N is any nucleotide). Theoretically, SpCas9 permits finding a suitable target site every eight nucleotides on an average throughout the genome. However, some genomic regions are not easily targetable by SpCas9 due to a high A/T content. Several naturally occurring orthologs of Cas9 with alternative PAM specificities have been identified and adopted for gene editing; however, many of these have even more limiting PAM requirements.^327–329^

#### Packaging and Delivery

*In vivo* delivery of CRISPR/Cas9 into mammalian cells is generally accomplished using viral vectors. AAVs remain the preferred choice due to their low immunogenicity and high transduction efficiency. However, AAVs have limited packaging capacity and, hence, it is difficult to package the genes encoding most used Cas9 (SpCas9) and its associated sgRNA into a single AAV vector unless compact promoters are used.^330,331^ Another limiting factor for most gene editing components is their safe, efficient, and targeted delivery to the specific organ or tissue. If CRISPR/Cas9 components are delivered *in vivo* via the systemic approach, they can get degraded by circulating proteases or nucleases or get cleared by the mononuclear phagocyte system. Furthermore, other factors such as vascular permeability, diverse endocytosis mechanisms, and lysosomal degradation can result in variable efficacy, which may eventually result in suboptimal therapeutic outcomes.^332^

#### DNA Damage Toxicity

CRISPR-based gene editing relies on introduction of DSBs, which can trigger apoptosis and growth inhibition rather than the intended gene edit.^333^ Additionally, large deletions spanning few kilobases/megabases and complex genetic rearrangements have been reported in several studies highlighting a major biosafety issue for clinical applications of CRISPR therapy.^334,335^ Furthermore, multiple simultaneous off-target edits can ultimately result in genomic rearrangements such as inversions, deletions, and chromosomal translocations and trigger DNA damage and stress response pathways.^334,336,337^

#### Immunotoxicity

Immunogenic toxicity is a known limitation of any gene editing technology, including CRISPR. Pre-existing antibodies against Cas9 and reactive T cells have been identified in humans, and Cas9 immunity has been associated with compromised therapeutic outcomes in various disease models.^338–341^

#### Regulatory Hurdles

Different countries have varying regulations regarding CRISPR-based gene editing, and in some countries, the guidelines are still under development. Also, in most countries, one regulatory agency oversees gene therapy while other agencies regulate genetically modified organisms, and this creates a complex regulatory process for CRISPR-based therapeutics. Additionally, the long-term effects and safety of these therapeutics are not yet fully understood. All of these factors may contribute to lengthy and complex approvals of CRISPR-based therapeutics.

## Conclusions and Looking Ahead

Since the first use of CRISPR-based gene editing, the field has evolved at an exceptional pace exhibiting an average growth in publications of 54% in the past decade (2014–2023). This sustained and extensive interest has resulted in a plethora of publications exploring the use of CRISPR in treating hard-to-cure diseases, disease diagnostics, and identification of genes underlying various disorders.

A majority of leading commercial entities active in the CRISPR space originate in the United States, while patents filed by academic research institutions appears to slightly more evenly divided between organizations in China and United States. Among the various gene targets occurring in the CRISPR data set, *TP53* emerges as the clear leader, growing drastically after 2018. Perhaps unsurprising since mutations in *TP53* have been linked to various types of cancer. These mutations tend to be missense mutations and present great opportunities for the use of CRISPR/Cas technology in correcting/rectifying them. Other notable gene targets appearing frequently include *c-myc*, *HBB*, *KRAS*, and *BRCA1*.

A considerable number of CRISPR-related publications appear to be connected to cancer and infectious diseases, while other diseases such as blood, genetic, and nervous system disorders are also explored in the context of CRISPR/Cas technology. Within the broader category of cancer, breast cancer, AML, liver cancer, lung cancer, and rectal cancer exhibit a remarkable increase in journal publications in the CRISPR data set indicating exploration of this technology in the treatment of or to establish critical genetic targets for these cancer types. Among nervous system disorders, the neurodegenerative diseases Alzheimer’s and Parkinson’s show a marked increase in publications, especially patents, related to CRISPR indicative of greater commercial interest.

The use of CRISPR/Cas technology in disease diagnostics has also seen a surge, most notably after 2019. Cas9 remains the Cas protein of choice in CRISPR/Cas-based diagnostics with the most number of publications associated with it, though in recent years, Cas12 appears to be catching up, managing to exceed Cas9 in 2023. CRISPR/Cas-based diagnostics have found application in detecting pathogens such as Zika virus and MRSA as well as cancer markers.

All of the research and development in the field has translated into considerable increase in commercial interest in CRISPR-based diagnostics and therapeutics over the past few years. Currently, there are >140 CRISPR-based therapeutics in various stages of clinical trials, a quarter of which appear to be for a range of cancer subtypes. Despite the great strides that have occurred in this field, there remain quite a few challenges in using CRISPR/Cas technology for therapeutic purposes. Researchers are actively engaged in developing alternative and better approaches to overcome these limitations. Off-target effects of CRISPR/Cas technology are being addressed by the development of chemically modified gRNAs, high-fidelity nuclease variants, and controlled expression of genome editor nucleases. The PAM sequence requirement of SpCas9 restricts the scope of targetable genomic sites; however, this issue can be addressed using engineered variants of Cas9 with alternative or relaxed PAM requirements or other naturally derived Cas9 orthologs, and Cas12a enzymes. Second-generation CRISPR-based technologies such as base editing or prime editing enable the introduction of precise modifications independently of DSBs. Newer packaging and delivery methods like electroporation/nucleofection and lipid nanoparticles have great potential to overcome existing targeted delivery problems.^342^

The ongoing refinement of existing CRISPR components continue to improve the efficiency and specificity of CRISPR-based therapeutics. Expanding the targeting capabilities and optimizing delivery systems continue to aid in significant improvements in clinical outcomes. Ultimately, in the future CRISPR-based therapeutics are likely to be developed successfully for myriads of diseases beyond cancer.

## Supplementary Material



## Notes

Brief description of methods including data scope and analysis, discussion about CRISPR/Cas biology and mechanism, and types of CRISPR/Cas systems; patent activity, commercial interest in CRISPR, CRISPR in agriculture, and AI in CRISPR; (Tables S1–S7) types of CRISPR/Cas systems, CRISPR/Cas therapeutics currently in the developmental pipeline, CRISPR/Cas9-mediated detection platforms, and other information; (Figures S1 and S2) CRISPR/Cas mechanism and types of CRISPR/Cas system; (Figure S3) patent activity; (Figure S4) commercial activity in CRISPR; (Figure S5) publication trends for AI in CRISPR; (Figures S6–S12) other data analysis related results; and (Figures S13–S16) schematic representations of various CRISPR diagnostic platforms (PDF)

## Abbreviations

4-OHT: 4-hydroxytamoxifen
AaCas12b: *Alicyclobacillus acidiphilus* Cas12b
AAVs: adeno-associated viruses
ABE: adenine base editor
AD: Alzheimer’s disease
AI: artificial intelligence
ALL: acute lymphocytic leukemia
AMD: age-related macular degeneration
AML: acute myeloid leukemia
ANGPTL3: angiopoietin-like protein 3
APP: amyloid precursor protein
ASGCT: American Society of Gene & Cell Therapy
aTFs: allosteric transcription factors
Aβ: amyloid β
B2M: beta-2 microglobulin
BC: breast cancer
BCL: B-cell lymphoma
BHB: β-hydroxybutyrate
BPH: benign prostatic hyperplasia
CARMEN: combinatorial arrayed reactions for multiplexed evaluation of nucleic acids
CAR-NK: chimeric antigen receptor-natural killer
CARP: CRISPR-associated reverse PCR
CAR-T: chimeric antigen receptor-T
Cas: CRISPR-associated proteins
Cas9 RNP: Cas9 ribonucleoprotein
Cas9 nAR: Cas9 nickase-based amplification reaction
Cascade: CRISPR-associated complex for antiviral defense
CAS-EXPAR: CRISPR/Cas9-triggered isothermal exponential amplification reaction
Cas-G4EX: CRISPR/Cas9 system-mediated G4-EXPAR
CASLFA: CRISPR/dCas9-mediated lateral flow nucleic acid assay
CaT-Smelor: CRISPR/Cas12a- and aTF-mediated small molecule detector
CD19: cluster of differentiation 19
CD70: cluster of differentiation 70
CDK-5: cyclin-dependent kinase 5
CHE: Switzerland
CHN: China
CLISA: CRISPR/Cas13a signal amplification linked immunosorbent assay
CNN: convoluted neural network
cOAs: cyclic oligonucleotides
CONAN: Cas3-operated nucleic acid detection
CPPs: cell-penetrating peptides
CRISDA: CRISPR–Cas9-triggered nicking endonuclease-mediated strand displacement amplification
CRISPR: clustered regularly interspaced short palindromic repeats
CRISPRa/i: CRISPR activation/interference
crRNA: CRISPR RNA
CRS: cytokine release syndrome
ctPCR: CRISPR-typing PCR
CVD: cardiovascular disease
dCas13: deficient Cas13
dCas9: dead Cas9
ddRPA: droplet digital RPA
DETECTR: DNA endonuclease-targeted CRISPR trans reporter
DGK: diacylglycerol kinase
DNMT: DNA methyltransferase
DSBs: double-stranded breaks
dsDNA: double-stranded DNA
EBV: Epstein–Barr virus
EGFR: epidermal growth factor receptor
EMA: European Medicines Agency
ESR1: estrogen receptor 1
EZH2: histone 3 lysine 27 methyltransferase
FAD: familial AD
FDA: food and drug administration
fDNA: functional DNA
FELUDA: FnCas9 Editor Linked Uniform Detection Assay
FFPE: formalin-fixed, paraffin-embedded
FH: familial hypercholesterolemia
FISH: fluorescence in situ hybridization
FLASH: finding low-abundance sequences by hybridization
FSHD: facioscapulohumeral muscular dystrophy
Gal3: galectin 3
GI: gastrointestinal cancer
GM-CSF: granulocyte-macrophage colony-stimulating factor
GRN: granulin
gRNA: guide RNA
GvHD: graft versus host disease
HAE: hereditary angioedema
HARRY: highly sensitive aptamer-regulated Cas14 R-loop for bioanalysis
hATTR: hereditary transthyretin amyloidosis
HBB: hemoglobin subunit beta
HBG1: hemoglobin subunit gamma 1
HBV: hepatitis B virus
HCC: hepatocellular carcinoma
HCR: hybridization chain reaction
HCV: hepatitis C virus
HD: histidine-aspartate
HDR: homology-directed repair
HeFH/HoFH: heterozygous/homozygous familial hypercholesterolaemia
HELP: HSV-1-erasing lentiviral particles
HEPN: higher eukaryotes and prokaryotes nucleotide-binding
HER2: human epidermal growth factor receptor 2
HIV/AIDS: human immunodeficiency virus/acquired immunodeficiency syndrome
HIV-1: human immunodeficiency virus, type 1
HPV: human papillomavirus
HSPCs: hematopoietic stem and progenitor cells
HTLV-1: human T cell lymphotropic virus, type 1
IARC: International Agency for Research on Cancer
IL-12: interleukin-12
IL1R1: interleukin receptors
IL3RA: interleukin 3 receptor alpha
indels: insertions or deletions
iPSCs: induced pluripotent stem cells
JPN: Japan
KLKB1: kallikrein B1
KO: knockout
KRAB: Krüppel-associated box transcriptional repression domain
KRAS: c-*K*_i_-Ras
KSHV: Kaposi’s sarcoma herpes virus
LAG3: lymphocyte activation gene-3
LAMA2: laminin alpha 2-chain
Lama2: laminin alpha 2
LAMP: loop-mediated isothermal amplification
LCA10: leber congenital amaurosis type 10
LNPs: lipid nanoparticles
Lp­(a): lipoprotein (a)
LRRK2: leucine rich repeat kinase 2
MDC1A: merosin-deficient congenital muscular dystrophy type 1A
MHRA: Medicines and Healthcare Products Regulatory Agency
MIT: Massachusetts Institute of Technology
MM: multiple myeloma
MRSA: methicillin-resistant *Staphylococcus aureus*
NA: not applicable
NASBA: nucleic acid sequence-based amplification
NASBACC: nucleic acid sequence-based amplification-CRISPR cleavage
nCas9: Cas9 nickase
NF-κB: nuclear factor κB
NHEJ: nonhomologous end joining
NHL: non-Hodgkin’s lymphoma
NK: natural killer
NOS: not specified
NSCLC: nonsmall cell lung cancer
OC: ovarian cancer
PADLOCK: picoinjection aided digital reaction unlocking
PAM: protospacer adjacent motif
PARP: poly­(ADP-ribose) polymerase
PB-19: parvovirus B19
PC: pancreatic cancer
PD: Parkinson’s disease
PD-1: programmed-death1
PD-L1: programmed death-ligand 1
PEI: polyethylenimine
PFS: protospacer flanking site
PGRMC1: progesterone receptor membrane component 1
PICASSO: CRISPR-based peptide display technology called peptide immobilization by dCas9-mediated self-organization
PINK1: PTEN-induced kinase 1
PLGA: poly­(lactic-*co*-glycolic acid)
Plk4: polo-like kinase 4
PNA: peptide nucleic acid
POIROT: photoinitiated CRISPR–Cas12a system for robust one-pot testing
PRKN: parkin RBR E3 ubiquitin protein ligase
PSEN1: presenilin-1
PSEN2: presenilin-2
qPCR: quantitative polymerase chain reaction
Rb: retinoblastoma
RBCs: red blood cells
RCasFISH: CRISPR/dCas9-MS2-based RNA fluorescence in situ hybridization assay
RNA-RBP: RNA-binding proteins
RPA: recombinase polymerase amplification
SaCas9: *Staphylococcus aureus* Cas9
SCAN: solid-state CRISPR/Cas12a-assisted nanopores
SCC: squamous cell carcinoma
SCD: sickle cell disease
sgRNA: single guide RNA
SHERLOCK: specific high-sensitivity enzymatic reporter unlocking
siRNA: small interfering RNA
SNCA: α-synuclein
SNPs: single-nucleotide polymorphisms
SOCS1: suppressor of cytokine signaling 1
SpCas9: *Streptococcus pyogenes* Cas9
SPRINT: SHERLOCK-based profiling of in Vitro transcription
ssDNA: single-stranded DNA
ssRNA: single-strand RNA
STOP: Sherlock testing in one pot
T1D: type 1 diabetes
TALEN: transcription activator-like effector nucleases
T-ALL: T cell acute lymphoblastic leukemia
TCL: T cell lymphoma
TET2: Tet methylcytosine dioxygenase 2
tgRNA: tuned guide RNA
TNF: tumor necrosis factor
TNFR1: tumor necrosis factor α receptor
TNFRSF17: TNF receptor superfamily member 17
TRAC: T cell receptor α subunit constant
tracrRNA: trans-activating crRNA
TTR: transthyretin
UCAD: ultrasensitive CRISPR/Cas12a-based antibody detection
UNIVERSE: universal nuclease for identification of virus empowered by RNA-sensing
USA: United States
USP1: ubiquitination-specific proteases
UTI: urinary tract infection
VEGF-A: vascular endothelial growth factor A
WIPO: World Intellectual Patent Office
ZFN: zinc finger nucleases
