# Supplementary material for: CRISPR Technology: Transforming the Future of Medicine and Diagnostics
Source: Biochemistry. 2025 Nov 24;64(24):4628–60. doi: 10.1021/acs.biochem.5c00480 (PMC12713731; doi:10.1021/acs.biochem.5c00480)
Supplement: Supplementary file 1 [file bi5c00480_si_001.pdf]

Supplementary Information for

CRISPR technology: Transforming the future of medicine and diagnostics

Kavita A Iyer [1†], Rumiana Tenchov[1†], Leilani M Lotti Diaz[1†], Preeti Jain[2†], Trupti Thite[2†], Yi Deng[1], Qiongqiong Angela Zhou[1*]

[1]CAS, A Division of the American Chemical Society, Columbus, Ohio 43210, United States

[2]ACS International India Pvt. Ltd., Pune 411044, India

[†]Equal contribution

*Corresponding author, email: qzhou@cas.org

- Methods
- CRISPR/Cas: Biology and mechanism
- Types of CRISPR/Cas systems
- Patent activity
- Commercial interest in CRISPR
- CRISPR in agriculture
- AI in CRISPR
- Tables S1-S7
- Figures S1-S16
- References

Methods

Search query: The search query crafted by CAS subject matter experts and adjusted iteratively to minimize noise while ensuring maximum coverage of the field. The query included the following terms – Crispr? or Cas9? or CRISPR-Cas system or Cas12 or CRISPR-associated(w)protein(w)9 Or clustered(1w)regularly(1w)interspaced(1w)short(1w)palindromic(1w)repeat or Crispr(w)tool(w)kit or Crispr-Cas(w)transposase(w)system or Molecular(w)sciss? Or genetic(w)sciss?

Data extraction: Data was extracted from the CAS Content Collection and broadly speaking consisted of document, concept and patent activity data. In terms of document data for journal publications, information included title, abstract, year of publication, citations, authors and research institutions. For patent publications, information pertaining to patent offices and patent assignees were also included in addition to other pertinent information. Concept data consisted of CAS indexed terms and their roles associated with publications. Finally, patent activity data consisted of comprehensive information about individual patents in patent families and their filing across 97 patent offices worldwide.

Data analysis: The first step of the analysis consisted of separating/filtering out publications associated with CRISPR in agriculture. To achieve this, we filtered out the following terms related to agriculture in the title and abstract of documents using Tableau: “agricul”, "crop”, "soybean, "corn, "potato”, "rice”, "fertilizer”, "soil amend”, "wheat", "bean”, and "legume”. We also filtered out the word “plant” just from title and not abstract since removal from abstract resulted in loss of documents within our scope. Error of false positives was calculated at <5%.

Leading patent assignees were identified based on volume of patents filed and separated into commercial and non-commercial patent assignees. Leading journal publishers and research organizations were identified on the combination of volume of research published and the average number of citations per publication. Geographical information (country/region) and organizational affiliation was that of the first author or the first patent assignee for journal and patent publications, respectively. In general, data analysis was performed by searching for keywords in the title, abstract, and CAS indexed terms associated with publications and included multiple synonyms as well as abbreviations.

Data visualization: Figures and images were created using a combination of Tableau, Microsoft Excel, and Adobe illustrator. Additionally, www.BioRender.com was also utilized for creating some of the schematic illustrations and has been specified in Figure captions.

CRISPR/Cas: Biology and mechanism

To fully understand CRISPR, it is essential to break down its components, and the mechanism of its natural function in prokaryotes to understand the way CRISPR can be exploited to achieve genome editing capabilities in humans and other organisms.

How CRISPR is employed by prokaryotic organisms as a natural defense

In 1987, the presence of CRISPR, in the *Escherichia coli* genome was reported by Nakata *et al.* from Osaka University, Japan[1] while Cas was discovered in 2002 by Jansen *et al.*[2] The subsequent discovery of CRISPR systems in both Gram-positive and Gram-negative bacteria, along with archaea, led to questions regarding the relevance of CRISPR to these organisms.[3, 4] Later on, in the mid-2000s, the functionality and importance of CRISPR was first realized in prokaryotes wherein the CRISPR system is a key component of their adaptive immunity, which protects prokaryotes from attack by viruses, plasmids, and other invasive genetic elements.[5-7]

The CRISPR defense mechanism protects bacteria via three basic stages: spacer acquisition (adaptation), CRISPR RNA (crRNA) biogenesis (expression or maturation), and target interference[8-10] (**Figure S1**). CRISPR/Cas systems are composed of a *Cas* operon and a CRISPR array. The CRISPR array contains unique sequences (spacers) flanked by palindromic repeats. These spacers are derived from foreign DNA that originated from mobile genetic elements such as bacteriophages, transposons, or plasmids that have previously infected the prokaryote. Cas (CRISPR-associated nuclease proteins) genes are usually found adjacent to the CRISPR array and encode the nuclease proteins (Cas proteins) responsible for destroying or cleaving viral nucleic acid.[7, 11-13]

In the first stage (spacer acquisition), upon phage infection, a sequence of the invading DNA (protospacer) is incorporated into the CRISPR array by the Cas1–Cas2 complex to form immune memory. In the second stage referred to as CRISPR RNA (crRNA) biogenesis, when foreign nucleic acids invade again, the CRISPR array is transcribed to generate crRNA. In type II systems, crRNA pairs with a complementary trans-activating crRNA (tracrRNA), while other systems use different RNA processing mechanisms. At the interference stage, the crRNA guides Cas proteins to specifically destroy the invading nucleic acid.[49-52] Adjacent to the crRNA-targeted sequence on the invading DNA, a short sequence referred to as the protospacer adjacent motif (PAM) plays an essential role in the adaptation and interference stages. For DNA targeting Cas proteins (such as Cas9 and Cas12a), PAM recognition is essential for target DNA binding and cleavage. The PAM specificity is determined by amino acids within the PAM-interacting domain of the Cas protein itself. This PAM requirement prevents self-targeting of the host CRISPR array (which lacks PAM sequences) while enabling recognition and cleavage of invading DNA that contains appropriate PAM sequences.[14]

How this bacterial phenomenon was adapted to be used as a programmable toolkit for genome editing

In 2012, Jennifer Doudna and Emmanuelle Charpentier, a molecular biologist and microbiologist, respectively, were the first to propose that the bacterial CRISPR/Cas9 system could be used as a programmable toolkit for genome editing in humans and other animal species. For their path breaking work, they were eventually awarded the Nobel Prize in Chemistry in 2020.[15] Another

breakthrough was the synthesis of single gRNA (sgRNA) which combines the role of crRNA and tracrRNA into a single molecule by fusing them together with a linker.[16, 17] The mechanism of CRISPR/Cas9 genome editing contains three steps, recognition, cleavage, and repair.[18] The designed sgRNA directs Cas9 and recognizes the target sequence in the gene of interest through its 5'crRNA complementary base pairing. The Cas9 protein remains inactive in the absence of sgRNA. The Cas9 nuclease makes double-stranded breaks (DSBs) at a site 3 base pairs upstream of the PAM.[19] PAMs are short, conserved 2–5 base-pair sequences located immediately adjacent to the target site. Their specific sequence varies depending on the Cas9 ortholog and bacterial species of origin. Once Cas9 has found a target site with the appropriate PAM, it triggers local DNA melting followed by the formation of an RNA-DNA hybrid. Then, the Cas9 protein is activated for DNA cleavage.

Finally, the DSB is repaired by either non-homologous end joining (NHEJ) or homology-directed repair (HDR) cellular mechanisms.[20, 21] The NHEJ pathway repairs DSBs in DNA by directly ligating through an enzymatic process without the need for exogenous homologous DNA template. The NHEJ mechanism often introduces insertions or deletions (indels) at the junction.[22] In contrast, the HDR process, although more complex than NHEJ, uses a homologous DNA template. The homologous DNA template has homology to the sequences flanking the site of cleavage to incorporate new DNA fragments. The template guides the repair process and lowers the possibility of errors. Since there is no insertion or deletion of nucleotide sequences, the HDR pathway maintains uniformity in the size of the resulting double-stranded DNA (dsDNA), unlike NHEJ.[23]

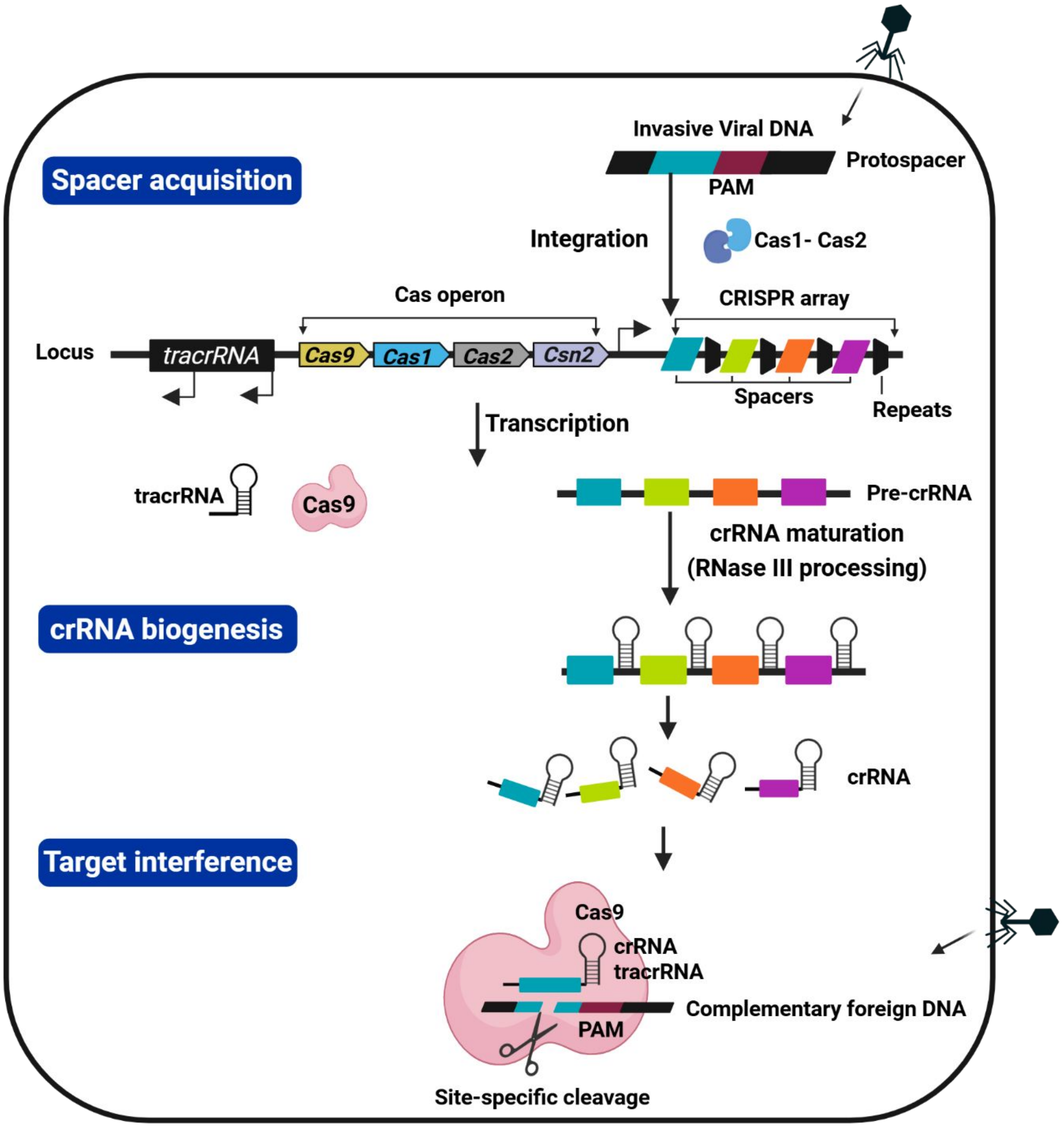

Figure S1. Mechanism of CRISPR/Cas9 adaptive immunity. The CRISPR/Cas systems are composed of a *Cas* operon and a CRISPR array that comprises identical repeat sequences that are interspersed by phage-derived spacers. The CRISPR/Cas mediated adaptive immunity consists of three stages, namely, spacer acquisition, crRNA biogenesis and target Interference. Abbreviations used: PAM – protospacer adjacent motif (PAM), crRNA – CRISPR RNA, tracrRNA – trans-activating crRNA. Figure was created using www.BioRender.com.

Types of CRISPR/Cas systems

The bacterial and archaeal CRISPR/Cas systems of adaptive immunity show remarkable diversity of protein composition, effector complex structure, genome locus architecture, mechanisms of adaptation, pre-crRNA processing and interference. CRISPR/Cas systems have been divided

largely into two classes, 6 types and 48 subtypes[24] (summarized in **Figure S2** and **Table 1**). Class 1 systems include Types I, III, and IV, that use multiprotein complexes to destroy foreign nucleic acids, and class 2 systems include Types II, V, and VI, that use single proteins.[25] Recent studies have reported a candidate Type VII system, a novel addition to Class 1, comprising an RNA-targeting complex formed by Cas5/Cas7-like proteins that recruits a Cas14 nuclease.[26] Its provisional assignment as a distinct CRISPR-Cas type is still under evaluation, and its acceptance as a canonical type awaits further experimental validation.

In this section, we provide a brief overview of natural Cas nucleases and engineered variants that have been adopted for use as therapeutics (**Figure S2** and **Table S1**).

Class 1 CRISPR/Cas systems

Class 1 systems require multiple Cas proteins to come together in a complex to mediate interference against foreign genetic elements making gene editing applications of class 1 systems limited. Class 1 systems are further divided into three CRISPR/Cas types based on the presence of a specific signature protein: Type I contains Cas3, Type III contains Cas10, and the Type IV contains Csf1, a Cas8-like protein.[27] Please see Table 1 for key differences and similarities between the different Class I CRISPR/Cas systems.

Type I is currently divided into seven subtypes, I-A to I-G; there are three distinct variants of the I-F subtype (I-F1, I-F2, and I-F3). A characteristic of Type I systems is CRISPR-associated complex for antiviral defense (Cascade), , which is a complex encompassing Cas molecules and crRNA.[28] The Cascade proteins of Type I systems typically encompass Cas5, Cas6, Cas7, and Cas8 (Cse1), with some subtypes also presenting Cas4 and/or Cas11(Cse2).[29] The pre-crRNA is processed by Cas6. In the interference stage of Type I systems, Cascade recognizes the PAM sequence, targets DNA in a sequence-specific manner through crRNA, and uses Cas3 to generates a single-strand nick on the unwinding target DNA thereby degrading the displaced strand through its 3′–5′ exonucleolytic activity.[30]

The overall composition and structure of Type III are highly similar to the Type I system.[31] It utilizes multi-subunit effector complexes to cleave both RNA and DNA and has been classified into six subtypes: III-A to III-F.[24] In the Type III system, the signature protein is multidomain Cas10 with an N-terminal histidine-aspartate (HD) nuclease domain (several subtypes) and two palm domains (a type of the RNA recognition motif). The HD domain takes part in nonspecific single-stranded DNA (ssDNA) cleavage activity, whereas the palm domain catalyzes the conversion of ATP to cyclic oligonucleotides (cOAs) when the type III crRNA-guided effector complex recognizes the target RNA. The cOAs activate the Csm6 protein, which nonspecifically degrades RNA molecules.[32, 33]

Type IV CRISPR/Cas system is divided into IV-A, IV-B, and IV-C subtypes, which mainly exist in plasmids however, their specific function is still poorly characterized.[24] Type IV lack certain hallmark components of other CRISPR–Cas systems, including the highly conserved adaptation module and an effector nuclease. All Type IV systems contain Cas7 protein (Csf2) and Cas5 protein (Csf3), which are part of the multi-subunit complex. Type IV-A CRISPR/Cas carry a DinG helicase instead of a nuclease component. Type IV CRISPR/Cas system may have the potential to be used in clinical drug-resistance gene therapy.[34, 35]

Class 2 CRISPR/Cas systems

Unlike Class 1 CRISPR/Cas systems, which utilize multiprotein effector complexes, Class 2 systems rely on a single multidomain protein for interference.[70] Class 2 CRISPR/Cas systems are divided into three major types: Type II, Type V and Type VI (please see **Table S1** for subtype comparisons).

Type II CRISPR/Cas systems are subdivided into II-A, II-B, II-C and II-D subtypes.[36] The II-C subtype has two variants (II-C1, II-C2).[24] These systems use a single large multidomain Cas9 protein as the effector complex. All Type II CRISPR/Cas loci contain *cas1* and *cas2* genes (essential for the CRISPR adaptation) and tracrRNA (noncoding RNA, required to mature the long pre-crRNA and for interactions with Cas9 protein). The crRNA–tracrRNA–Cas9 protein complex recognizes and cleaves the target DNA sequences. Cas9 nucleases recognize the PAM sequence (5'-NGG-3'), and induce blunt-end DSBs at target sites.[37-39] Cas9 has two distinct nuclease domains, HNH and RuvC, which cleave the target and non-target strand, respectively[16, 40] (**Figure S2B** and **Table 1**). Among the different types of CRISPR/Cas systems, the Type II system is the most widely used in genetic engineering due to its simplicity, versatility, and efficiency.[41] The engineered CRISPR/Cas9 system encompasses two main components: a sgRNA and Cas9 protein. The sgRNA is a fusion of tracrRNA and crRNA, containing a 20-nucleotide spacer sequence that is complementary to the target sequence and an ~80-nucleotide scaffold that enables Cas9 binding. The target DNA sequence should be adjacent to the PAM site in the genome. Inactivation of either nuclease domain of Cas9 creates a Cas9 nickase[42] (nCas9), useful in base editors and prime editors, which performs precise genome editing without requiring DSBs,[43] and dCas9 serves as a scaffold for recruiting effectors proximal to specific genomic sites. dCas9 is widely used for regulating transcription, altering epigenetic controls, imaging living cells, and other purposes.[44, 45]

Type V CRISPR/Cas system can be divided into V-A to V-I, V-K, and V-U subtypes with diverse functions and its effectors are Cas12a (Cpf1), Cas12b (C2c1), Cas12c (C2c3), etc.[24] Type V CRISPR/Cas system requires Cas and crRNA to edit the target site. Once the crRNA recognizes the PAM site (5’-TTTN-3’) and fully pairs with the target DNA base, Cas12a uses the RuvC domain to cut the target sequence in a cis manner to generate the 5’ sticky end, and at the same time, uses the trans cutting activity to cut any adjacent ssDNA.[46] Cas12a shows RNase III activity, does not require a tracrRNA, and generates mature crRNA.[47] Like Cas12a, Cas14a belongs to the Class 2 system Type V family. Cas14a proteins were initially characterized for their RNA-guided, PAM-independent ssDNA-targeted endonuclease activity.[48, 49] However, recent studies have demonstrated that Cas14a can also cleave double-stranded DNA in a PAM-dependent manner (5' T- or C-rich PAM) under certain conditions.[50]

Type VI CRISPR/Cas system can be divided into VI-A, VI-B, VI-C, and VI-D subtypes.[24] The signature protein is Cas13 (C2c2), which has higher eukaryotes and prokaryotes nucleotide-binding (HEPN) domains.[51] The uniqueness of this system is that Cas13 can recognize ssRNA molecular targets. Under the targeting effect of crRNA without the requirement of tracrRNA, the Cas13-crRNA complex recognizes the sequence of the protospacer flanking site (PFS) on the target nucleic acid, and at the same time cuts the target RNA, trans cuts the single-strand RNA (ssRNA).[52-54]

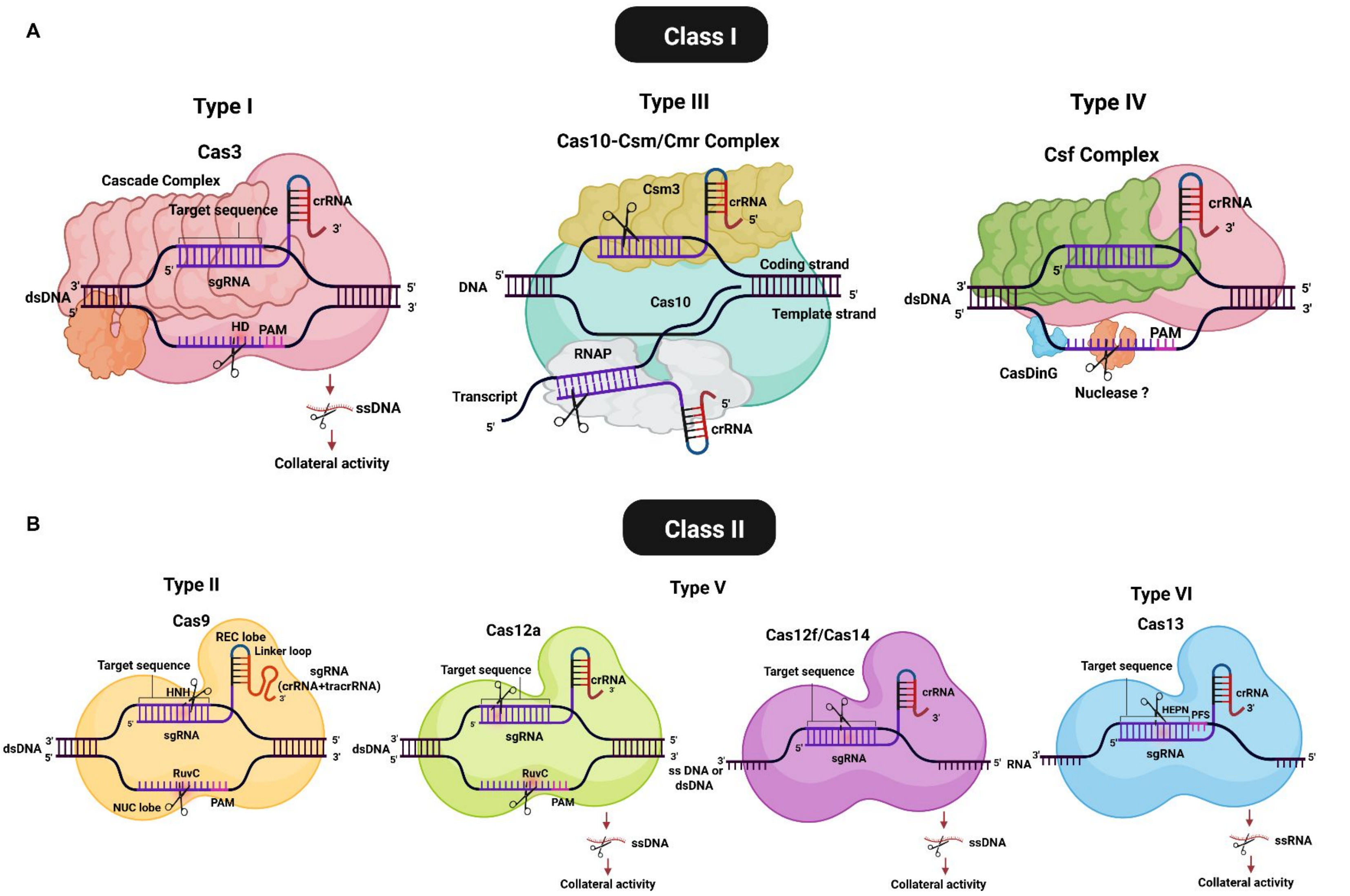

Figure S2. Schematics depicting various CRISPR/Cas system types and cleavage characteristics. The CRISPR/Cas system is classified into two classes based on the effector molecules involved. (**A**) Class I is characterized by multi-unit effectors, while (**B**) Class II exhibits a single effector. Each class is further divided into three types distinguished by their catalytic domain and target nucleotide specificity. The illustration was created using BioRender.com.

Table S1. Classification of CRISPR/Cas system with respect to various parameters including their class, types, subtypes, protein, nuclease domain, protospacer restriction, pre-crRNA processing, effectors, tracrRNA requirement, target and cleavage activity.

Class	Type	Sub-types	Signature Protein	Nuclease domain	Protospacer restrictions	Pre-crRNA Processing	Effectors of CRISPR system	tracrRNA	Target and Cleavage activity
I	I	A-G	Cas3	N-terminal histidine-aspartate (HD) domain	PAM-like NTT sequences	Cas6	Cas3, Cascade, crRNA	No	Nuclease activity to ssDNA
	III	A-F	Cas10	RRM	No PFS bias	Cas6	Cmr/Csm, crRNA, Cas10	No	Nuclease activity to ssDNA/RNA
	IV	A, B	Csf1	-	5′-GNAWN-3′ on the 5′-side of the target	Cas6	Cas7 (Csf2), Cas5 (Csf3) , Cas8 (Csf1), crRNA, CasDinG	No	Unknown
II	II	A-C	Cas9	HNH and RuvC domains	PAM, 3′ G-rich motif	RNase III, tracrRNA	Cas9, tracrRNA, crRNA	Yes	dsDNA blunt cleavage activity and no collateral cleavage activity
	V	A-I, K, U	Cas12	RuvC domain	PAM, 5′ T-rich motif	Cpf1	Cpf1, crRNA, tracr RNA	No	dsDNA overhang cleavage activity and ssDNA collateral cleavage activity
			Cas12f (also known as Cas14)	RuvC domain	PAM independent, Use T-rich sequences as PAMs	Yes	Cpf1, tracrRNA, crRNA	Yes	ssDNA, dsDNA and ssDNA collateral cleavage activity
	VI	A-D	Cas13	2 HEPN domains	PFS - varies by subtype; Cas13a: 3' PFS (A, U, or C); Cas13b: 5' and 3' PFS; Cas13d: No PFS requirement	Self-processes its own pre-crRNA array using endonuclease activity	C2c2, crRNA	No	ssRNA and non-specific RNA cleavage upon target recognition (collateral activity)

Patent activity

We next took a look at patent activity data in the field of CRISPR therapeutics; an activity is defined as an event that leads to the publication of a patent document, for example application publications, issuing of patents, and others. An analysis of patent family activity data in **Figure S3** shows the flow from the patent assignee country (left) to the patent office where the first application in a given family is filed (center) and finally to the destination patent office for individual patent publications within the family. The United States leads in terms of sheer volume of patent publications, most of which are sent to the World Intellectual Patent Office (WIPO). Still, a decent sized portion of US patents go straight to the home office. For China, the second country/region leading in terms of sheer volume of patent publications, an overwhelming majority of patent applications appear to have been filed and granted at their home office. Most of the patents initially submitted to WIPO are subsequently submitted to the U.S., the European patent Office, Canada, Japan, South Korea and China, with a smaller number being filed at patent offices in India, Brazil, Australia, and other smaller patent offices around the world.

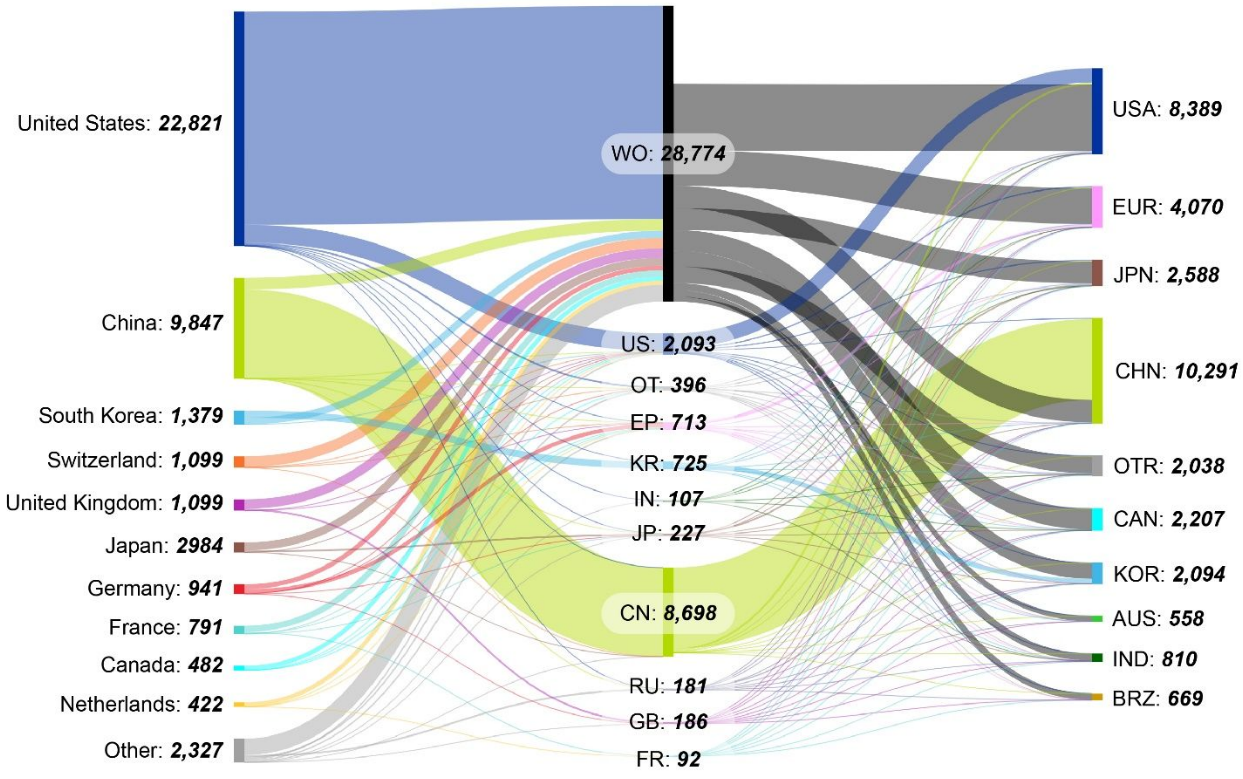

Figure S3. Patent family activity data related to patents in the field of CRISPR for the period 1995-2024 based on data from the CAS Content Collection. The column on the left corresponds to country/region of the patent assignee, the column in the middle corresponds to the first patent office and the column on the right corresponds to the final patent office. Note that data for 2024 is incomplete due to time of data extraction and encompasses data for January to June.

Commercial interest in CRISPR

In the past decade, capital investment in the field of CRISPR technology has seen a remarkable increase with a sharp increase starting in 2018 and persisting until 2021 with investments exceeding a staggering USD 11 billion in 2021 (**Figure S4A**; PitchBook Data, Inc.; *Data has not been reviewed by PitchBook analysts.). An overwhelming majority of these investments involved companies originating in the United States (USA, 96%). Other key players in terms of geographical distribution, though of much smaller magnitude, included Switzerland (CHE), China (CHN) and Japan (JPN) (**Figure S4B**).

Breakdown of the capital invested by primary industry code, a system of classification indicative of a business's main line of revenue, shows that as expected biotechnology, pharmaceuticals and drug discovery, together account for almost 90% of capital invested (Figure 25C). Other industries with smaller contributions include agricultural chemicals, diagnostic equipment and laboratory services among others (**Figure S4C**). This is in line with our analysis which also identified CRISPR therapeutics and CRISPR in agriculture as the major applications. Time trends of capital invested in these sectors indicate largest influx of capital in the biotechnology industry with two major spikes in 2019 and 2021. A record number of deals occurred in 2021 and few of the biggest (in terms of deal size) involved Century Therapeutics and Mammoth Biosciences (>150M USD) and, Caribou Biosciences and AgBiome (>100M USD). The most consistent influx in capital on the other hand appears to be in drug discovery until 2021 with a plunge in 2022 and uptick again after 2022 (**Figure S4D**).

A few key players in the biotechnology space with a high number of patents related to CRISPR are: CRISPR Therapeutics, Caribou Biosciences and Intellia Therapeutics, while those in pharmaceuticals and drug discovery space are Regeneron Pharmaceuticals, Editas Medicine, Beam Therapeutics and Metagenomi, among many others. Regeneron Pharmaceuticals, most well-known for its AAV vectors for gene delivery, in collaboration with Intellia Therapeutics is actively pursuing development of CRISPR/Cas9-based therapeutics for hemophilia A and B,[55] a type of rare bleeding disorder.[56] Regeneron Pharmaceuticals appears to also be engaged in collaboration with Mammoth Biosciences[57] co-founded by the Nobel Laureate and CRISPR pioneer Jennifer Doudna.

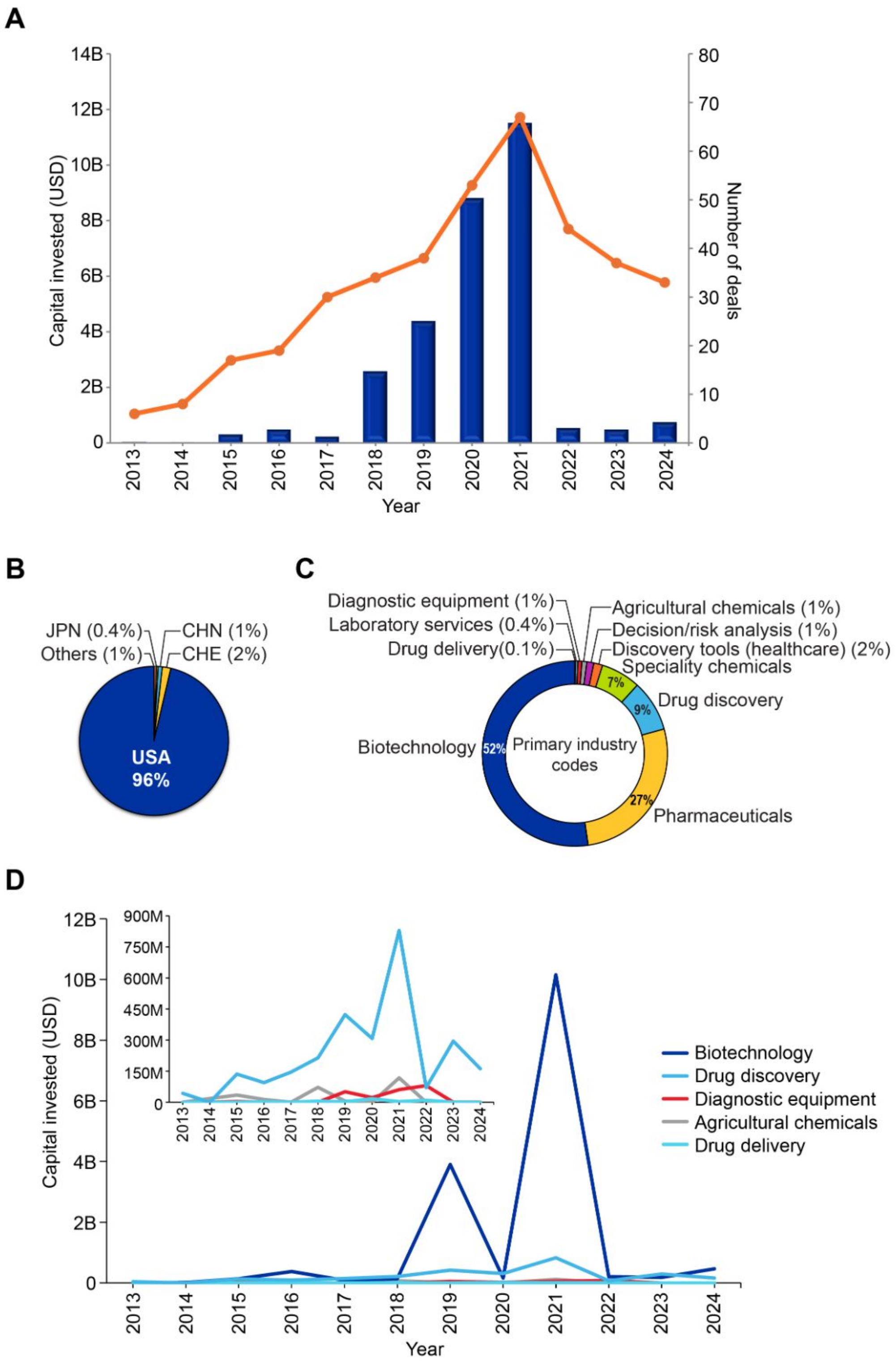

Figure S4. Commercial interest in CRISPR technology. (**A**) Capital invested in and number of deals in CRISPR. (**B**) Geographical distribution of capital invested in CRISPR. (**C**) Breakdown of capital invested as per primary industry codes and (**D**) their time trends over the last decade (2013-2024). Source: PitchBook Data, Inc.; *Data has not been reviewed by PitchBook analysts.

CRISPR in agriculture

CRISPR/Cas is also considered as the leading site-specific nuclease for plant genome editing, first reported in 2013 by Li *et al.*,[58] Shan *et al.*,[59] and Nekrasov *et al.*[60] Not only is it useful to develop crops that are high yielding and resistant to abiotic stresses (drought, salinity, flooding, etc.) exacerbated by climate change, but also to resist biotic stresses such as insects and pathogens that are also being exacerbated by climate change. Our original search query resulted in a considerable number of documents, specifically around 13%, related to the application of CRISPR in plants and for agricultural use. The use of CRISPR in agriculture is beyond the scope of this manuscript and the related documents were filtered out of our dataset. For more information on the application of CRISPR for the enhancement of disease and parasitic plant resistance in crops and/or for climate change resilience we suggest reviews by: Jhu *et al.*,[61] Paul *et al.*,[62] Khan *et al.*,[63] Zaidi *et al.*,[64] Karavolias *et al.*,[65] Ndudzo *et al.*,[66] Misra *et al.*,[67] Schenke and Cai,[68] Ahmad *et al.*,[69] and Maximiano and Franco.[70]

AI in CRISPR

With the recent and ongoing surge in artificial intelligence (AI) and its application in a wide range of fields, interest in using AI in CRISPR has also seen an increase as exhibited by the growth of publications over the last decade (**Figure S5**).

Discussed briefly below are a few examples of AI models developed for CRISPR:

- DeepCas9,[71] a deep convoluted neural network (CNN) model consisting of a combination of several other deep-learning models and capable of predicting on-target activity of sgRNAs based on DNA sequences potentially allowing high-throughput screening of sgRNAs.
- OpenCRISPR-1,[72] a protein language trained on several components of naturally occurring CRISPR systems with an emphasis on Class 2 systems (specifically the Types II system). The model was capable of designing diverse novel Cas9-like proteins not found in nature along with functional sgRNAs for said novel Cas9-like proteins. The model was generated using ProGen2, a protein large language model,[73] and trained using the CRISPR-Atlas (collection of Cas proteins, CRISPR arrays, tracrRNAs and PAMs from microbial genome).
- TIGER (Targeted Inhibition of Gene Expression via guide RNA design)[74] – A CNN-based model developed for predicting on- and off-target efficacy of Cas13d gRNAs. Potential applications of the TIGER model include reducing undesirable off target effects thereby improving safety profile of CRISPR therapeutics.
- EVO,[75] a genomic foundational model trained on >82,000 CRISPR/Cas loci capable of producing unique Cas proteins (Cas9, Cas12 and Cas13).
- Other models/tools include: CRISPick,[76-78] sgRNA Scorer,[79, 80] SSC,[81, 82] DeepCRISPR,[83, 84] CRISTA,[85] CRISPR MultiTargeter,[86, 87] DeepHF,[88, 89] CRISPR-A-I,[90] CRISPR-P[91, 92] and many more.

For a comprehensive review on the use of AI in CRISPR please see Dixit *et al.*[93] and Lee.[94]

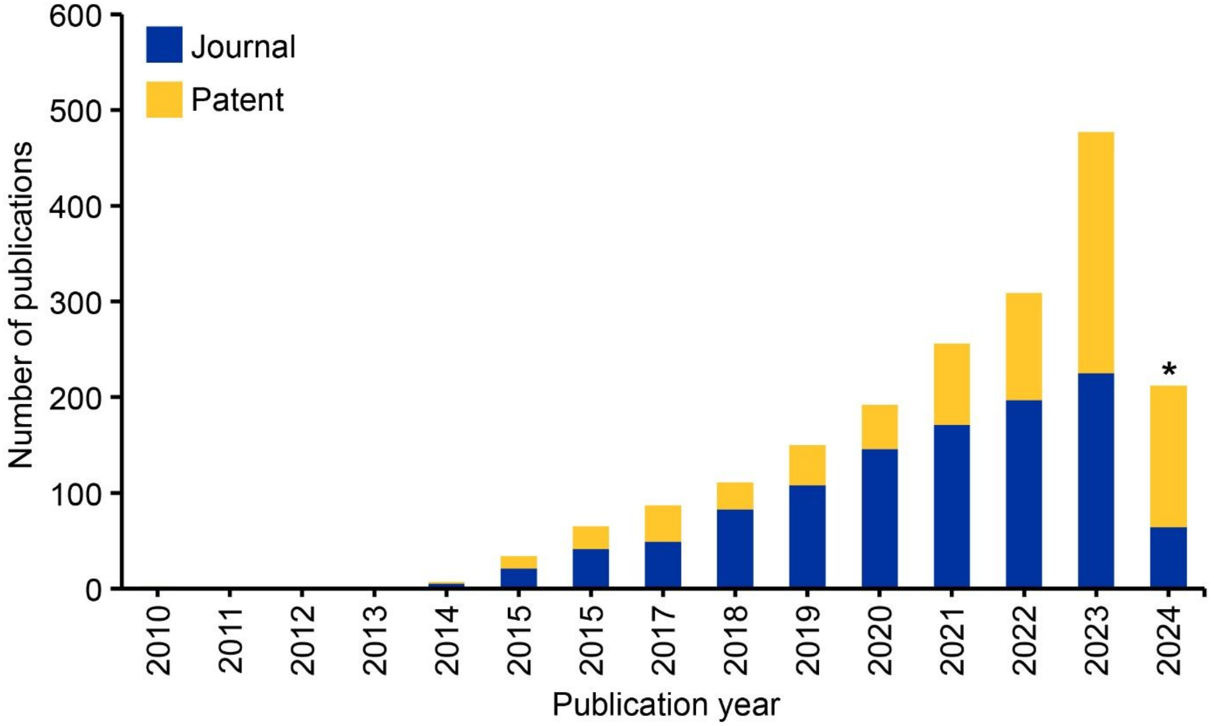

Figure S5. Time trends of publications related to artificial intelligence (AI) in the CRISPR dataset. Data includes journal and patent publications from the CAS Content Collection for the period 2010-2024. *Note that data for 2024 is incomplete due to time of data extraction and encompasses data for January to June.

Table S2. List of CRISPR/Cas therapeutics currently in the developmental pipeline.

Drug Name	Company	Gene Target	Disease Condition	Status | Year	Clinical Trial ID
**Phase I**					
AVC-201/ Allo-RevCAR01-T-CD123	AvenCell Therapeutics, Inc.	Interleukin 3 receptor subunit alpha	Relapsed/refractory acute myeloid leukemia (AML) and other selected hematologic malignancies positive for CD123	Recruiting | 2023	NCT05949125
CB-010	Caribou Biosciences, Inc.	CD19 molecule	Follicular lymphoma and non-Hodgkin lymphoma	Recruiting | 2020	NCT04637763
CB-011	Caribou Biosciences, Inc.	TNF receptor superfamily member 17	Multiple myeloma	Recruiting | 2023	NCT05722418
CTX-310	CRISPR Therapeutics	Angiopoietin like 3	Cardiovascular diseases such as mixed dyslipidemias, homozygous familial hypercholesterolemia, heterozygous familial hypercholesterolemia and severe hypertriglyceridemia	Prospectively registered | 2023	ACTRN12623000809639
CTX-320	CRISPR Therapeutics	Lipoprotein(a)	Cardiovascular disease	Prospectively registered | 2023	ACTRN12623001095651
ET-01	EdiGene	Not applicable	β-thalassemia, sickle cell anemia, and iron granulocyte anemia	Active, not recruiting | 2021	NCT04925206
HG-202	HuidaGene Therapeutics	Vascular endothelial growth factor A	Wet age-related macular degeneration	Recruiting | 2023	NCT06031727
JWK-001	Chengdu Jinweike Biotechnology Co., Ltd.	Vascular endothelial growth factor A	Neovascular form of age-related macular degeneration (nAMD) and diabetic retinopathy (DR)		CTR20241792
KSQ-4279/ RO7623066	KSQ Therapeutics, Inc.	Ubiquitin specific peptidase 1	Solid tumors including ovarian and triple-negative breast cancer	Recruiting | 2022	NCT05240898
P-BCMA-ALLO1	Poseida Therapeutics, Inc. and Roche	TNF receptor superfamily member 17	Multiple myeloma	Recruiting | 2021	NCT04960579

RM-001	Reforgene Medicine	Unspecified	β-thalassemia		Ref
RP-1664	Repare Therapeutics Inc.	Polo like kinase 4	Solid cancers	Recruiting | 2024	NCT06232408
SNIPR-001	SNIPR Biome	Not applicable	Bloodstream infections in hematological cancer patients and patients with autoimmune diseases	Completed | 2022	NCT05277350
YOLT-101	YolTech Therapeutics	Unspecified	Familial hypercholesterolemia	Recruiting | 2024	NCT06458010
**Phase II**					
BRL-201/ CD19-UCART	Bioray Laboratories	CD19 molecule, programmed cell death 1	B-cell lymphoma and non-Hodgkin's lymphoma	Suspended | 2017	NCT03229876
CTX-112	CRISPR Therapeutics	CD19 molecule	B-cell malignancies and systemic lupus erythematosus	Recruiting | 2022	NCT05643742
CTX-131	Nkarta, Inc. and CRISPR Therapeutics	CD70 molecule	Solid cancer, hematological, cancer, B-cell lymphoma, T-cell lymphoma,	Recruiting | 2023	NCT05795595
EBT-101	Excision BioTherapeutics, Inc.	Unspecified	HIV/AIDS	Completed | 2021	NCT05144386
ET-02	EdiGene, Inc.	CD19 molecule	Acute lymphocytic leukemia and non-Hodgkin's lymphoma	Active, not recruiting | 2021	NCT04933825
γ-Globin reactivated autologous hematopoietic stem cell therapy	Bioray Laboratories	Hemoglobin subunit γ 1,2	Thalassemia	Completed | 2019	NCT04211480
KSQ-001EX	KSQ Therapeutics, Inc.	Suppressor of cytokine signaling 1	Head and neck cancer, lung cancer, non-small cell cancer, melanoma cancer, solid cancer	Recruiting | 2024	NCT06237881
NTLA-2002	Intellia Therapeutics, Inc.	Kallikrein B1	Hereditary angioedema	Active, not recruiting | 2021	NCT05120830
Nulabeglogene autogedtemcel	Kamau Therapeutics	Not applicable	Sickle cell anemia	Recruiting | 2021	NCT04819841

OTQ-923	Novartis	Not applicable	Sickle cell disease	Active, not recruiting | 2020	NCT04443907
Renizgamglogene autogedtemcel (EDIT-301)	Editas Medicine, Inc.	Hemoglobin subunit beta	β-thalassemia and sickle cell anemia	Active, not recruiting | 2021	NCT04853576
Tumor infiltrating lymphocyte (TIL) therapy	Intima Bioscience, Inc.	Not applicable	Gastrointestinal cancers	Not yet recruiting | 2022	NCT05566223
VCTX-211	CRISPR Therapeutics and Vertex Pharmaceuticals	Not applicable	Type 1 diabetes	Recruiting | 2023	NCT05565248
VGB-Ex-01	Shanghai Vitalgen BioPharma Co., Ltd	Not applicable	Transfusion-dependent β-thalassemia (β-TDT)	Recruiting | 2023	NCT06041620
**Phase III**					
LBP-EC01	Locus Biosciences	Not applicable	Urinary tract infections caused by the *E. coli* and *K. pneumoniae*	Completed | 2019	NCT04191148
NTLA-2001	Intellia Therapeutics and Regeneron Pharmaceuticals	Transthyretin	Transthyretin amyloidosis and other liver disorders	Active, not recruiting | 2020	NCT04601051

Table S3. Comparison of the three molecular diagnostic methods.

Method	Time required	Response procedures	Advantages	Disadvantages
Next generation sequencing (NGS)	20 h	Library preparation, NGS sequencing, bioinformatics analysis	Comprehensive analysis of all nucleic acids, rapid and preliminary identification of new pathogens	Expensive equipment, complexity of operation, and not all genomes were available
Polymerase chain reaction (PCR)	1.5 h	Reverse transcription, RNA-cDNA hybridization denaturation, PCR amplification	Gold standard, currently the most common detection method	Complex laboratory infrastructure required, specialized technical personnel
CRISPR/Cas	0.6 h	DNA amplification, Cas reaction	Low cost, high sensitivity, no need for complex instruments and equipment, fast and convenient for field testing	Not widely used in clinical trials, pending clinical validation

Table S4. Key mechanisms, advantages, drawbacks, and detection platforms of CRISPR/Cas-based disease diagnostics.

Cas protein involved	Mechanism	Advantages / drawbacks	Detection platforms
Cas9	▪ sgRNA directs the Cas9 protein to a specific DNA sequence, where it creates a blunt DSB in DNA *in vitro*<br><br>▪ HNH and RuvC domains cleave complementary and non-complementary strands, respectively	▪ ***Drawback***:<br>- Cas9 protein is always used as an independent element to recognize, adhere to, or cut the target sequence, but does not participate in the whole detection process because it cannot easily indicate the result of the reaction without the support of other technologies	▪ **NASBACC**: Nucleic acid sequence-based amplification (NASBA)-CRISPR cleavage<br>▪ **CAS-EXPAR**: CRISPR/Cas9-triggered isothermal exponential amplification reaction<br>▪ **FLASH**: Finding low abundance sequences by hybridization [313]<br>▪ **FELUDA**: FnCas9 editor linked uniform detection assay (Milenia Biotec) [309]
nCas9	▪ When the nCas9-sgRNA complex binds to the target DNA and forms an R-loop, the ssDNA can be cleaved by the nickase, releasing 30 termini available for further manipulation,[315] which serve as ready substrates for strand extension and displacement reactions	▪ ***Advantage:***<br>- Efficient genome engineering with minimal off-target effects[95]	▪ **Cas9nAR**: Cas9 nickase-based amplification reaction[317]<br>▪ **CRISDA**: CRISPR/Cas9-triggered nicking endonuclease-mediated strand displacement amplification [318]
dCas9	▪ Incorporation of D10A and H840A mutations into Cas9 resulted in the generation of a nuclease-deficient Cas9 (dCas9) protein, which retains its DNA targeting ability while abolishing DSB formation and acquiring additional localization functionality[75]	▪ ***Advantage:***<br>- The frequency of off-target effects is lower than the conventional CRISPR[96]	▪ **CASLFA**: CRISPR/dCas9-mediated lateral flow nucleic acid assay[319]<br>▪ **dCas9-DNA-FISH**: CRISPR-mediated DNA-fluorescence *in situ* hybridization (FISH) [322]<br>▪ **Paired dCas9** platform[97]<br>▪ **RCasFISH**: CRISPR/dCas9-MS2-based RNA fluorescence *in situ* hybridization assay[324]<br>▪ **PICASSO**: Peptide immobilization by dCas9-mediated self-organization[323]<br>▪ **Bio-SCAN**: Biotin-coupled specific CRISPR-based assay for nucleic acid detection[320]
Cas12a	▪ A single mature crRNA (40–44 nt in length) guides Cas12a which binds upstream of a typical thymidine-rich PAM TTTV (where V = A, C and G) and cleaves DNA 18–23 nt distal to the PAM via 5 bp staggered DSBs [80, 325] resulting in DNA sticky ends (cis-acting), and it	▪ ***Advantage***:<br>- Potential to be used as a nucleic acid detection system through the incorporation of a ssDNA reporter molecule	▪ **DETECTR**: DNA endonuclease-targeted CRISPR trans reporter [298]<br>▪ **Cas12aVDet**: Cas12a-based visual detection[328]<br>▪ **POIROT**: Photo-initiated CRISPR–Cas12a system for robust one-pot testing[98]

Cas protein involved	Mechanism	Advantages / drawbacks	Detection platforms
	non-specifically cleaves ssDNA (trans-acting)[298]		▪ **HOLMES**: One-hour low-cost multipurpose highly efficient system[327]<br>▪ **SCAN**: Solid-state CRISPR/Cas12a-assisted nanopores [330]<br>▪ **E-CRISPR**: CRISPR/Cas12a based electrochemical biosensor [331]<br>▪ **apta-HCR-CRISPR**: Aptamer-hybridization chain reaction-CRISPR[332]<br>▪ **CaT-SMelor**: CRISPR/Cas12a- and aTF (allosteric transcription factors)-mediated small molecule detector[333]<br>▪ fDNA-regulated CRISPR-Cas sensor[99]<br>▪ **UCAD**: Ultrasensitive CRISPR/Cas12a-based antibody detection assay[334]
Cas12b	▪ Recognizes the PAM sequence of 5′-TTN as Cas12a<br>▪ Upon cleaving DNA, it produces sticky ends and subsequently activates collateral cleavage	▪ *Advantages*:<br>- smaller size of Cas12b makes it more suitable for clinical use<br>- can react over a wide temperature range and pH range with lower off-target effects[337,338]	▪ **HOLMESv2**: One-hour low-cost multipurpose highly efficient system v2[339]<br>▪ **CDetection**: Cas12b-mediated DNA detection[342]
Cas12c	▪ Cas12c protein functions exclusively as an RNA-induced DNA-binding enzyme while lacking DNase activity<br>▪ Three types of Cas12c reported so far: Cas12c1, Cas12c2, and OspCas12c<br>▪ PAM of Cas12c1 and OspCas12c is 5′TG, while that of Cas12c2 is 5′TN[343]	▪ *Advantages*:<br>- broad recognition range different from other Cas12 systems<br>- Cas12c1 exhibited ssDNA trans-cleavage activity equivalent to the Cas12a system<br>- higher sensitivity and specificity for nucleic acid detection compared to Cas12a	▪ **Cas12c-DETECTOR:** Cas12c-based nucleic acid detection platform[100]
Cas13	▪ Cas13 system, consists of a single effector, (Cas13) complexed with crRNA without the need for tracrRNA<br>▪ Cas13 incorporates nucleotide-binding higher eukaryotes and prokaryotes nucleotide-binding ribonuclease domains enabling it to process precursor crRNA, cleave target RNA, and degrade nonspecific bystander RNA	▪ *Advantages*:<br>- targets RNA rather than DNA<br>- besides cleaving specific RNA, Cas13 can target arbitrary RNA (collateral cleavage) when it is activated providing a new solution for nucleic acid detection<br>- the Cas13 system, guided by crRNA, stands out for its ability to target RNA sequences without the need for PAMs, thereby broadening its range of targetability[52, 101]	▪ **SHERLOCK**: Specific high-sensitivity enzymatic reporter unlocking[348]<br>▪ **SHERLOCKv2**[299]<br>▪ **HUDSON**: Heating unextracted diagnostic samples to obliterate nucleases<br>▪ **CARMEN**: Combinatorial arrayed reactions for multiplexed evaluation of nucleic acids[350]<br>▪ **DESCS**: Dual methylation-sensitive restriction endonucleases coupling

Cas protein involved	Mechanism	Advantages / drawbacks	Detection platforms
			with an RPA-assisted CRISPR/Cas13a system[352]<br>▪ **CrisprZyme**: CRISPR/Cas-based reaction with a nanozyme-linked immunosorbent assay[351]<br>▪ **PADLOCK-CRISPR**: Picoinjection aided digital reaction unLOCKing [353]<br>▪ **APC-Cas**: Allosteric probe initiation catalysis and CRISPR/Cas13a[355]<br>▪ **SPRINT**-SHERLOCK-based profiling of *in vitro* transcription[354]<br>▪ **CLISA**: CRISPR/Cas13a signal amplification linked immunosorbent assay[358]
Cas14 (Cas12f)	▪ Cas14 binds to its target ssDNA/dsDNA and activates a collateral cleavage mechanism similar to Cas12 and Cas13	▪ ***Advantages***:<br>- detect and cleave ssDNA without requiring the presence of a PAM[360]<br>- they are significantly more compact (400 to 700 amino acids) than Cas12a (about the half size)<br>- Cas14, unlike Cas12, has lower tolerance to nucleotide mismatches between sgRNA and the target template, which greatly reduces on-target activity of Cas14 allowing use of Cas14 to detect SNPs in DNA[102]	▪ **HARRY**: Highly sensitive aptamer-regulated Cas14 R-loop for bioanalysis[363]
Cas3	▪ Operates with a CRISPR-associated complex called Cascade[364]<br>▪ Cascade complex first recognizes the target DNA based on sequence complementarity between the target and the crRNA<br>▪ Upon recognition, Cas3 is recruited to the site and degrades the target DNA in a helicase-nuclease fashion[365]	▪ ***Advantage***:<br>- can cleave long stretches of the target DNA, unlike Cas9 or Cas12, which only create DSBs<br>▪ ***Drawback***:<br>- requires multiple Cas proteins	▪ **CONAN**: Cas3-operated nucleic acid detection[297]

Table S5. Summary of CRISPR/Cas9-mediated detection platform.

Method	Target	Amplification	Time	Readout	Sensitivity	Application
**Cas9**						
NASBACC[103]	RNA	NASBAtoehold	2–6 h	Colorimetry	fmol/L	Zika viruses
Cas-EXPAR[104]	DNA/ RNA	EXPAR	≤1h	Fluorescence	Sub-amol/L	DNA methylation and *Listeria monocytogenes* RNA
FLASH-NGS[105]	DNA	PCR	~2h	Sequencing	Sub-amol/L	Drug-resistant microbes
FELUDA[106]	dsDNA	LFD PCR/RPA	<1h	Fluorescence	~110*10-15mol	SARS-CoV-2
**dCas9**						
CASLFA[107]	DNA	RAP/PCR	≤1h	Lateral flow	150–200 copies/ reaction	Microbes
dCas9-DNA-FISH[108]	DNA	No amplification	30 min	Fluorescence	10 CFU/ml	MRSA
Paired dCas9 (PC)[109]	DNA	PCR	10 min after PCR	Fluorescence	Single copy/ reaction	Mycobacterium tuberculosis
RCasFISH[110]	RNA	No amplification	<1.5 h	Fluorescence	10 copies	Human epidermal growth factor receptor 2 (HER2)
**nCas9**						
CRISDA[111]	DNA	KF-mediated amplification	2–3 h	Fluorescence	amol/L	Detection of breast cancer-associated SNPs genotyping
Cas9nAR[112]	DNA	KF-mediated amplification	<1h	Fluorescence	2 copies/reaction	SNP

Table S6. Summary of CRISPR/Cas12-mediated detection platform.

Method	Target	Amplification	Time	Readout	Sensitivity	Application
**Cas12a**						
DETECTR[113]	DNA	LAMP/RPA	~70 min	Fluorescence	amol/L	HPV
Cas12aVDet[114]	DNA	RPA	<30 min	Fluorescence	10aM	Mycoplasma
POIROT[115]	RNA/DNA	RPA	20 min	Fluorescence	1 copy/μL	SARS-CoV-2, HPV-16 and HPV-18
HOLMES[116]	RNA/DNA	(RT-)PCR	~1h	Fluorescence	amol/L	Methylation of cancer gene
SCAN[117]	DNA	RPA	~1h	Nanopore	10 nM	HIV
E-CRISPR[118]	DNA	non-enzymatic amplified	~1.5 h	Electrochemical biosensor	50 pM for nucleic acid detection and 0.2 nM for protein detection	Cancer marker
apta-HCR-CRISPR[119]	Protein	HCR	>1h	Fluorescence	102 particles/μL	TEV protein markers
CaT-SMelor[120]	DNA	No amplification	~20 min	Fluorescence	nmol/L	Uric acid and p-hydroxybenzoic acid among their structurally similar analogues
fDNA-regulated CRISPR-Cas sensor[121]	DNA	No amplification	~15 min	Fluorescence	≈ 0.21 μM	ATP and Na+ in human plasma
UCAD[122]	Antibody	RPA	40 min	Fluorescence	10aM	SARS-CoV-2 antibodies in blood samples
**Cas12b**						
HOLMESv2[123]	RNA/DNA	(RT)-LAMP	~1h	Fluorescence	10–5 nM	DNA methylation, SNP, RNA virus
CDetection[124]	DNA	RPA	~3h	Fluorescence	0.1aM	SNP in cancer
**Cas12c**						

Cas12c-DETECTR[125]	RNA/DNA	(RT)- RPA	-	Fluorescence/ Lateral flow	0.23 pM	SNP, human and plant pathogens

Table S7. Summary of CRISPR/Cas13-mediated detection platform.

Method	Target	Amplification	Time	Readout	Sensitivity	Application
SHERLOCK[126]	RNA/ DNA	RPA/RT-RPA	2–5 h	Fluorescence	amol/L	Infectious agents, SNP in cancer
SHERLOCKv2[127]	RNA/ DNA	RPA	0.5–3 h	Fluorescence, Colorimetry Lateral flow	zmol/L	Multiple targets
HUDSON + SHERLOCK[128]	RNA/ DNA	RPA	2 h	Fluorescence, Colorimetry Lateral flow	amol/L	Zika and Dengue viruses
CARMEN[129]	RNA	PCR or RPA	-	Fluorescence	aM	Pandemic pathogen screening
DESCS 86.4[130]	DNA	RPA	~30 min	Fluorescence	aM	Site-specific DNA methylation
CrisprZyme[131]	RNA	No amplification	6 h	Colorimetric analysis	7.88 ± 3.21 pM	Myocardial infarction and prostate cancer
PADLOCK[132]	RNA/ DNA	ddRPA	30 min	Fluorescence	9500 cp/μl	Nucleic acid quantification
APC-Cas[133]	Cell	RPA	~2h	Fluorescence	1 CFU/mL	Salmonella Enteritidis
SPRINT[134]	RNA	No amplification	35 min	Fluorescence	pM	Cofactors, nucleotides, metabolites of amino acids, tetracycline, and monatomic ions
CLISA[135]	IL-6, VEGF	T7 transcription	30 min	Fluorescence	2.29 fM, 0.81 fM	Inflammatory factor (human IL-6), and a tumor marker (human vascular endothelial growth factor (human VEGF),

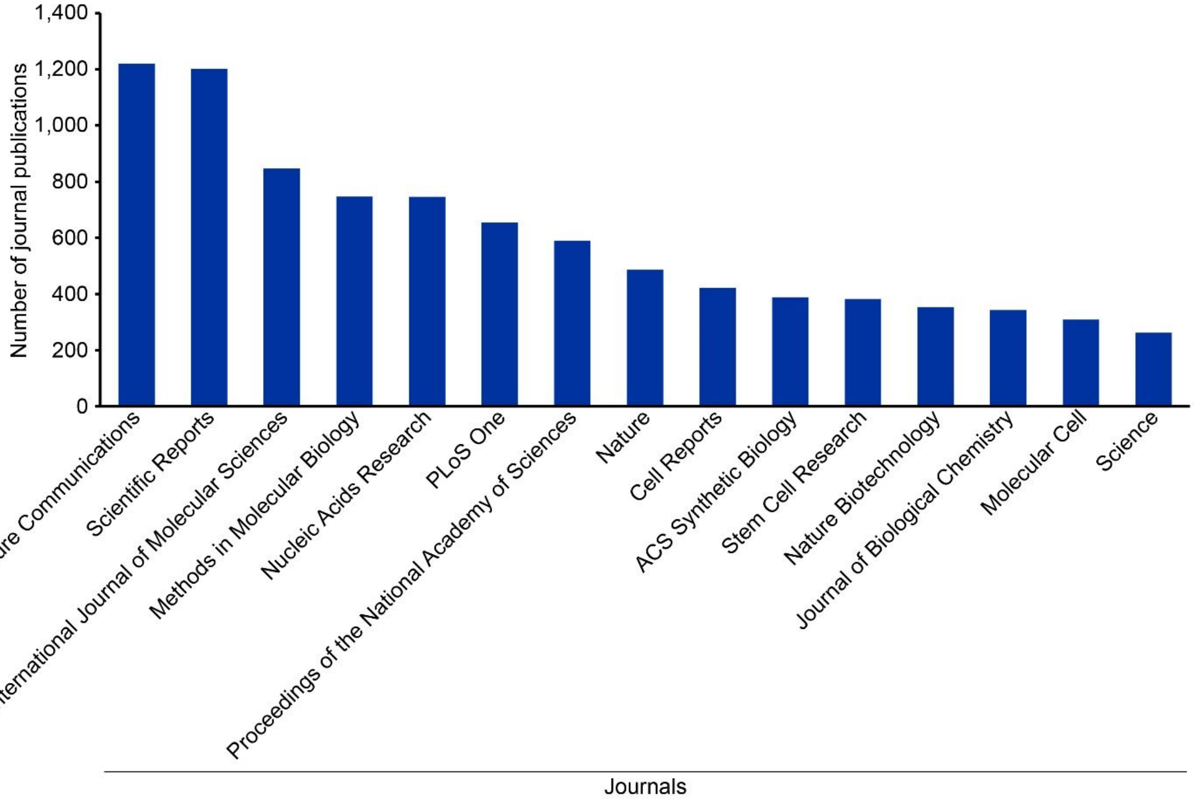

Figure S6. Leading journals in the field of CRISPR in terms of volume of research published. Data includes journal publications from the CAS Content Collection for the period 1995-2024. Note that data for 2024 is incomplete due to time of data extraction and encompasses data for January to June.

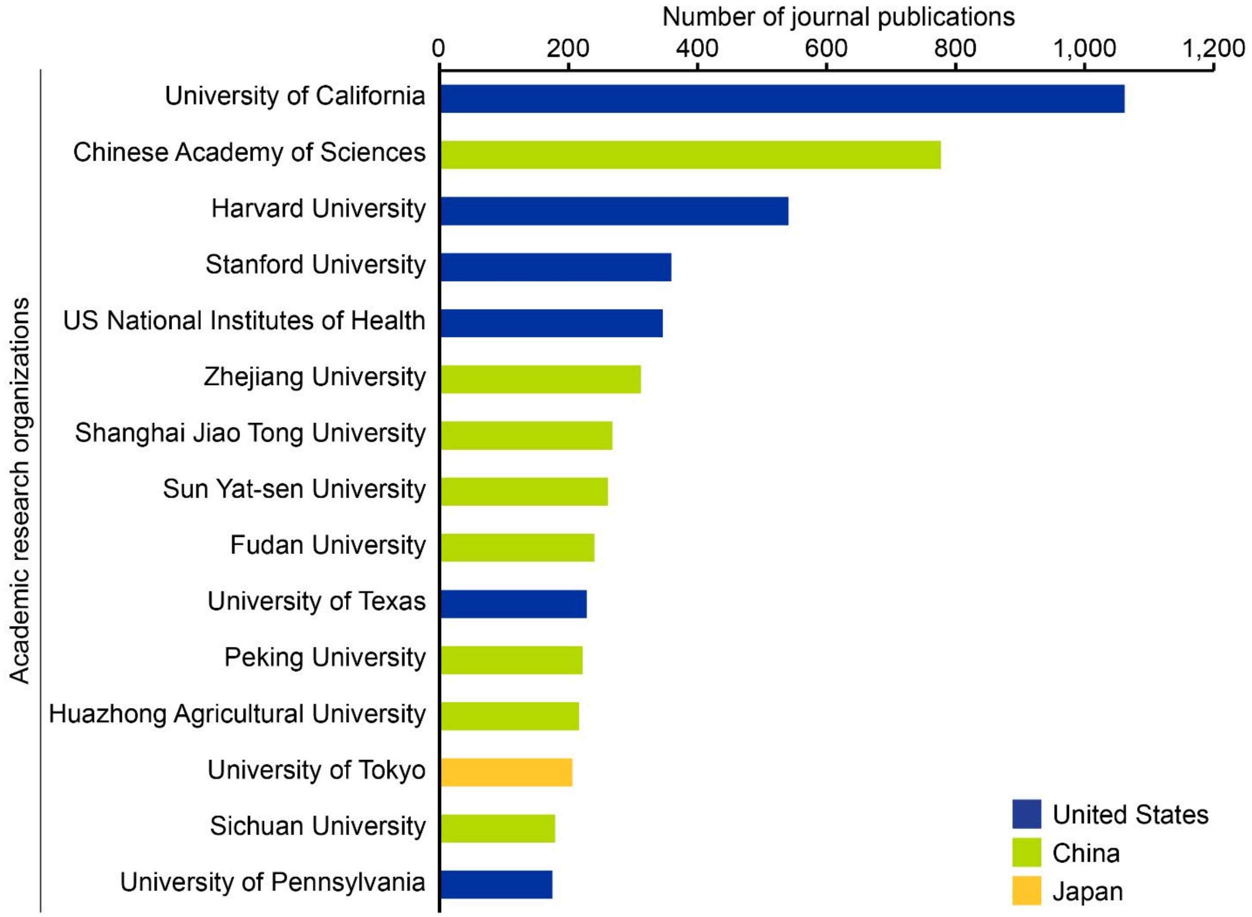

Figure S7. Leading academic research organizations in the field of CRISPR documents in terms of volume of research published. The bars have been color coded to indicate geographical location. Data includes journal from the CAS Content Collection for the period 1995-2024. Note that data for 2024 is incomplete due to time of data extraction and encompasses data for January to June.

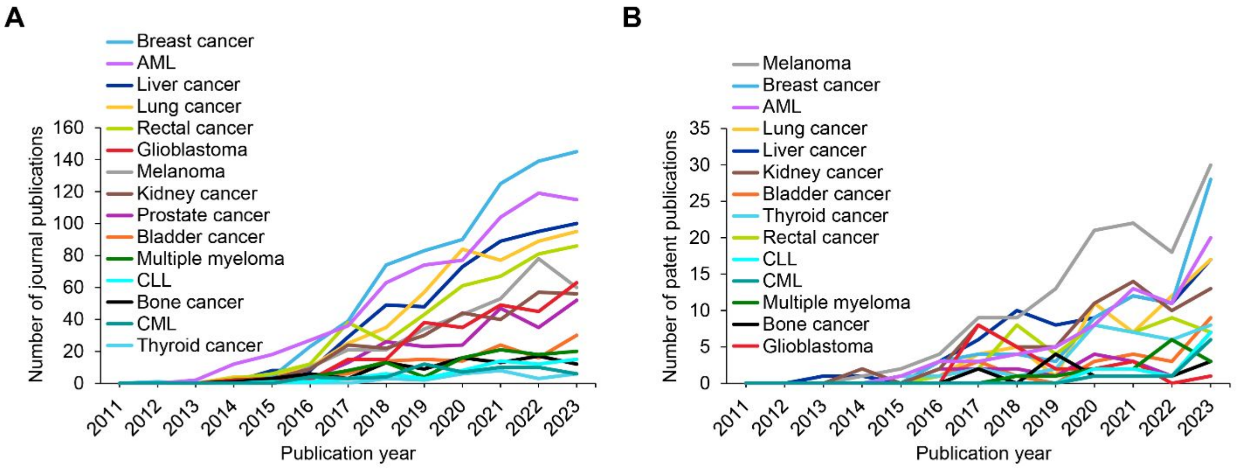

Figure S8. Time trends for CRISPR (**A**) journal publications and (**B**) patents co-occurring with various subtypes of cancer. Data includes journal and patent publications from the CAS Content Collection. Abbreviations used: AML, acute myeloid leukemia; CLL, chronic lymphocytic leukemia; CML, chronic myeloid leukemia.

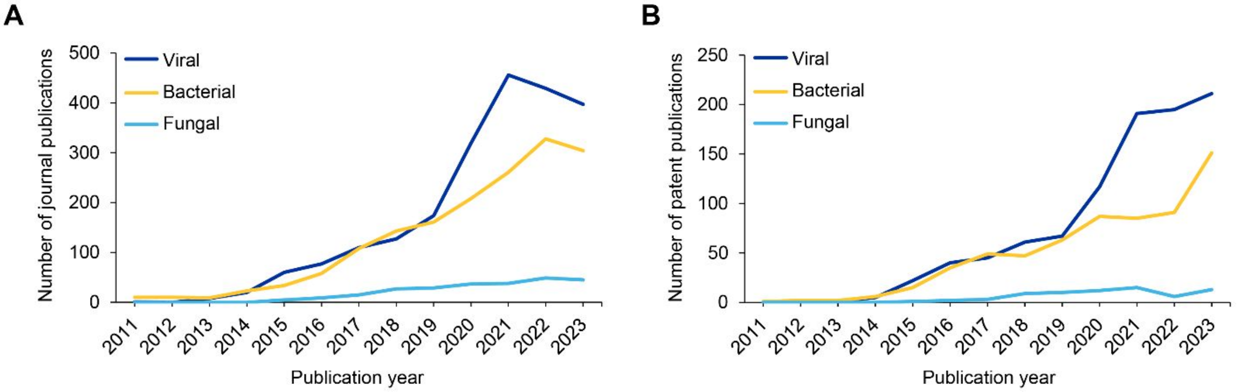

Figure S9. Time trends for CRISPR (**A**) journal publications and (**B**) patents co-occurring with three major types of infectious diseases (viral, bacterial and fungal). Data includes journal and patent publications from the CAS Content Collection.

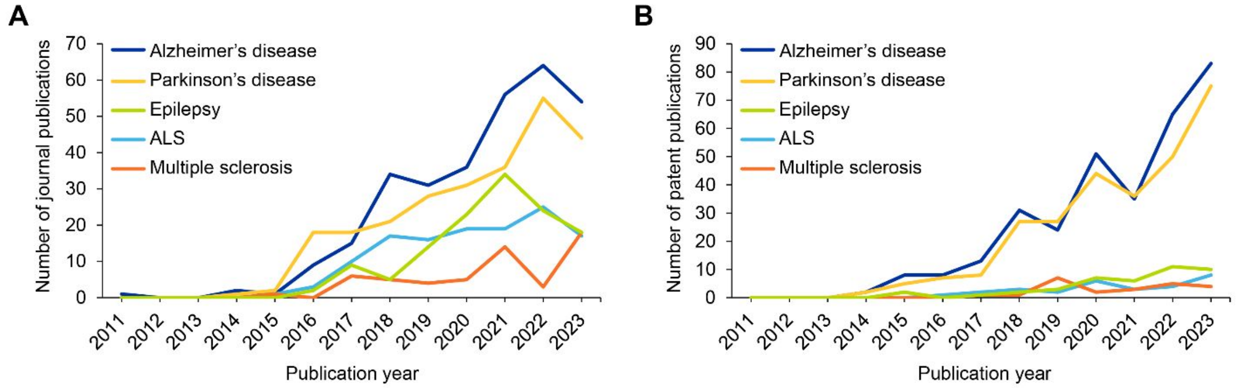

Figure S10. Time trends for CRISPR (**A**) journal publications and (**B**) patents co-occurring with nervous system disorders (Alzheimer’s, Parkinson’s, epilepsy, ALS and multiple sclerosis). Data includes journal and patent publications from the CAS Content Collection. Abbreviation used: ALS, amyotrophic lateral sclerosis.

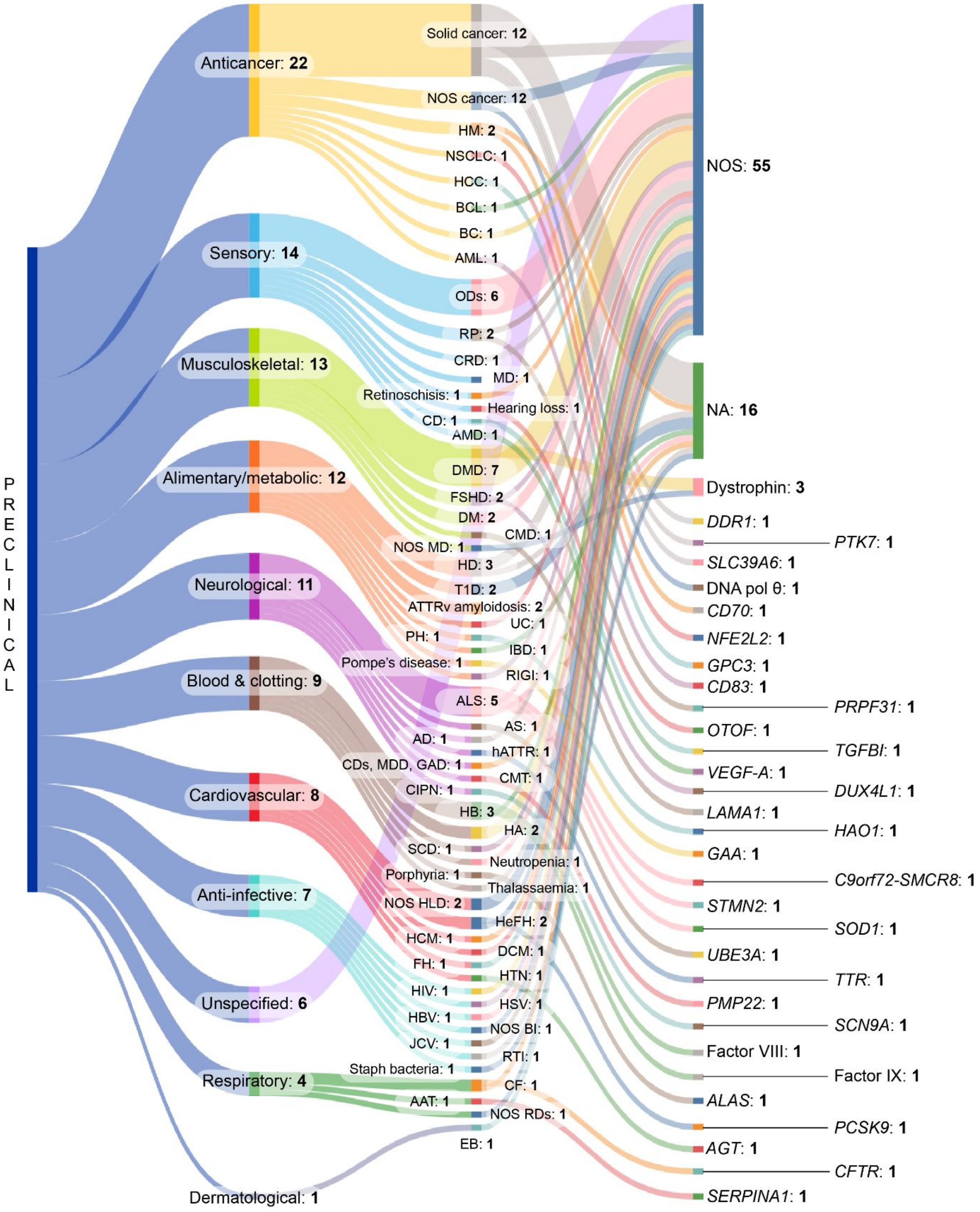

Figure S11. Distribution of CRISPR-based therapeutics in the preclinical stage of development across disease groups (2nd column from the left), individual diseases (3rd column from the left), and their biological targets (4th column from the left). Data retrieved from Pharmaproject Citeline Clinical Intelligence in June 2024. The names of the diseases and their targets are abbreviated here as: AAT, alpha-1 antitrypsin

deficiency; AD, Alzheimer's disease; *AGT*, angiotensinogen; *ALAS*, 5'-aminolevulinate synthase 1; ALS, amyotrophic lateral sclerosis; AMD, age-related macular degeneration; AML, acute myeloid leukemia; AS, Angelman syndrome; BC, breast cancer; BCL, B-cell lymphoma; *C9orf72-SMCR8, C9orf72-SMCR8 complex subunit*; CD, corneal dystrophy; CF, cystic fibrosis; *CFTR, cystic fibrosis transmembrane conductance regulator*; CIPN, chemotherapy-induced peripheral neuropathy; CMD, congenital muscular dystrophy; CMT, Charcot–Marie–Tooth disease; CRD, cone-rod dystrophy; DCM, dilated cardiomyopathy; *DDR1, discoidin domain receptor tyrosine kinase 1*; DM, myotonic dystrophy; DMD, Duchenne muscular dystrophy; *DNA pol θ, DNA polymerase theta*; *DUX4L1, double homeobox 4 like*; EB, epidermolysis bullosa; FH, familial hypercholesterolemia; FSHD, facioscapulohumeral muscular dystrophy; *GAA, glucosidase alpha acid*; GAD, generalized anxiety disorder; *GPC3, glypican 3*; HA, haemophilia A; *HAO1, hydroxyacid oxidase 1*; hATTR, hereditary transthyretin amyloidosis; HB, haemophilia B; HBV, hepatitis-B virus; HCC, hepatocellular carcinoma; HCM, hypertrophic cardiomyopathy; HD, hepatic dysfunction; HeFH, heterozygous familial hypercholesterolaemia; HIV, human immunodeficiency virus; HM, hematological malignancies; HSV, herpes simplex virus; HTN, hypertension; IBD, inflammatory bowel disease; JCV, John Cunningham virus; *LAMA1*, laminin subunit alpha 1; MD, macular dystrophy; MD, macular dystrophy; MDD, major depressive disorder; NA, not applicable; *NFE2L2, nuclear factor erythroid 2 like 2*; NOS, not specified; NOS BI, unspecified bacterial Infection; NOS HLD, unspecified hyperlipidaemia; NOS MD, unspecified muscular dystrophy; NOS RD, unspecified respiratory disease; NSCLC, non-small cell lung cancer; OD, ocular disorder; *OTOF, otoferlin*; *PCSK9, proprotein convertase subtilisin/kexin type 9*; PH, primary hyperoxaluria; *PMP22*, peripheral myelin protein 22; *PRPF31*, pre-mRNA processing factor 31; *PTK7,* protein tyrosine kinase 7; RIGI, radiation-induced gastric injury; RP, retinitis pigmentosa; RTI, respiratory tract infection; SCD, sickle cell anaemia; *SCN9A*, sodium voltage-gated channel alpha subunit 9; *SERPINA1*, serpin family A member 1; *SLC39A6*, solute carrier family 39 member 6; *SOD1*, superoxide dismutase 1; *STMN2*, stathmin 2; T1D, type 1 diabetes; *TGFBI*, transforming growth factor beta induced; *TTR*, transthyretin; *UBE3A*, ubiquitin protein ligase E3A; UC, ulcerative colitis; *VEGF-A*, vascular endothelial growth factor A.

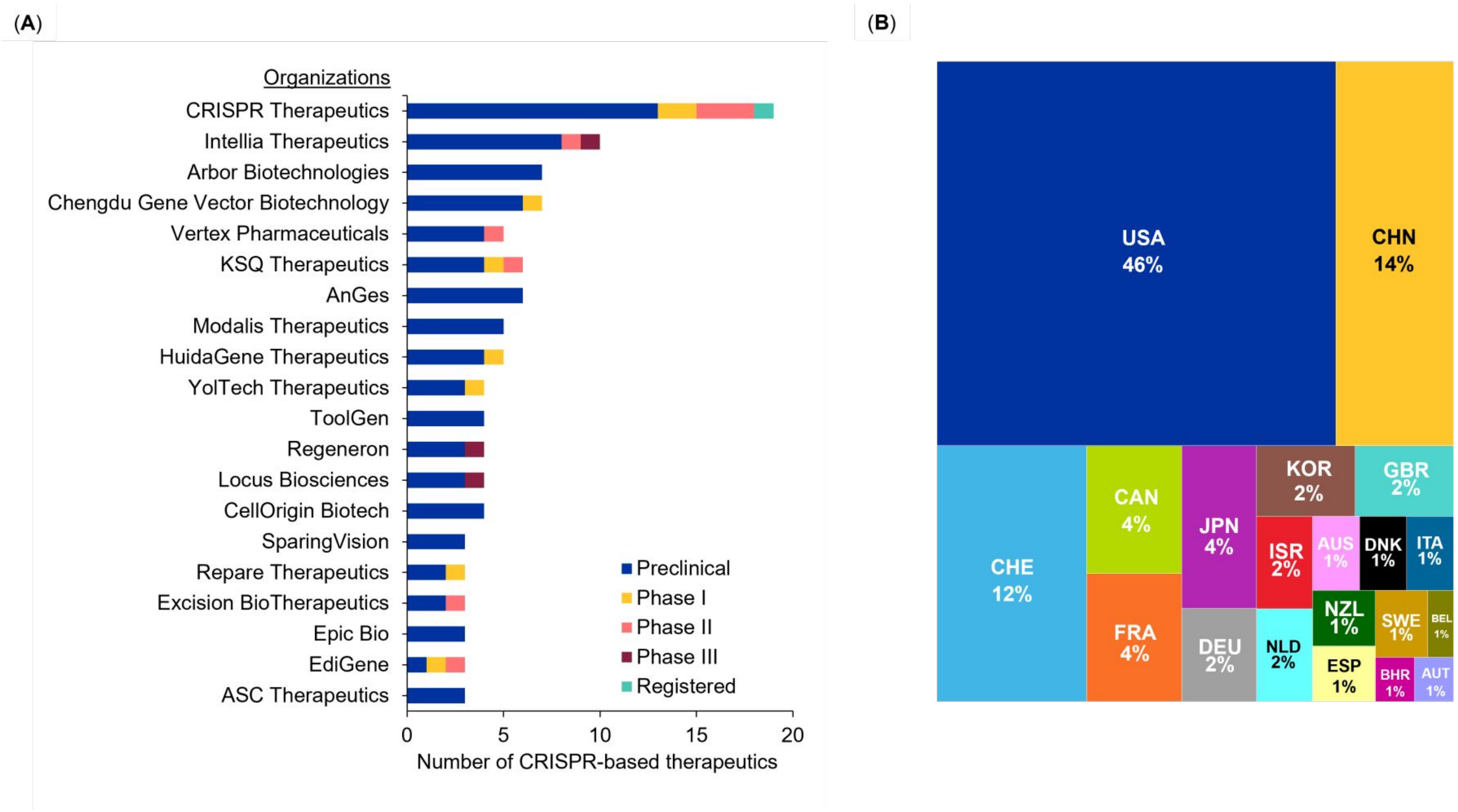

Figure S12. (A) Major organizations conducting preclinical and clinical research on CRISPR therapy. Stacked bars represent the number of drugs in various stages of development for each company. **(B)** Country/region-wise distribution of the number of CRISPR-based therapeutics (in percentage) in preclinical and clinical research of drug development. Data was taken from Pharmaproject Citeline Clinical Intelligence in June 2024.

Standard three letter codes used as abbreviations for country/region names: USA: United States, CHN: China, CHE: Switzerland, CAN: Canada, FRA: France, JPN: Japan, DEU: Germany, KOR: South Korea, GBR: United Kingdom, ISR: Israel, NLD: Netherland, AUS: Australia, DNK: Denmark, ITA: Italy, NZL: New Zealand, SWE: Sweden, BEL: Belgium, ESP: Spain, BHR: Beharan, AUT: Austria.

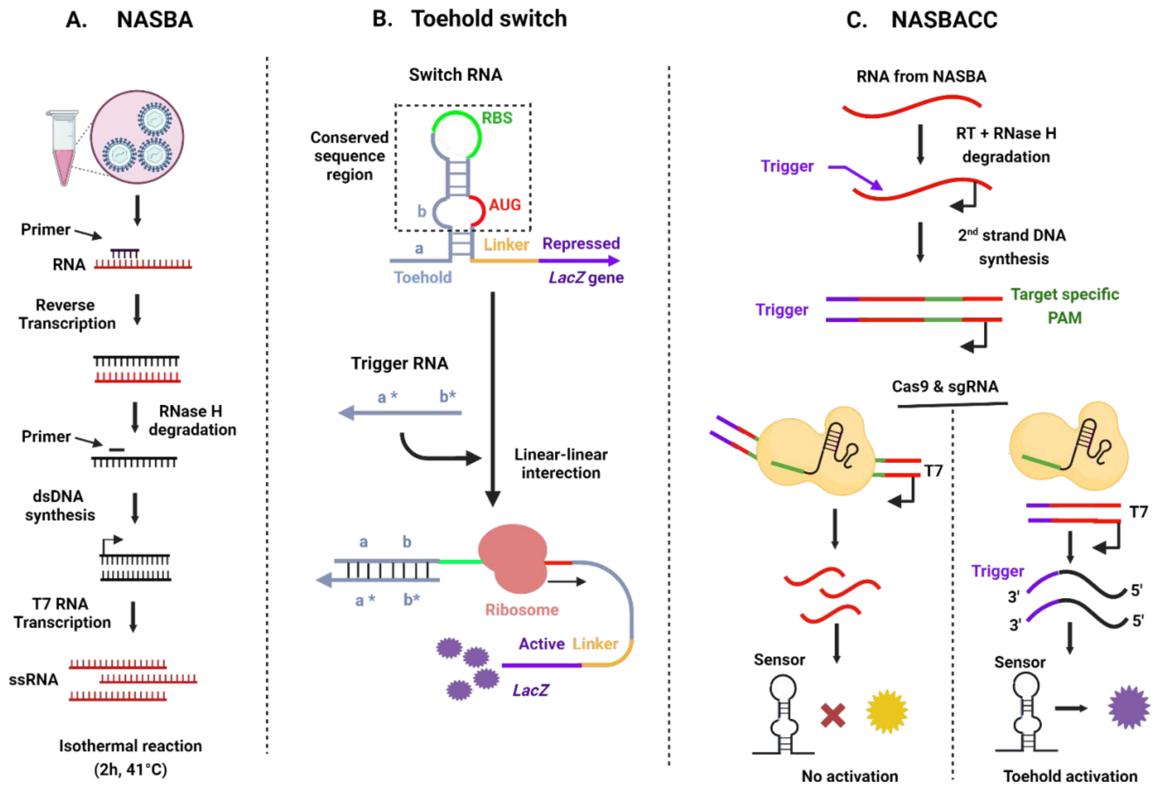

Figure S13. Schematics of CRISPR-Cas9-based CRISPR-diagnostic methods: (**A**) The amplification process of NASBA (nucleic acid sequence-based amplification)-mediated RNA amplification begins with reverse transcription of a target RNA and generates a dsDNA product. T7-mediated transcription of the DNA template then creates copies of the target RNA sequence. Importantly, each new target RNA can be detected by the toehold switch sensors and also serve as starting material for further amplification cycles. (**B**) Toehold switch systems are composed of two RNA strands referred to as the switch and trigger. The switch RNA contains the coding sequence of the gene, ribosome binding site (RBS) and a start codon that is followed by a common 21 nt linker sequence coding for low-molecular-weight amino acids added to the N terminus of the gene of interest. A single-stranded toehold sequence at the 5′ end of the hairpin module provides the initial binding site for the trigger RNA strand. This trigger molecule contains an extended single-stranded region that completes a branch migration process with the hairpin to expose the RBS and start codon, thereby initiating translation of the gene of interest. (**C**) NASBACC exploits the ability of Cas9 to selectively cleave DNA only in the presence of an NGG PAM. In the presence of PAM and gRNA target site, the dsDNA that is synthesized as part of the NASBA reaction undergoes Cas9-mediated cleavage, resulting in a truncated RNA product that is unable to activate the sensor toehold switch. In the absence of the PAM sequence, the full-length RNA product containing the sensor trigger sequence is generated, allowing for sensor activation. Trigger RNA is only amplified from DNA that is not cut by Cas9, thereby allowing for strain-specific detection using toehold sensor. These schematics have been created using www.BioRender.com.

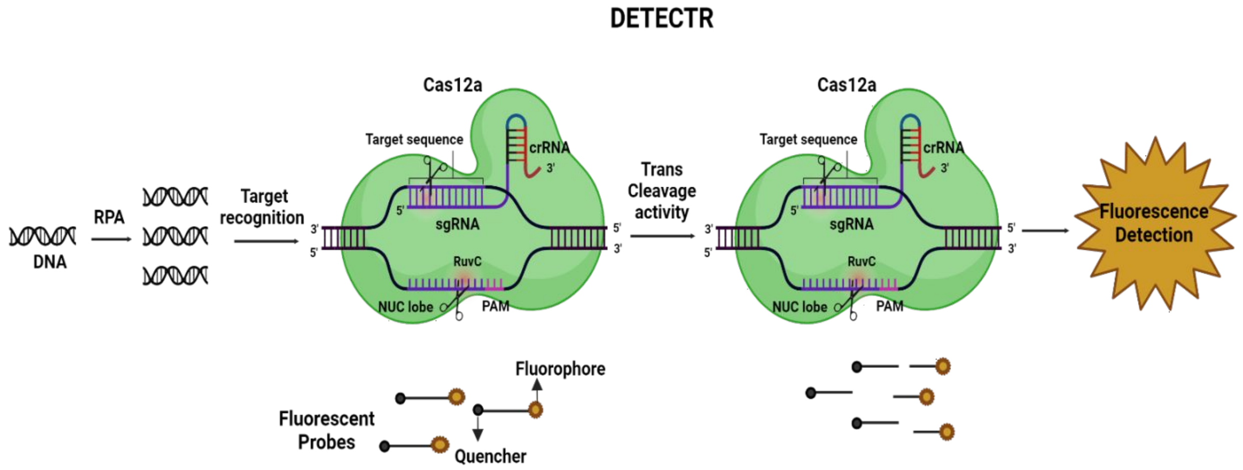

Figure S14. Schematic presentation of CRISPR-Cas12a based DETECTR diagnostic platform. The DNA molecule is amplified using isothermal amplification followed by the addition of the Cas12a mix with sgRNA and fluorescent probes. Cas12a recognizes the target DNA and cleaves fluorescent probes by means of collateral activity to produce a fluorescent signal. Created using www.BioRender.com.

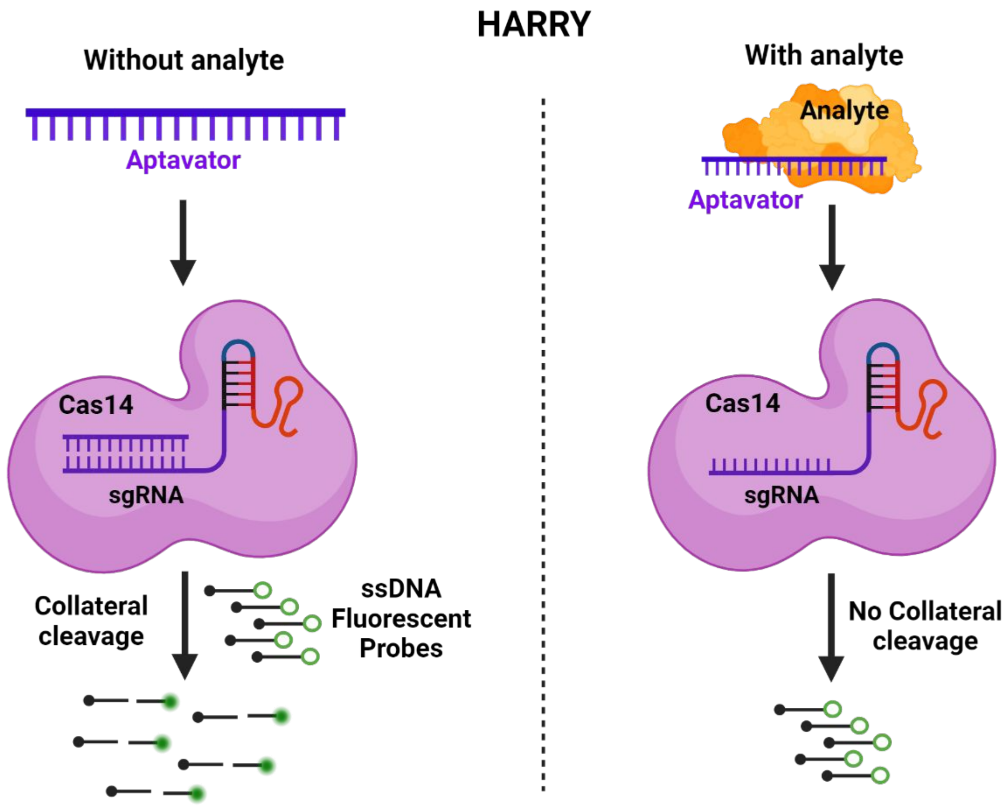

Figure S15. Illustration of the principle of HARRY (highly sensitive aptamer-regulated Cas14 R-loop for bioanalysis). A DNA strand named "Aptavator" functions as both the aptamer and the activator of Cas14a. In the absence of targets (analyte) (left panel), Cas14a-sgRNA ribonucleoprotein recognizes the Aptavator and forms a stable R-loop structure to cleave nearby ssDNA probe resulting in fluorescence enhancement. In the presence of targets (analyte) (right panel), the Aptavator is tightly bound to the targets because of its high affinity towards the targets, minimizing hybridization with the sgRNA complexed in Cas14a, forming the R-loop and inhibiting the activation of Cas14a. Consequently, probe remains intact maintaining the low fluorescence. The Illustration has been created by using www.BioRender.com.

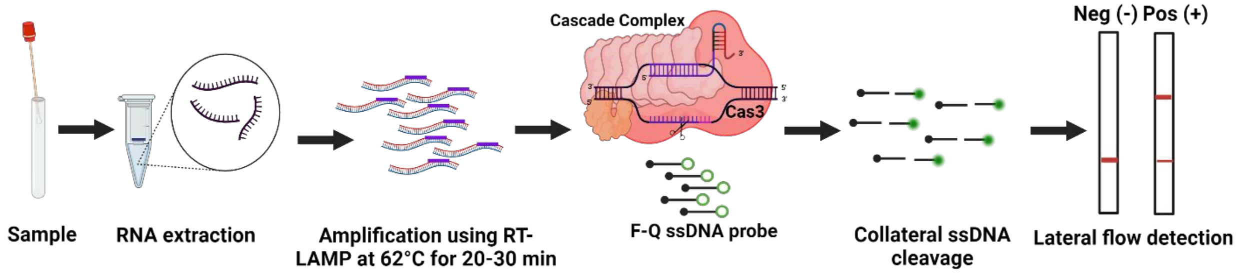

Figure S16. Schematic representation of the Cas3-Operated Nucleic Acid detectioN (CONAN) lateral flow assay: An *in vitro* nucleic acid-detection platform. The extracted RNA is amplified using RT-LAMP. The amplified product is incubated with CRISPR/Cas3 complex containing Cas3, Cas5, Cas6, Cas7, Cas8, and Cas11 proteins along with crRNA and fluorophore quencher (F-Q)-labeled ssDNA probe for 10 min at 37 °C. CRISPR/Cas3 trans cleaves nonspecific ssDNA after target-specific dsDNA cleavage which recognizes the PAM. Resulting fluorescence is detected through lateral flow assay. The abundant reporter accumulates anti-FITC antibody-gold nanoparticle conjugates at the first line (negative) on the strip, whereas cleavage of the reporter would reduce accumulation on the first line and result in signal on the second line (positive) with <2 min of flow at RT. The Illustration has been created by using www.BioRender.com.

References

(1) Ishino, Y.; Shinagawa, H.; Makino, K.; Amemura, M.; Nakata, A. Nucleotide Sequence of The IAP Gene, Responsible for Alkaline Phosphatase Isozyme Conversion in *Escherichia coli*, and Identification of the Gene Product. *J. Bacteriol.* **1987**, *169* (12), 5429-5433. DOI: 10.1128/jb.169.12.5429-5433.1987.
(2) Jansen, R.; Embden, J. D.; Gaastra, W.; Schouls, L. M. Identification of Genes that are Associated with DNA Repeats in Prokaryotes. *Mol. Microbiol.* **2002**, *43* (6), 1565-1575. DOI: 10.1046/j.1365-2958.2002.02839.x.
(3) Ishino, Y.; Krupovic, M.; Forterre, P. History of CRISPR-Cas from Encounter with a Mysterious Repeated Sequence to Genome Editing Technology. *J. Bacteriol.* **2018**, *200* (7), e00580-00517/00581-e00580-00517/00517. DOI: 10.1128/JB.00580-17.
(4) Sorek, R.; Kunin, V.; Hugenholtz, P. CRISPR--A Widespread System that Provides Acquired Resistance Against Phages in Bacteria and Archaea. *Nat. Rev. Microbiol.* **2008**, *6* (3), 181-186. DOI: 10.1038/nrmicro1793.
(5) Hille, F.; Charpentier, E. CRISPR-Cas: Biology, Mechanisms and Relevance. *Philos. Trans. R. Soc. Lond. B Biol. Sci.* **2016**, *371* (1707). DOI: 10.1098/rstb.2015.0496.
(6) Ibrahim, A. U.; Özsoz, M.; Saeed, Z.; Tirah, G.; Gideon, O. Genome Engineering Using the CRISPR Cas9 System. *J. Biomed. Pharm. Sci.* **2019**, *2*, 127.
(7) Mojica, F. J.; Diez-Villasenor, C.; Garcia-Martinez, J.; Soria, E. Intervening Sequences of Regularly Spaced Prokaryotic Repeats Derive from Foreign Genetic Elements. *J. Mol. Evol.* **2005**, *60* (2), 174-182. DOI: 10.1007/s00239-004-0046-3.
(8) Marraffini, L. A. CRISPR-Cas Immunity in Prokaryotes. *Nature* **2015**, *526* (7571), 55-61. DOI: 10.1038/nature15386.
(9) van der Oost, J.; Jore, M. M.; Westra, E. R.; Lundgren, M.; Brouns, S. J. CRISPR-based Adaptive and Heritable Immunity in Prokaryotes. *Trends Biochem. Sci.* **2009**, *34* (8), 401-407. DOI: 10.1016/j.tibs.2009.05.002.
(10) Jackson, S. A.; McKenzie, R. E.; Fagerlund, R. D.; Kieper, S. N.; Fineran, P. C.; Brouns, S. J. CRISPR-Cas: Adapting to change. *Science* **2017**, *356* (6333). DOI: 10.1126/science.aal5056.
(11) Barrangou, R.; Fremaux, C.; Deveau, H.; Richards, M.; Boyaval, P.; Moineau, S.; Romero, D. A.; Horvath, P. CRISPR Provides Acquired Resistance Against Viruses in Prokaryotes. *Science* **2007**, *315* (5819), 1709-1712. DOI: 10.1126/science.1138140.
(12) Haft, D. H.; Selengut, J.; Mongodin, E. F.; Nelson, K. E. A guild of 45 CRISPR-associated (Cas) protein families and multiple CRISPR/Cas subtypes exist in prokaryotic genomes. *PLoS Comput Biol* **2005**, *1* (6), e60. DOI: 10.1371/journal.pcbi.0010060 From NLM Medline.
(13) Makarova, K. S.; Grishin, N. V.; Shabalina, S. A.; Wolf, Y. I.; Koonin, E. V. A Putative RNA-interference-based Immune System in Prokaryotes: Computational Analysis of the Predicted Enzymatic Machinery, Functional Analogies with Eukaryotic RNAi, and Hypothetical Mechanisms of Action. *Biol. Direct.* **2006**, *1*, 7. DOI: 10.1186/1745-6150-1-7.
(14) Gleditzsch, D.; Pausch, P.; Muller-Esparza, H.; Ozcan, A.; Guo, X.; Bange, G.; Randau, L. PAM Identification by CRISPR-Cas Effector Complexes: Diversified Mechanisms and Structures. *RNA Biol.* **2019**, *16* (4), 504-517. DOI: 10.1080/15476286.2018.1504546.
(15) *Press release: The Nobel Prize in Chemistry 2020*. 2020. https://www.nobelprize.org/prizes/chemistry/2020/press-release/ (accessed 2024 December 31).
(16) Jinek, M.; Chylinski, K.; Fonfara, I.; Hauer, M.; Doudna, J. A.; Charpentier, E. A Programmable Dual-RNA-guided DNA Endonuclease in Adaptive Bacterial Immunity. *Science* **2012**, *337* (6096), 816-821. DOI: 10.1126/science.1225829.
(17) Jiang, W.; Bikard, D.; Cox, D.; Zhang, F.; Marraffini, L. A. RNA-guided Editing of Bacterial Genomes Using CRISPR-Cas Systems. *Nat. Biotechnol.* **2013**, *31* (3), 233-239. DOI: 10.1038/nbt.2508.

(18) Shao, M.; Xu, T. R.; Chen, C. S. The Big Bang of Genome Editing Technology: Development and Application of the CRISPR/Cas9 System in Disease Animal Models. *Dongwuxue Yanjiu* **2016**, *37* (4), 191-204. DOI: 10.13918/j.issn.2095-8137.2016.4.191.
(19) Ceasar, S. A.; Rajan, V.; Prykhozhij, S. V.; Berman, J. N.; Ignacimuthu, S. Insert, Remove or Replace: A Highly Advanced Genome Editing System Using CRISPR/Cas9. *Biochim. Biophys. Acta* **2016**, *1863* (9), 2333-2344. DOI: 10.1016/j.bbamcr.2016.06.009.
(20) Mei, Y.; Wang, Y.; Chen, H.; Sun, Z. S.; Ju, X. D. Recent Progress in CRISPR/Cas9 Technology. *J. Genet. Genomics* **2016**, *43* (2), 63-75. DOI: 10.1016/j.jgg.2016.01.001.
(21) Jiang, F.; Doudna, J. A. CRISPR-Cas9 Structures and Mechanisms. *Annu. Rev. Biophys.* **2017**, *46*, 505-529. DOI: 10.1146/annurev-biophys-062215-010822.
(22) Liu, M.; Rehman, S.; Tang, X.; Gu, K.; Fan, Q.; Chen, D.; Ma, W. Methodologies for Improving HDR Efficiency. *Front. Genet.* **2018**, *9*, 691. DOI: 10.3389/fgene.2018.00691.
(23) Yang, H.; Ren, S.; Yu, S.; Pan, H.; Li, T.; Ge, S.; Zhang, J.; Xia, N. Methods Favoring Homology-Directed Repair Choice in Response to CRISPR/Cas9 Induced-Double Strand Breaks. *Int. J. Mol. Sci.* **2020**, *21* (18), 6461. DOI: 10.3390/ijms21186461.
(24) Makarova, K. S.; Wolf, Y. I.; Iranzo, J.; Shmakov, S. A.; Alkhnbashi, O. S.; Brouns, S. J. J.; Charpentier, E.; Cheng, D.; Haft, D. H.; Horvath, P.; et al. Evolutionary Classification of CRISPR-Cas Systems: A Burst of Class 2 and Derived Variants. *Nat. Rev. Microbiol.* **2020**, *18* (2), 67-83. DOI: 10.1038/s41579-019-0299-x.
(25) Makarova, K. S.; Wolf, Y. I.; Alkhnbashi, O. S.; Costa, F.; Shah, S. A.; Saunders, S. J.; Barrangou, R.; Brouns, S. J.; Charpentier, E.; Haft, D. H.; et al. An Updated Evolutionary Classification of CRISPR-Cas Systems. *Nat. Rev. Microbiol.* **2015**, *13* (11), 722-736. DOI: 10.1038/nrmicro3569.
(26) Yang, J.; Li, X.; He, Q.; Wang, X.; Tang, J.; Wang, T.; Zhang, Y.; Yu, F.; Zhang, S.; Liu, Z.; et al. Structural Basis for the Activity of the Type VII CRISPR-Cas System. *Nature* **2024**, *633* (8029), 465-472. DOI: 10.1038/s41586-024-07815-0.
(27) Koonin, E. V.; Makarova, K. S.; Zhang, F. Diversity, Classification and Evolution of CRISPR-Cas Systems. *Curr. Opin. Microbiol.* **2017**, *37*, 67-78. DOI: 10.1016/j.mib.2017.05.008.
(28) Brouns, S. J.; Jore, M. M.; Lundgren, M.; Westra, E. R.; Slijkhuis, R. J.; Snijders, A. P.; Dickman, M. J.; Makarova, K. S.; Koonin, E. V.; van der Oost, J. Small CRISPR RNAs Guide Antiviral Defense in Prokaryotes. *Science* **2008**, *321* (5891), 960-964. DOI: 10.1126/science.1159689.
(29) Makarova, K. S.; Wolf, Y. I.; Koonin, E. V. Classification and Nomenclature of CRISPR-Cas Systems: Where from Here? *CRISPR J.* **2018**, *1* (5), 325-336. DOI: 10.1089/crispr.2018.0033.
(30) Hayes, R. P.; Xiao, Y.; Ding, F.; van Erp, P. B.; Rajashankar, K.; Bailey, S.; Wiedenheft, B.; Ke, A. Structural Basis for Promiscuous PAM Recognition in Type I-E Cascade from *E. coli*. *Nature* **2016**, *530* (7591), 499-503. DOI: 10.1038/nature16995.
(31) Zhao, H.; Sheng, G.; Wang, J.; Wang, M.; Bunkoczi, G.; Gong, W.; Wei, Z.; Wang, Y. Crystal Structure of the RNA-guided Immune Surveillance Cascade Complex in *Escherichia coli*. *Nature* **2014**, *515* (7525), 147-150. DOI: 10.1038/nature13733.
(32) Kazlauskiene, M.; Kostiuk, G.; Venclovas, C.; Tamulaitis, G.; Siksnys, V. A Cyclic Oligonucleotide Signaling Pathway in Type III CRISPR-Cas Systems. *Science* **2017**, *357* (6351), 605-609. DOI: 10.1126/science.aao0100.
(33) Huang, F.; Zhu, B. The Cyclic Oligoadenylate Signaling Pathway of Type III CRISPR-Cas Systems. *Front. Microbiol.* **2020**, *11*, 602789. DOI: 10.3389/fmicb.2020.602789.
(34) Pinilla-Redondo, R.; Mayo-Munoz, D.; Russel, J.; Garrett, R. A.; Randau, L.; Sorensen, S. J.; Shah, S. A. Type IV CRISPR-Cas Systems are Highly Diverse and Involved in Competition Between Plasmids. *Nucleic Acids Res.* **2020**, *48* (4), 2000-2012. DOI: 10.1093/nar/gkz1197.
(35) Benz, F.; Camara-Wilpert, S.; Russel, J.; Wandera, K. G.; Cepaite, R.; Ares-Arroyo, M.; Gomes-Filho, J. V.; Englert, F.; Kuehn, J. A.; Gloor, S.; et al. Type IV-A3 CRISPR-Cas Systems

Drive Inter-plasmid Conflicts by Acquiring Spacers in Trans. *Cell Host Microbe* **2024**, *32* (6), 875-886 e879. DOI: 10.1016/j.chom.2024.04.016.
(36) Aliaga Goltsman, D. S.; Alexander, L. M.; Lin, J. L.; Fregoso Ocampo, R.; Freeman, B.; Lamothe, R. C.; Perez Rivas, A.; Temoche-Diaz, M. M.; Chadha, S.; Nordenfelt, N.; et al. Compact Cas9d and HEARO Enzymes for Genome Editing Discovered from Uncultivated Microbes. *Nat. Commun.* **2022**, *13* (1), 7602. DOI: 10.1038/s41467-022-35257-7.
(37) Gasiunas, G.; Barrangou, R.; Horvath, P.; Siksnys, V. Cas9-crRNA Ribonucleoprotein Complex Mediates Specific DNA Cleavage for Adaptive Immunity in Bacteria. *Proc. Natl. Acad. Sci. U. S. A.* **2012**, *109* (39), E2579-E2586. DOI: 10.1073/pnas.1208507109.
(38) Garneau, J. E.; Dupuis, M. E.; Villion, M.; Romero, D. A.; Barrangou, R.; Boyaval, P.; Fremaux, C.; Horvath, P.; Magadan, A. H.; Moineau, S. The CRISPR/Cas Bacterial Immune System Cleaves Bacteriophage and Plasmid DNA. *Nature* **2010**, *468* (7320), 67-71. DOI: 10.1038/nature09523.
(39) Shmakov, S.; Abudayyeh, O. O.; Makarova, K. S.; Wolf, Y. I.; Gootenberg, J. S.; Semenova, E.; Minakhin, L.; Joung, J.; Konermann, S.; Severinov, K.; et al. Discovery and Functional Characterization of Diverse Class 2 CRISPR-Cas Systems. *Mol. Cell* **2015**, *60* (3), 385-397. DOI: 10.1016/j.molcel.2015.10.008.
(40) Shmakov, S.; Smargon, A.; Scott, D.; Cox, D.; Pyzocha, N.; Yan, W.; Abudayyeh, O. O.; Gootenberg, J. S.; Makarova, K. S.; Wolf, Y. I.; et al. Diversity and Evolution of Class 2 CRISPR-Cas Systems. *Nat. Rev. Microbiol.* **2017**, *15* (3), 169-182. DOI: 10.1038/nrmicro.2016.184.
(41) Hryhorowicz, M.; Lipinski, D.; Zeyland, J.; Slomski, R. CRISPR/Cas9 Immune System as a Tool for Genome Engineering. *Arch. Immunol. Ther. Exp. (Warsz)* **2017**, *65* (3), 233-240. DOI: 10.1007/s00005-016-0427-5.
(42) Qi, L. S.; Larson, M. H.; Gilbert, L. A.; Doudna, J. A.; Weissman, J. S.; Arkin, A. P.; Lim, W. A. Repurposing CRISPR as An RNA-guided Platform for Sequence-specific Control of Gene Expression. *Cell* **2013**, *152* (5), 1173-1183. DOI: 10.1016/j.cell.2013.02.022.
(43) Anzalone, A. V.; Koblan, L. W.; Liu, D. R. Genome Editing with CRISPR-Cas Nucleases, Base Editors, Transposases and Prime Editors. *Nat. Biotechnol.* **2020**, *38* (7), 824-844. DOI: 10.1038/s41587-020-0561-9.
(44) Nakamura, M.; Gao, Y.; Dominguez, A. A.; Qi, L. S. CRISPR Technologies for Precise Epigenome Editing. *Nat. Cell Biol.* **2021**, *23* (1), 11-22. DOI: 10.1038/s41556-020-00620-7.
(45) Wang, H.; La Russa, M.; Qi, L. S. CRISPR/Cas9 in Genome Editing and Beyond. *Annu. Rev. Biochem.* **2016**, *85*, 227-264. DOI: 10.1146/annurev-biochem-060815-014607.
(46) Swarts, D. C.; Jinek, M. Mechanistic Insights into the cis- and trans-Acting DNase Activities of Cas12a. *Mol. Cell* **2019**, *73* (3), 589-600 e584. DOI: 10.1016/j.molcel.2018.11.021 From NLM Medline.
(47) Zetsche, B.; Gootenberg, J. S.; Abudayyeh, O. O.; Slaymaker, I. M.; Makarova, K. S.; Essletzbichler, P.; Volz, S. E.; Joung, J.; van der Oost, J.; Regev, A.; et al. Cpf1 is a Single RNA-guided Endonuclease of a Class 2 CRISPR-Cas System. *Cell* **2015**, *163* (3), 759-771. DOI: 10.1016/j.cell.2015.09.038.
(48) Aquino-Jarquin, G. CRISPR-Cas14 is Now Part of the Artillery for Gene Editing and Molecular Diagnostic. *Nanomedicine* **2019**, *18*, 428-431. DOI: 10.1016/j.nano.2019.03.006.
(49) Yuan, B.; Yuan, C.; Li, L.; Long, M.; Chen, Z. Application of the CRISPR/Cas System in Pathogen Detection: A Review. *Molecules* **2022**, *27* (20). DOI: 10.3390/molecules27206999.
(50) Karvelis, T.; Bigelyte, G.; Young, J. K.; Hou, Z.; Zedaveinyte, R.; Budre, K.; Paulraj, S.; Djukanovic, V.; Gasior, S.; Silanskas, A.; et al. PAM recognition by miniature CRISPR-Cas12f nucleases triggers programmable double-stranded DNA target cleavage. *Nucleic Acids Res* **2020**, *48* (9), 5016-5023. DOI: 10.1093/nar/gkaa208 From NLM Medline.
(51) Anantharaman, V.; Makarova, K. S.; Burroughs, A. M.; Koonin, E. V.; Aravind, L. Comprehensive Analysis of the HEPN Superfamily: Identification of Novel Roles in Intra-

Genomic Conflicts, Defense, Pathogenesis and RNA Processing. *Biol. Direct.* **2013**, *8*, 15. DOI: 10.1186/1745-6150-8-15.
(52) Abudayyeh, O. O.; Gootenberg, J. S.; Essletzbichler, P.; Han, S.; Joung, J.; Belanto, J. J.; Verdine, V.; Cox, D. B. T.; Kellner, M. J.; Regev, A.; et al. RNA Targeting with CRISPR-Cas13. *Nature* **2017**, *550* (7675), 280-284. DOI: 10.1038/nature24049.
(53) Cox, D. B. T.; Gootenberg, J. S.; Abudayyeh, O. O.; Franklin, B.; Kellner, M. J.; Joung, J.; Zhang, F. RNA Editing with CRISPR-Cas13. *Science* **2017**, *358* (6366), 1019-1027. DOI: 10.1126/science.aaq0180.
(54) O'Connell, M. R. Molecular Mechanisms of RNA Targeting by Cas13-containing Type VI CRISPR-Cas Systems. *J. Mol. Biol.* **2019**, *431* (1), 66-87. DOI: 10.1016/j.jmb.2018.06.029.
(55) *Regeneron and Intellia Therapeutics Expand Collaboration to Develop CRISPR/Cas9-Based Treatments*. 2020. https://investor.regeneron.com/news-releases/news-release-details/regeneron-and-intellia-therapeutics-expand-collaboration-develop (accessed 2024 October 28th).
(56) *Hemophilia A*. https://rarediseases.org/rare-diseases/hemophilia-a/ (accessed 2024 October 28th).
(57) *Regeneron and Mammoth Biosciences Collaborate to Pursue Next-Generation CRISPR-Based Gene Editing for Multiple Diseases*. 2024. https://investor.regeneron.com/news-releases/news-release-details/regeneron-and-mammoth-biosciences-collaborate-pursue-next (accessed 2024 October 28th).
(58) Li, J.-F.; Norville, J. E.; Aach, J.; McCormack, M.; Zhang, D.; Bush, J.; Church, G. M.; Sheen, J. Multiplex and Homologous Recombination–mediated Genome Editing in *Arabidopsis* and *Nicotiana benthamiana* Using Guide RNA and Cas9. *Nat. Biotechnol.* **2013**, *31* (8), 688-691. DOI: 10.1038/nbt.2654.
(59) Shan, Q.; Wang, Y.; Li, J.; Zhang, Y.; Chen, K.; Liang, Z.; Zhang, K.; Liu, J.; Xi, J. J.; Qiu, J.-L.; et al. Targeted Genome Modification of Crop Plants Using a CRISPR-Cas System. *Nat. Biotechnol.* **2013**, *31* (8), 686-688. DOI: 10.1038/nbt.2650.
(60) Nekrasov, V.; Staskawicz, B.; Weigel, D.; Jones, J. D. G.; Kamoun, S. Targeted Mutagenesis in the Model Plant *Nicotiana benthamiana* Using Cas9 RNA-guided Endonuclease. *Nat. Biotechnol.* **2013**, *31* (8), 691-693. DOI: 10.1038/nbt.2655.
(61) Jhu, M.-Y.; Ellison, E. E.; Sinha, N. R. CRISPR Gene Editing to Improve Crop Resistance to Parasitic Plants. *Front. Genome Ed.* **2023**, *5*, Mini Review. DOI: 10.3389/fgeed.2023.1289416.
(62) Paul, N. C.; Park, S.-W.; Liu, H.; Choi, S.; Ma, J.; MacCready, J. S.; Chilvers, M. I.; Sang, H. Plant and Fungal Genome Editing to Enhance Plant Disease Resistance Using the CRISPR/Cas9 System. *Front. Plant Sci.* **2021**, *12*, 700925, Mini Review. DOI: 10.3389/fpls.2021.700925.
(63) Khan, Z. A.; Kumar, R.; Dasgupta, I. CRISPR/Cas-Mediated Resistance against Viruses in Plants. In *Int. J. Mol. Sci.*, 2022; Vol. 23, p 2303.
(64) Zaidi, S. S.; Mahas, A.; Vanderschuren, H.; Mahfouz, M. M. Engineering Crops of the Future: CRISPR Approaches to Develop Climate-resilient and Disease-resistant Plants. *Genome Biol.* **2020**, *21* (1), 289. DOI: 10.1186/s13059-020-02204-y.
(65) Karavolias, N. G.; Horner, W.; Abugu, M. N.; Evanega, S. N. Application of Gene Editing for Climate Change in Agriculture. *Front. Sustain. Food Syst.* **2021**, *5*, Systematic Review. DOI: 10.3389/fsufs.2021.685801.
(66) Ndudzo, A.; Sibanda Makuvise, A.; Moyo, S.; Bobo, E. D. CRISPR-Cas9 Genome Editing in Crop Breeding for Climate Change Resilience: Implications for Smallholder Farmers in Africa. *J. Agric. Food Res.* **2024**, *16*, 101132. DOI: 10.1016/j.jafr.2024.101132.
(67) Misra, V.; Pandey, H.; Mall, A. K. Exploring CRISPR/Cas9 Gene Editing Applications for Enhancing Disease Resistance in Sugar Beet. *ACS Agric. Sci. Technol.* **2024**, *4* (1), 4-21. DOI: 10.1021/acsagscitech.3c00193.

(68) Schenke, D.; Cai, D. Applications of CRISPR/Cas to Improve Crop Disease Resistance: Beyond Inactivation of Susceptibility Factors. *iScience* **2020**, *23* (9), 101478. DOI: 10.1016/j.isci.2020.101478.
(69) Ahmad, S.; Tang, L.; Shahzad, R.; Mawia, A. M.; Rao, G. S.; Jamil, S.; Wei, C.; Sheng, Z.; Shao, G.; Wei, X.; et al. CRISPR-Based Crop Improvements: A Way Forward to Achieve Zero Hunger. *J. Agric. Food Chem.* **2021**, *69* (30), 8307-8323. DOI: 10.1021/acs.jafc.1c02653.
(70) Maximiano, M. R.; Franco, O. L. CRISPR/Cas: The New Frontier in Plant Improvement. *ACS Agric. Sci. Technol.* **2022**, *2* (2), 202-214. DOI: 10.1021/acsagscitech.1c00279.
(71) Xue, L.; Tang, B.; Chen, W.; Luo, J. Prediction of CRISPR sgRNA Activity Using a Deep Convolutional Neural Network. *J. Chem. Inf. Model.* **2019**, *59* (1), 615-624. DOI: 10.1021/acs.jcim.8b00368.
(72) Ruffolo, J. A.; Nayfach, S.; Gallagher, J.; Bhatnagar, A.; Beazer, J.; Hussain, R.; Russ, J.; Yip, J.; Hill, E.; Pacesa, M.; et al. [Preprint] Design of highly functional genome editors by modeling the universe of CRISPR-Cas sequences. *bioRxiv* **2024**. DOI: 10.1101/2024.04.22.590591.
(73) Nijkamp, E.; Ruffolo, J. A.; Weinstein, E. N.; Naik, N.; Madani, A. ProGen2: Exploring the Boundaries of Protein Language Models. *Cell Syst.* **2023**, *14* (11), 968-978. DOI: 10.1016/j.cels.2023.10.002.
(74) Wessels, H. H.; Stirn, A.; Mendez-Mancilla, A.; Kim, E. J.; Hart, S. K.; Knowles, D. A.; Sanjana, N. E. Prediction of On-target and Off-target Activity of CRISPR-Cas13d Guide RNAs Using Deep Learning. *Nat. Biotechnol.* **2024**, *42* (4), 628-637. DOI: 10.1038/s41587-023-01830-8.
(75) Nguyen, E.; Poli, M.; Durrant, M. G.; Kang, B.; Katrekar, D.; Li, D. B.; Bartie, L. J.; Thomas, A. W.; King, S. H.; Brixi, G.; et al. Sequence Modeling and Design from Molecular to Genome Scale with Evo. *Science* **2024**, *386* (6723), eado9336. DOI: 10.1126/science.ado9336.
(76) *CRISPick*. https://portals.broadinstitute.org/gppx/crispick/public (accessed 2024 October 30th).
(77) Doench, J. G.; Fusi, N.; Sullender, M.; Hegde, M.; Vaimberg, E. W.; Donovan, K. F.; Smith, I.; Tothova, Z.; Wilen, C.; Orchard, R.; et al. Optimized sgRNA Design to Maximize Activity and Minimize Off-target Effects of CRISPR-Cas9. *Nat. Biotechnol.* **2016**, *34* (2), 184-191. DOI: 10.1038/nbt.3437.
(78) Sanson, K. R.; Hanna, R. E.; Hegde, M.; Donovan, K. F.; Strand, C.; Sullender, M. E.; Vaimberg, E. W.; Goodale, A.; Root, D. E.; Piccioni, F.; et al. Optimized Libraries for CRISPR-Cas9 Genetic Screens with Multiple Modalities. *Nat. Commun.* **2018**, *9* (1), 5416. DOI: 10.1038/s41467-018-07901-8.
(79) *sgRNA Scorer*. https://frederick.cancer.gov/resources/repositories/sgrnascorer (accessed 2024 October 30th).
(80) Chari, R.; Yeo, N. C.; Chavez, A.; Church, G. M. sgRNA Scorer 2.0: A Species-Independent Model To Predict CRISPR/Cas9 Activity. *ACS Synth. Biol.* **2017**, *6* (5), 902-904. DOI: 10.1021/acssynbio.6b00343.
(81) *Welcome to SSC - Sequence Scan for CRISPR*. http://crispr.dfci.harvard.edu/SSC/ (accessed 2024 October 30th).
(82) Xu, H.; Xiao, T.; Chen, C. H.; Li, W.; Meyer, C. A.; Wu, Q.; Wu, D.; Cong, L.; Zhang, F.; Liu, J. S.; et al. Sequence Determinants of Improved CRISPR sgRNA Design. *Genome Res.* **2015**, *25* (8), 1147-1157. DOI: 10.1101/gr.191452.115.
(83) *DeepCRISPR*. http://www.deepcrispr.net/ (accessed 2024 October 30th).
(84) Chuai, G.; Ma, H.; Yan, J.; Chen, M.; Hong, N.; Xue, D.; Zhou, C.; Zhu, C.; Chen, K.; Duan, B.; et al. DeepCRISPR: Optimized CRISPR Guide RNA Design by Deep Learning. *Genome Biol.* **2018**, *19* (1), 80. DOI: 10.1186/s13059-018-1459-4.

(85) Abadi, S.; Yan, W. X.; Amar, D.; Mayrose, I. A Machine Learning Approach for Predicting CRISPR-Cas9 Cleavage Efficiencies and Patterns Underlying its Mechanism of Action. *PLoS Comput. Biol.* **2017**, *13* (10), e1005807. DOI: 10.1371/journal.pcbi.1005807.
(86) Prykhozhij, S. V.; Rajan, V.; Gaston, D.; Berman, J. N. CRISPR Multitargeter: A Web Tool to Find Common and Unique CRISPR Single Guide RNA Targets in a Set of Similar Sequences. *PLoS One* **2015**, *10* (3), e0119372. DOI: 10.1371/journal.pone.0119372.
(87) Prykhozhij, S. V.; Rajan, V.; Gaston, D.; Berman, J. N. Correction: CRISPR MultiTargeter: A Web Tool to Find Common and Unique CRISPR Single Guide RNA Targets in a Set of Similar Sequences. *PLoS One* **2015**, *10* (9), e0138634. DOI: 10.1371/journal.pone.0138634.
(88) *DeepHF*. http://www.deephf.com/#/home (accessed 2024 October 30th).
(89) Wang, D.; Zhang, C.; Wang, B.; Li, B.; Wang, Q.; Liu, D.; Wang, H.; Zhou, Y.; Shi, L.; Lan, F.; et al. Optimized CRISPR Guide RNA Design for Two High-fidelity Cas9 Variants by Deep Learning. *Nat. Commun.* **2019**, *10* (1), 4284. DOI: 10.1038/s41467-019-12281-8.
(90) Zheng, X.; Cui, J.; Wang, Y.; Zhang, J.; Wang, C. [Preprint] CRSIPR-A-I: A Webtool for the Efficacy Prediction of CRISPR Activation and Interference. *bioRxiv* **2021**. DOI: 10.1101/2021.12.02.470943.
(91) *CRISPR-P 2.0: An Improved CRISPR/Cas9 Tool for Genome Editing in Plants*. http://crispr.hzau.edu.cn/CRISPR2/ (accessed 2024 October 30th).
(92) Lei, Y.; Lu, L.; Liu, H. Y.; Li, S.; Xing, F.; Chen, L. L. CRISPR-P: A Web Tool for Synthetic Single-guide RNA Design of CRISPR-system in Plants. *Mol. Plant* **2014**, *7* (9), 1494-1496. DOI: 10.1093/mp/ssu044.
(93) Dixit, S.; Kumar, A.; Srinivasan, K.; Vincent, P.; Ramu Krishnan, N. Advancing Genome Editing with Artificial Intelligence: Opportunities, Challenges, and Future Directions. *Front. Bioeng. Biotechnol.* **2023**, *11*, 1335901. DOI: 10.3389/fbioe.2023.1335901.
(94) Lee, M. Deep Learning in CRISPR-Cas Systems: A Review of Recent Studies. *Front. Bioeng. Biotechnol.* **2023**, *11*, 1226182. DOI: 10.3389/fbioe.2023.1226182.
(95) Chiang, T. W.; le Sage, C.; Larrieu, D.; Demir, M.; Jackson, S. P. CRISPR-Cas9(D10A) Nickase-based Genotypic and Phenotypic Screening to Enhance Genome Editing. *Sci. Rep.* **2016**, *6*, 24356. DOI: 10.1038/srep24356.
(96) Kanafi, M. M.; Tavallaei, M. Overview of Advances in CRISPR/deadCas9 Technology and its Applications in Human Diseases. *Gene* **2022**, *830*, 146518. DOI: 10.1016/j.gene.2022.146518.
(97) Zhang, Y.; Qian, L.; Wei, W.; Wang, Y.; Wang, B.; Lin, P.; Liu, W.; Xu, L.; Li, X.; Liu, D.; et al. Paired Design of dCas9 as a Systematic Platform for the Detection of Featured Nucleic Acid Sequences in Pathogenic Strains. *ACS Synth. Biol.* **2017**, *6* (2), 211-216. DOI: 10.1021/acssynbio.6b00215.
(98) Liu, P.; Lin, Y.; Zhuo, X.; Zeng, J.; Chen, B.; Zou, Z.; Liu, G.; Xiong, E.; Yang, R. Universal crRNA Acylation Strategy for Robust Photo-Initiated One-Pot CRISPR-Cas12a Nucleic Acid Diagnostics. *Angew Chem. Int. Ed. Engl.* **2024**, *63* (23), e202401486. DOI: 10.1002/anie.202401486.
(99) Xiong, Y.; Zhang, J.; Yang, Z.; Mou, Q.; Ma, Y.; Xiong, Y.; Lu, Y. Functional DNA Regulated CRISPR-Cas12a Sensors for Point-of-Care Diagnostics of Non-Nucleic-Acid Targets. *J. Am. Chem. Soc.* **2020**, *142* (1), 207-213. DOI: 10.1021/jacs.9b09211.
(100) Wang, Z.; Zhong, C. Cas12c-DETECTOR: A Specific and Sensitive Cas12c-based DNA Detection Platform. *Int. J. Biol. Macromol.* **2021**, *193* (Pt A), 441-449. DOI: 10.1016/j.ijbiomac.2021.10.167.
(101) Abudayyeh, O. O.; Gootenberg, J. S.; Konermann, S.; Joung, J.; Slaymaker, I. M.; Cox, D. B.; Shmakov, S.; Makarova, K. S.; Semenova, E.; Minakhin, L.; et al. C2c2 is a Single-Component Programmable RNA-Guided RNA-Targeting CRISPR Effector. *Science* **2016**, *353* (6299), aaf5573. DOI: 10.1126/science.aaf5573.

(102) Harrington, L. B.; Burstein, D.; Chen, J. S.; Paez-Espino, D.; Ma, E.; Witte, I. P.; Cofsky, J. C.; Kyrpides, N. C.; Banfield, J. F.; Doudna, J. A. Programmed DNA Destruction by Miniature CRISPR-Cas14 Enzymes. *Science* **2018**, *362* (6416), 839-842. DOI: 10.1126/science.aav4294.
(103) Pardee, K.; Green, A. A.; Takahashi, M. K.; Braff, D.; Lambert, G.; Lee, J. W.; Ferrante, T.; Ma, D.; Donghia, N.; Fan, M.; et al. Rapid, Low-Cost Detection of Zika Virus Using Programmable Biomolecular Components. *Cell* **2016**, *165* (5), 1255-1266. DOI: 10.1016/j.cell.2016.04.059 From NLM Medline.
(104) Huang, M.; Zhou, X.; Wang, H.; Xing, D. Clustered Regularly Interspaced Short Palindromic Repeats/Cas9 Triggered Isothermal Amplification for Site-Specific Nucleic Acid Detection. *Anal Chem* **2018**, *90* (3), 2193-2200. DOI: 10.1021/acs.analchem.7b04542 From NLM Medline.
(105) Quan, J.; Langelier, C.; Kuchta, A.; Batson, J.; Teyssier, N.; Lyden, A.; Caldera, S.; McGeever, A.; Dimitrov, B.; King, R.; et al. FLASH: a next-generation CRISPR diagnostic for multiplexed detection of antimicrobial resistance sequences. *Nucleic Acids Res* **2019**, *47* (14), e83. DOI: 10.1093/nar/gkz418 From NLM Medline.
(106) Azhar, M.; Phutela, R.; Kumar, M.; Ansari, A. H.; Rauthan, R.; Gulati, S.; Sharma, N.; Sinha, D.; Sharma, S.; Singh, S.; et al. Rapid and accurate nucleobase detection using FnCas9 and its application in COVID-19 diagnosis. *Biosens Bioelectron* **2021**, *183*, 113207. DOI: 10.1016/j.bios.2021.113207 From NLM Medline.
(107) Wang, X.; Xiong, E.; Tian, T.; Cheng, M.; Lin, W.; Wang, H.; Zhang, G.; Sun, J.; Zhou, X. Clustered Regularly Interspaced Short Palindromic Repeats/Cas9-Mediated Lateral Flow Nucleic Acid Assay. *ACS Nano* **2020**, *14* (2), 2497-2508. DOI: 10.1021/acsnano.0c00022 From NLM Medline.
(108) Guk, K.; Keem, J. O.; Hwang, S. G.; Kim, H.; Kang, T.; Lim, E. K.; Jung, J. A facile, rapid and sensitive detection of MRSA using a CRISPR-mediated DNA FISH method, antibody-like dCas9/sgRNA complex. *Biosens Bioelectron* **2017**, *95*, 67-71. DOI: 10.1016/j.bios.2017.04.016 From NLM Medline.
(109) Zhang, Y.; Qian, L.; Wei, W.; Wang, Y.; Wang, B.; Lin, P.; Liu, W.; Xu, L.; Li, X.; Liu, D.; et al. Paired Design of dCas9 as a Systematic Platform for the Detection of Featured Nucleic Acid Sequences in Pathogenic Strains. *ACS Synth Biol* **2017**, *6* (2), 211-216. DOI: 10.1021/acssynbio.6b00215 From NLM Medline.
(110) Wang, M.; Chen, K.; Wu, Q.; Peng, R.; Zhang, R.; Li, J. RCasFISH: CRISPR/dCas9-Mediated in Situ Imaging of mRNA Transcripts in Fixed Cells and Tissues. *Anal Chem* **2020**, *92* (3), 2468-2475. DOI: 10.1021/acs.analchem.9b03797 From NLM Medline.
(111) Zhou, W.; Hu, L.; Ying, L.; Zhao, Z.; Chu, P. K.; Yu, X. F. A CRISPR-Cas9-triggered strand displacement amplification method for ultrasensitive DNA detection. *Nat Commun* **2018**, *9* (1), 5012. DOI: 10.1038/s41467-018-07324-5 From NLM Medline.
(112) Wang, T.; Liu, Y.; Sun, H. H.; Yin, B. C.; Ye, B. C. An RNA-Guided Cas9 Nickase-Based Method for Universal Isothermal DNA Amplification. *Angew Chem Int Ed Engl* **2019**, *58* (16), 5382-5386. DOI: 10.1002/anie.201901292 From NLM Medline.
(113) Chen, J. S.; Ma, E.; Harrington, L. B.; Da Costa, M.; Tian, X.; Palefsky, J. M.; Doudna, J. A. CRISPR-Cas12a target binding unleashes indiscriminate single-stranded DNase activity. *Science* **2018**, *360* (6387), 436-439. DOI: 10.1126/science.aar6245 From NLM Medline.
(114) Wang, B.; Wang, R.; Wang, D.; Wu, J.; Li, J.; Wang, J.; Liu, H.; Wang, Y. Cas12aVDet: A CRISPR/Cas12a-Based Platform for Rapid and Visual Nucleic Acid Detection. *Anal Chem* **2019**, *91* (19), 12156-12161. DOI: 10.1021/acs.analchem.9b01526 From NLM Medline.
(115) Liu, P.; Lin, Y.; Zhuo, X.; Zeng, J.; Chen, B.; Zou, Z.; Liu, G.; Xiong, E.; Yang, R. Universal crRNA Acylation Strategy for Robust Photo-Initiated One-Pot CRISPR-Cas12a Nucleic Acid Diagnostics. *Angew Chem Int Ed Engl* **2024**, *63* (23), e202401486. DOI: 10.1002/anie.202401486 From NLM Medline.

(116) Li, S. Y.; Cheng, Q. X.; Wang, J. M.; Li, X. Y.; Zhang, Z. L.; Gao, S.; Cao, R. B.; Zhao, G. P.; Wang, J. CRISPR-Cas12a-assisted nucleic acid detection. *Cell Discov* **2018**, *4*, 20. DOI: 10.1038/s41421-018-0028-z From NLM PubMed-not-MEDLINE.
(117) Nouri, R.; Jiang, Y.; Lian, X. L.; Guan, W. Sequence-Specific Recognition of HIV-1 DNA with Solid-State CRISPR-Cas12a-Assisted Nanopores (SCAN). *ACS Sens* **2020**, *5* (5), 1273-1280. DOI: 10.1021/acssensors.0c00497 From NLM Medline.
(118) Dai, Y.; Somoza, R. A.; Wang, L.; Welter, J. F.; Li, Y.; Caplan, A. I.; Liu, C. C. Exploring the Trans-Cleavage Activity of CRISPR-Cas12a (cpf1) for the Development of a Universal Electrochemical Biosensor. *Angew Chem Int Ed Engl* **2019**, *58* (48), 17399-17405. DOI: 10.1002/anie.201910772 From NLM Medline.
(119) Xing, S.; Lu, Z.; Huang, Q.; Li, H.; Wang, Y.; Lai, Y.; He, Y.; Deng, M.; Liu, W. An ultrasensitive hybridization chain reaction-amplified CRISPR-Cas12a aptasensor for extracellular vesicle surface protein quantification. *Theranostics* **2020**, *10* (22), 10262-10273. DOI: 10.7150/thno.49047 From NLM Medline.
(120) Liang, M.; Li, Z.; Wang, W.; Liu, J.; Liu, L.; Zhu, G.; Karthik, L.; Wang, M.; Wang, K. F.; Wang, Z.; et al. A CRISPR-Cas12a-derived biosensing platform for the highly sensitive detection of diverse small molecules. *Nat Commun* **2019**, *10* (1), 3672. DOI: 10.1038/s41467-019-11648-1 From NLM Medline.
(121) Xiong, Y.; Zhang, J.; Yang, Z.; Mou, Q.; Ma, Y.; Xiong, Y.; Lu, Y. Functional DNA Regulated CRISPR-Cas12a Sensors for Point-of-Care Diagnostics of Non-Nucleic-Acid Targets. *J Am Chem Soc* **2020**, *142* (1), 207-213. DOI: 10.1021/jacs.9b09211 From NLM Medline.
(122) Tang, Y.; Song, T.; Gao, L.; Yin, S.; Ma, M.; Tan, Y.; Wu, L.; Yang, Y.; Wang, Y.; Lin, T.; et al. A CRISPR-based ultrasensitive assay detects attomolar concentrations of SARS-CoV-2 antibodies in clinical samples. *Nat Commun* **2022**, *13* (1), 4667. DOI: 10.1038/s41467-022-32371-4 From NLM Medline.
(123) Li, L.; Li, S.; Wu, N.; Wu, J.; Wang, G.; Zhao, G.; Wang, J. HOLMESv2: A CRISPR-Cas12b-Assisted Platform for Nucleic Acid Detection and DNA Methylation Quantitation. *ACS Synth Biol* **2019**, *8* (10), 2228-2237. DOI: 10.1021/acssynbio.9b00209 From NLM Medline.
(124) Teng, F.; Guo, L.; Cui, T.; Wang, X. G.; Xu, K.; Gao, Q.; Zhou, Q.; Li, W. CDetection: CRISPR-Cas12b-based DNA detection with sub-attomolar sensitivity and single-base specificity. *Genome Biol* **2019**, *20* (1), 132. DOI: 10.1186/s13059-019-1742-z From NLM Medline.
(125) Wang, Z.; Zhong, C. Cas12c-DETECTOR: A specific and sensitive Cas12c-based DNA detection platform. *Int J Biol Macromol* **2021**, *193* (Pt A), 441-449. DOI: 10.1016/j.ijbiomac.2021.10.167 From NLM Medline.
(126) Gootenberg, J. S.; Abudayyeh, O. O.; Lee, J. W.; Essletzbichler, P.; Dy, A. J.; Joung, J.; Verdine, V.; Donghia, N.; Daringer, N. M.; Freije, C. A.; et al. Nucleic acid detection with CRISPR-Cas13a/C2c2. *Science* **2017**, *356* (6336), 438-442. DOI: 10.1126/science.aam9321 From NLM Medline.
(127) Gootenberg, J. S.; Abudayyeh, O. O.; Kellner, M. J.; Joung, J.; Collins, J. J.; Zhang, F. Multiplexed and portable nucleic acid detection platform with Cas13, Cas12a, and Csm6. *Science* **2018**, *360* (6387), 439-444. DOI: 10.1126/science.aaq0179 From NLM Medline.
(128) Myhrvold, C.; Freije, C. A.; Gootenberg, J. S.; Abudayyeh, O. O.; Metsky, H. C.; Durbin, A. F.; Kellner, M. J.; Tan, A. L.; Paul, L. M.; Parham, L. A.; et al. Field-deployable viral diagnostics using CRISPR-Cas13. *Science* **2018**, *360* (6387), 444-448. DOI: 10.1126/science.aas8836 From NLM Medline.
(129) Ackerman, C. M.; Myhrvold, C.; Thakku, S. G.; Freije, C. A.; Metsky, H. C.; Yang, D. K.; Ye, S. H.; Boehm, C. K.; Kosoko-Thoroddsen, T. F.; Kehe, J.; et al. Massively multiplexed nucleic acid detection with Cas13. *Nature* **2020**, *582* (7811), 277-282. DOI: 10.1038/s41586-020-2279-8 From NLM Medline.

(130) Wang, X.; Zhou, S.; Chu, C.; Yang, M.; Huo, D.; Hou, C. Dual Methylation-Sensitive Restriction Endonucleases Coupling with an RPA-Assisted CRISPR/Cas13a System (DESCS) for Highly Sensitive Analysis of DNA Methylation and Its Application for Point-of-Care Detection. *ACS Sens* **2021**, *6* (6), 2419-2428. DOI: 10.1021/acssensors.1c00674 From NLM Medline.
(131) Broto, M.; Kaminski, M. M.; Adrianus, C.; Kim, N.; Greensmith, R.; Dissanayake-Perera, S.; Schubert, A. J.; Tan, X.; Kim, H.; Dighe, A. S.; et al. Nanozyme-catalysed CRISPR assay for preamplification-free detection of non-coding RNAs. *Nat Nanotechnol* **2022**, *17* (10), 1120-1126. DOI: 10.1038/s41565-022-01179-0 From NLM Medline.
(132) Cui, J. Q.; Liu, F. X.; Park, H.; Chan, K. W.; Leung, T.; Tang, B. Z.; Yao, S. Droplet digital recombinase polymerase amplification (ddRPA) reaction unlocking via picoinjection. *Biosens Bioelectron* **2022**, *202*, 114019. DOI: 10.1016/j.bios.2022.114019 From NLM Medline.
(133) Shen, J.; Zhou, X.; Shan, Y.; Yue, H.; Huang, R.; Hu, J.; Xing, D. Sensitive detection of a bacterial pathogen using allosteric probe-initiated catalysis and CRISPR-Cas13a amplification reaction. *Nat Commun* **2020**, *11* (1), 267. DOI: 10.1038/s41467-019-14135-9 From NLM Medline.
(134) Iwasaki, R. S.; Batey, R. T. SPRINT: a Cas13a-based platform for detection of small molecules. *Nucleic Acids Res* **2020**, *48* (17), e101. DOI: 10.1093/nar/gkaa673 From NLM Medline.
(135) Chen, Q.; Tian, T.; Xiong, E.; Wang, P.; Zhou, X. CRISPR/Cas13a Signal Amplification Linked Immunosorbent Assay for Femtomolar Protein Detection. *Anal Chem* **2020**, *92* (1), 573-577. DOI: 10.1021/acs.analchem.9b04403 From NLM Medline.
